# Supplementary material for: Health outcomes associated with Zika virus infection in humans: a systematic review of systematic reviews
Source: BMJ Open. 2019 Nov 3;9(11):e032275. doi: 10.1136/bmjopen-2019-032275 (PMC6858219; doi:10.1136/bmjopen-2019-032275)
Supplement: Supplementary data [file bmjopen-2019-032275supp001.pdf]

Search Strategy

Database: Embase Classic+Embase <1947 to 2018 February 27>

Search Strategy:

1 zika fever/ or zika virus/ or zika virus vaccine/ or zika.mp. (5013)

2 "systematic review"/ or "review"/ (2367967)

3 1 and 2 (569)

Database: Ovid MEDLINE(R) <1946 to February Week 3 2018>

Search Strategy:

1 exp ZIKA VIRUS INFECTION/ or exp ZIKA VIRUS/ or zika.mp. (2287)

2 "review"/ (2215441)

3 1 and 2 (326)

Database: Cochrane

Search Strategy:

1 ZIKA and review (2)

Update – 22/07/2019

Database: LILACS

Search Strategy:

(tw:((tw:(ZIKA VIRUS INFECTION)) OR (tw:(ZIKA VIRUS)) OR (tw:(zika.mp)))) AND (tw:(systematic review)) (729)

Database(s): Ovid MEDLINE(R) 1946 to July Week 2 2019

Search Strategy:

1 exp ZIKA VIRUS INFECTION/ or exp ZIKA VIRUS/ or zika.mp. (4560)

2 "review"/ (2360456)

3 1 and 2 (722)

4 limit 3 to yr=”2018-Current” (261)

Database(s): Embase Classic+Embase <1947 to 2019 July 19>

Search Strategy:

1 zika fever/ or zika virus/ or zika virus vaccine/ or zika.mp. (8593)

2 "systematic review"/ or "review"/ (2553024)

3 1 and 2 (1056)

4 limit 3 to yr=”2018-Current” (504)

Database: Cochrane

Search Strategy:

1 ZIKA and review (0)
