# Supplementary material for: Health outcomes associated with Zika virus infection in humans: a systematic review of systematic reviews
Source: BMJ Open. 2019 Nov 3;9(11):e032275. doi: 10.1136/bmjopen-2019-032275 (PMC6858219; doi:10.1136/bmjopen-2019-032275)
Supplement: Supplementary data [file bmjopen-2019-032275supp002.pdf]

Supplementary file 2 - Table 1. Health outcomes - Congenital Zika syndrome

Authors	Malformations / Congenital abnormalities	Brain abnormalities	Microcephaly	Ocular disorders	Auditory disorder - Rate	Cardiovascular damage
Krauer et al. (2017) [15]		Prevalence: 96% of cases	91% of cases; Prevalence ratio over states with no reported cases of microcephaly= 4.67	Prevalence: 42% (49 cases in 116 mother-infant pairs)	13% (3 cases in 24 mother-infant pairs)	
Paixao et al. (2016) [16]		In 2015, the prevalence of microcephaly in Brazil was 20 cases per 10,000 live births; Zika infection during 9 pregnancies confirmed by CDC resulted in the birth of a neonate with microcephaly.	Rate per 100,000 live births: 121.7 (0.12%) in 2015 in Brazil; Death due microcephaly: 1.3% in suspected microcephaly cases			
Chibueze et al. (2017) [17]		In one observational study of 35 infants with microcephaly, 11 fetuses had intra-uterine brain injury accompanied by stunting of cerebral growth prior to birth.	One observational study provided a trimester-specific modelling estimate risk for microcephaly per 10,000 ZIKV infected pregnant women per trimester of pregnancy: 1st 95 (34 - 191), 2nd 84 (12 - 196), 3rd trimester: 0 - (0 - 251)			
Coelho et al. (2017) [18]	Other organs damage: French Guiana 2% in 250 live births or mother-infant pair. USA: 7%. Not clear if the denominator is the number of live births or mother-infant pair (301 or 498 respectively)		0.3% in live-birth pregnancies; 14.3% - in live-birth pregnancies; Prevalence (cases/all pregnancies): 2.3%. Prevalence (cases/live births): 2.7%. Death due to microcephaly: 8.3%, would be 5.7% in case of new confirmed cases are included.	Two studies reported a prevalence of ocular damage (0.9% and 1%). It is not clear if the denominator is the number of live births (395 and 301, respectively) or the number mother-infant pair (442 and 498, respectively)		French Guiana: Cardiovascular damage equal to 1%. The denominator is unclear if is the number of live births or mother-infant pair (301 and 498 respectively)
Simoes et al. (2016) [19]	Prevalence of CZS: 10 to 20 cases in 100,000 live births; 8.87% of cases with confirmed changes in CNS		The Ministry of Health in Brazil reported an increase in the number of cases of microcephaly close to 20 times that previously reported (aproximately 0.5 cases for each 10,000 live births) which means 10 microcephaly cases per 10,000 births.			
Padilla et al. (2016) [20]	In 72 women with Zika-positive serology during pregnancy in Brazil, 29% had abnormalities detected on fetal ultrasound. Central nervous system abnormalities were noted after Zika infections as late as 27 weeks' gestation, and placental insufficiency was noted with even later gestational ages.		In 2015, the prevalence of microcephaly in Brazil was 20 cases per 10,000 live births; Zika infection during 9 pregnancies confirmed by CDC resulted in the birth of a neonate with microcephaly.			
Marques et al. (2019) [21]		% of neurological malformations: Subcortical-cortical junction calcifications: 92.9%, Basal ganglia calcifications: 57.1%, Periventricular calcifications: 29.5%. Ventriculomegaly/hydrocephaly: 63.1%. Cerebellar abnormalities: 46.2%, 82% (14 of 17 patients). Corpus callosum abnormalities: 47.9%	39.7% in cases of congenital Zika infection. Almost 100% when the infection occurred during the first trimester and decreased when the infection occurred in the second or third trimester	Prevalence: 44.3% in congenital ZIKV infection, 20% in patients with microcephaly, 33% in patients with ventriculomegaly, and 43% in patients with calcification. Bilateral findings: 76.8% of infants with ocular lesions. In eyes of infants with ocular lesions and congenital ZIKV infection: Macular lesions in 50%, Optical nerve abnormalities: 27.78%, Chorioretinal artrophy/scaring: 10.65%, Focal pigment mottling of retina: 6.94%, Microphtalmia: 3.70%, Glaucoma: 2.31%, Cataract: 2.31%, Iris coloboma: 2.31%, Subluxation: 1.39%		
Counotte et al. (2018) [22]	Prevalence of adverse congenital outcomes: 8.97-49.57% in ZIKV positive women. Birth defects: 5.9% in pregnant asymptomatic women and 5.98% in symptomatic pregnant women		RR between ZIKV exposed and unexposed: 4.4-6.6. OR between women with confirmed ZIKV and without evidence of ZIKV infection: 11.0-55.5			
Haby et al. (2018) [23]			Prevalence of asymptomatic ZIKV infection in mothers who gave birth to babies with microcephaly: 0.36			

Authors	Malformations / Congenital abnormalities	Brain abnormalities	Microcephaly	Ocular disorders	Auditory disorder - Rate	Cardiovascular damage
**Sarwar et al. (2018) [24]**		Prevalence in dead neonates of ZIKV infected mothers: Pachygyria: 14.28%, Arthrogryposis: 14.28%. Morphologic microcephalus changes: 14.28%. Ventriculomegaly / hydrocephaly: 100%. Cerebellar abnormalities: 28.57%	Risk of 1% when infection occurred in the first trimester of pregnancy	In ZIKV infected infants: Retinal impairment: 28%, Impaired optic nerve: 17%, Optic nerve hypoplasia: 8%		
**Wahid et al. (2018) [25]**	Fetal abnormalities 28.57% in infected pregnant women. Ventricular calcifications or other central nervous system abnormal amniotic fluid volume or cerebral or umbilical artery flow: 16.67%. (CNS) lesions: 16.67%. 80 of the 185 infants, ZIKV-linked microcephaly: 10 (the value of the denominator is not clear) neonates, 5 of 80 or 185 birth defects such as hydranencephaly, holoprosencephaly, clubfeet, and craniosynostosis, 3 of 80 or 185: cataracts, holoprosencephaly, and ventral pons hypoplasia	Prevalence: 28% (including microcephaly) in newborns of mothers infected with ZIKV	Risk of microcephaly: 0-30%. Relative Risk 100–1,000 (assuming 10% exposure) or 20–200 (assuming 50% exposure) compared to background risk of microcephaly. Prevalence: 50.47% among definite or probable ZIKV cases. Higher risk of microcephaly in pregnant women infected during first trimester. Estimated risk of microcephaly: 0.95% in women infected in the first trimester	In infants with microcephaly: ophthalmoscopic alterations in 50% (not clear if ZIKV-related infection) . Ocular findings 34.5-58.62% of ZIKV linked microcephalic infants		
**Soriano-Arandes et al. (2018) [26]**	Birth defects: 6% in asymptomatic and symptomatic pregnant women. From 1 study: Fetal adverse outcomes in women infected with ZIKV: 55% in the first term of pregnancy, 29% in the third trimester. In infants with CZS: Dimples: 30.1%, Distal hand/finger contractures: 20.5%, feet malposition: 15.7%, generalized arthrogryposis: 9.6%, birth defects in women with recent ZIKV infection: 6%	Prevalence: Microcephaly in 86.7% and craniofacial disproportion in 95.8% of infants with probable CZS	In infected women in the first trimester: Risk of 0.95% in a population with an estimated rate of ZIKV infection of 66%; Prevalence of 55% in Rio de Janeiro. infection in the 3rd trimester: Prevalence: 29% (Rio de Janeiro). In a series of 13 infants with congenital ZIKV infection and progressive microcephaly, more than half of the mothers did not report any symptoms prior to delivery.		In a study of 70 children with microcephaly and laboratory diagnosys of congenital ZIKV infection, 5 (7%) had sensorineural hearing loss.	One study: congenital heart disease was described in 14 of a series of 103 cases (13.6%) in children with CZS.
**Santos et al. (2018) [28]**		Intracranial calcifitcation: 23 of 23 childen. Frontal lobe: 69% - 78%. Parietal lobe: 83% - 87%. Corticomedullary junction: 53% - 86%. Thalamus: 39% - 43%. Punctate calcification: 72% - 100%. Distributed in the band format: 56% - 75%. Reduction in the constitution of gyri of the severe cerebral cortex: 0.78. Cerebellar hypoplasia: 0.74. Involving only one cerebellar hemisphere : 13%. Brainstem globally hypoplastic: 8.7%. Abnormal hypodensity of the white matter: 1. Diffuse involvement of all the cerebral lobes: 0.87. Basal ganglia calcification: 57% - 65%				
**Pomar et al. (2019) [30]**	CZS: 4-9% of pregnancies of women infected by ZIKV. Malformations of cortical development: 79-82% of CZS cases. Intraventicular synechiae and periventricular cystic degeneration: 58% of CZS cases. Malformations of the corpus callosum: 71-100%. Vermian hypoplasia: 42% of CZS cases. 21% to 82%. Swallowing disorders and hydramnios: 25%. Partial immobilization or arthrogryposes: 10-25%. Motor abnormalities : 77.3-100% of CZS cases. Adverse outcomes - No signs/complications: 45% of proven infected fetuses/newborn. Adverse outcomes - Mild / moderate signs: 20% of proven infected fetuses/newborn. Adverse outcomes - Severe complications: 21% of proven infected fetuses/newborn. Risk of neurodevelopmental abnormality: 9% of infants born from infected mothers	Brain volume loss: 92%. Ventriculomegaly in CZS: 63.1-92%. Calcifications in CZS: 71-92%	Prevalence of microcephaly in CZS: 33.3-64%	Eye abnormalities: 25% in infants with CZS		

Authors	Malformations / Congenital abnormalities	Brain abnormalities	Microcephaly	Ocular disorders	Auditory disorder - Rate	Cardiovascular damage
**Wilder-Smith et al. (2018) [31]**	From infected pregnant travelers: Fetuses or infants with birth defects: 6% for asymptomatic women and 6% for symptomatic women with evidence of possible recent ZIKV infection. Zika virus-associated birth defects in infants with ZIKV infection: 10% in completed pregnancies with reported outcomes; 5% in infants with possible ZIKV-associated birth defects from women with confirmed or probably ZIKV infection) (5% among symptomatic and 4% among asymptomatic women). Among 1,508 pregnancies with lab-confirmed ZIKV (5% among symptomatic and 7% among asymptomatic woman). Adverse fetal outcomes: 7% in pregnant women with symptomatic ZIKV infection. Adverse outcomes: 3 of 4 ZIKV infected pregnant women.					
**Nithiyanantham et al. (2019) [32]**	Prevalence of joint abnormalities: 13.2% in infants of ZIKV-infected mothers	In infants of ZIKV-infected mothers: Ventriculomegaly / hydrocephaly: 21.8% (95% CI,15.2-28.4); Brain calcifications: 42.6% (95% CI, 30.8-54.4)	Prevalence of 3.9% in infants of ZIKV-infected mothers	Prevalence: 4.2% in infants of ZIKV-infected mothers		
**Masel et al. (2019) [33]**	No association of prior exposure to DENV and fetal imaging abnormalities					
**Barbosa et al. (2019) [34]**	Microcephaly or neurologic changes: 50.10% on 962 fetus or children studied				Altered OAE varied from 0% to 75%, while altered a-ABR varied from 0% to 29.9%. Among patients who underwent OAE assessments (n=244), 18,4% presented alterations while 25% of microcephaly cases dislpayed alterations. Among the 448 patients who reportedly underwent the first a-ABR test, 15.2% presented alterations. Among three studies that included 102 children with laboratory confirmation of congenital ZIKV infection, 18 (17.6%) had hearing alterations, five in the ABR and 13 in the HINE.	
**Minhas et al. (2017) [35]**						Cohort with 9 adults positive for ZIKV and no previous cardiac history. 8 of the cases had arrhythmicas and 6 presented heart failure. Of the 8 arrhythmias, 3 were acute atrial fibrillation (two paroxysmal, one persistent), 2 were non-sustained atrial tachycardia, and 2 were ventricular arrhythmias. 5 of the 6 heart failure patients had a low ejection fraction (EF), and one had preserved EF with pre-eclampsia and moderate to severe pericardial effusion.

Supplementary file 2 - Table 2. Health outcomes - Neurological

Authors	Neurological complications	Epilepsy	Sleep characteristics	GBS
**Krauer et al. (2017) [15]**				74-84% symptomatic ZIKV in GBS cases; ZIKV laboratory-confirmed in GBS cases investigated: 100%
**Paixao et al. (2016) [16]**	French Polynesia outbreak: Amongg patients that visited health care facilities with Zika-like symptoms, 2.3 per 1,000 had neurological complications			In Bahia, Brazil, GBS was diagnosed in 1 of every 1,000 reported ZIKV cases. French Polynesia outbreak: Among patients that visited health care facilities with Zika-like symptoms, 1.3/1,000 ZIKV infections had GBS. ZIKV symptomatic cases when confirmed Among 42 GBS cases, 36% required intensive care and 21% required mechanical ventilation; El salvador: Prevalence of 35% (84 GBS cases in 240 ZIKV infections)
**Simoes et al. (2016) [19]**				In the primary databases consulted, there is only one case report occurred in French Polynesia in which GBS was diagnosed in a patient infected with Zika virus.
**Padilla et al. (2016) [20]**				An analysis of 42 patients who developed GBS during the French Polynesia outbreak estimates the incidence of the disease to be 0.24 per 1000 Zika virus infections. 88% of these patients reported symptoms and 93% of patients showed evidence of recent disease with ZIKV confirmed by the presence of IgM antibodies. Of these patients, 38% required admission to an intensive care unit and 29% required mechanical ventilation.
**Marques et al. (2019) [21]**		Prevalence of epilepsy: 42.2-67% in children with congenital ZIKV. Infantile spasms: 72%, 21.6%. Generalized: 11.8%. Partial: 8.9%. Described as brief jerking spells of flexion and/or extension movements that lasted a few seconds : 21.57%. Focal motor seizures: 21%. Tonic seizures: 4%. Myoclonic seizures: 2%. Myoclonic seizures: 1%.	34.1% (30 in 88 congenital ZIKV-infected children) were defined as poor sleepres and 24% (21 in 88) slept less than 9 hours	

Authors	Neurological complications	Epilepsy	Sleep characteristics	GBS
**Counotte et al. (2018) [22]**				Prevalence ratio during the ZIKV transmission over pre-outbreak period: 2.0-9.8.
**Haby et al. (2018) [23]**				Prevalence of asymptomatic ZIKV infection in patients with GBS: 0.12
**Wahid et al. (2018) [25]**	A recent study presented neurological disorders in 12 of 16 patients co-infected with ZIKV, chikungunya virus, and dengue virus in Guayaquil, Ecuador. One patients experienced CNS vasculitis, three had GBS whereas, and six patients were diagnosed with meningitis or encephalitis.			About 43% of GBS patients were found to be positive for ZIKV. Another study confirmed ZIKV-linked GBS in 1 of 3 patients.
**Barbi et al. (2018) [27]**				Meta-analysis: 1513 GBS cases in 164,651 ZIKV-infected individuals (0.92%). Estimative the prevalence of GBS to be 1.23% (CI: 95% 1.17%-1.29%) of all ZIKV infection cases in adults. 16 in 38 GBS cases (42%) needed intensive care unit hospitalization (French Polynesia)
**Wachira et al. (2018) [29]**				OR: 59.7 (CI: 95% 10.4 - ∞); Other study: no statistical significance between ZIKV and GBS
**Pomar et al. (2019) [30]**		9-95.5% in congenital ZIKV infection		Prevalence of 1.23% (95% CI, 1.17%-1.29%) in general ZIKV infected-population)
**Wilder-Smith et al. (2018) [31]**				2.15% (2 cases in 93 ZIKV cases recorded in Geosentinel sites)
**Masel et al. (2019) [33]**	No association of prior exposure to DENV and clinical neurological assessment of fetus			No statistically significant difference in patients with GBS with or without prior DENV exposure. No statistical difference in prior DENV exposed patients with or without GBS after ZIKV infection.

Supplementary file 2 - Table 3. Health outcomes – Adverse outcomes

Authors	Death due ZIKV infection	Abortion due to ZIKA / fetal death / perinatal death / neonatal death	Intrauterine growth restrictions - Rate within mother-infant pairs	Abnormal amniotic fluid	Adverse birth outcomes
**Krauer et al. (2017) [15]**		Prevalence in all pregnancy outcomes: Miscarriage 2.5%; intrauterine death or stillbirth 1.1%; termination of pregnancy 5.4%; Neonatal death: 3.2%	28.57% of cases	Rate: 18% of infected pregnant women	
**Paixão et al. (2016) [16]**	In Brazil, 2 deaths of adults were attribuited to Zika and 7 are under investigation by the Ministry of Health; El Salvador (240 ZIKV cases, 2 deaths)				
**Chibueze et al. (2017) [17]**					
**Coelho et al. (2017) [18]**		Miscarriages and perinatal deaths: USA (22% - 2 deaths in 9 ZIKV infected pregnant women), Brazil (6.7% - 9 deaths in 135 ZIKV infected pregnant women), Puerto Rico (3% - 2 deaths in 67 ZIKV infected pregnant women), USA (10.6% - 47 deaths in 442 ZIKV infected pregnant women), French Guiana (4% - 20 deaths in 498 ZIKV infected pregnant women).			
**Simões et al. (2016) [19]**		In Brazil, 1.79% (91/5,079) of microcephaly reported cases, progressed to miscarriage or postpartum death. According to the classification, 64.8% (59/91) remained under investigation; 838% (8/91) were investigated and discarded, and 26.4% (24/91) were investigated and confirmed for microcephaly and/or changes in the CNS.			
**Padilla et al. (2016) [20]**		In 72 women with Zika-positive serology during pregnancy in Brazil, the fetal death rate was 4.8%; Zika infection during 9 pregnancies confirmed by CDC resulted in outcomes of 2 spontaneous abortions and 2 elective abortions.			
**Wahid et al. (2018) [25]**			One study with 88 pregnant women of which 72 were positive for ZIKV and ultrasonography was performed in 42: in utero growth restriction with or without microcephaly (5/42).		
**Pomar et al. (2019) [30]**		14% of proven infected fetuses/newborn	Prevalence of IUGR in CZS: 14%		
**Masel et al. (2019) [33]**		No association of prior exposure to DENV and fetal loss			Occured in 46.4% of those ZIKV infected participants
