# Supplementary material for: Health outcomes associated with Zika virus infection in humans: a systematic review of systematic reviews
Source: BMJ Open. 2019 Nov 3;9(11):e032275. doi: 10.1136/bmjopen-2019-032275 (PMC6858219; doi:10.1136/bmjopen-2019-032275)
Supplement: Supplementary data [file bmjopen-2019-032275supp003.pdf]

Supplementary file 3 - Table 1.1. Summary of AMSTAR 2 rating

AMSTAR 2	Krauer et al. [15]	Paixão et al. [16]	Chibueze et al. [17]	Coelho et al. [18]	Simões et al. [19]	Padilla et al. [20]	Marques et al. [21]
1	Yes	Yes	Yes	Yes	Yes	No	Yes
2	No	No	Partial yes	No	No	No	Yes
3	Yes	Yes	Yes	Yes	Yes	Yes	Yes
4	Partial yes	Yes	Partial yes	Partial yes	Partial yes	Yes	Partial yes
5	Yes	Yes	Yes	Yes	Yes	Yes	Yes
6	Yes	No	Yes	Yes	No	No	No
7	Yes	Yes	Yes	Yes	No	No	No
8	Partial yes	Partial yes	Yes	Partial yes	No	No	No
9	No	No	No	No	No	No	No
10	Yes	No	Yes	Yes	No	No	Yes
11	No	No	No	No	No	No	No
12	Yes	No MA conducted	No MA conducted	Yes	No MA conducted	No MA conducted	No MA conducted
13	No	No MA conducted	No MA conducted	No	No MA conducted	No MA conducted	No MA conducted
14	No	No	No	No	No	No	No
15	Yes	Yes	No	Yes	No	No	No
16	No	No MA conducted	No MA conducted	No	No MA conducted	No MA conducted	No MA conducted

*MA - Meta-analysis

Questions 2,4,7,9,12 and 14, highlighted, are those of critical domains.

1 - Did the research questions and inclusion criteria for the review include the components of PICO?

2 - Did the report of the review contain an explicit statement that the review methods were established prior to the conduct of the review and did the report justify any significant deviations from the protocol?

3 - Did the review authors explain their selection of the study designs for inclusion in the review?

4 - Did the review authors use a comprehensive literature search strategy?

5 - Did the review authors perform study selection in duplicate?

6 - Did the review authors perform data extraction in duplicate?

7 - Did the review authors provide a list of excluded studies and justify the exclusions?

8 - Did the review authors describe the included studies in adequate detail?

9 - Did the review authors use a satisfactory technique for assessing the risk of bias (RoB) in individual studies that were included in the review?

10 - Did the review authors report on the sources of funding for the studies included in the review?

11 - If meta-analysis was performed did the review authors use appropriate methods for statistical combination of results?

12 - If meta-analysis was performed, did the review authors assess the potential impact of RoB in individual studies on the results of the meta-analysis or other evidence synthesis?

13 - Did the review authors account for RoB in individual studies when interpreting/ discussing the results of the review?

14 - Did the review authors provide a satisfactory explanation for, and discussion of, any heterogeneity observed in the results of the review?

15 - If they performed quantitative synthesis did the review authors carry out an adequate investigation of publication bias (small study bias) and discuss its likely impact on the results of the review?

16 - Did the review authors report any potential sources of conflict of interest, including any funding they received for conducting the review?

Supplementary file 3 - Table 1.2. Summary of AMSTAR 2 rating

AMSTAR 2	Counotte et al. [22]	Haby et al. [23]	Sarwar et al. [24]	Wahid et al. [25]	Soriano-Arandes et al. [26]	Barbi et al. [27]	Santos et al. [28]
**1**	Yes	Yes	Yes	Yes	Yes	Yes	Yes
**2**	Yes	Partial yes	No	No	No	No	No
**3**	No	Yes	No	No	No	No	No
**4**	Partial yes	Partial yes	Partial yes	No	No	No	No
**5**	Yes	No	No	Yes	No	No	No
**6**	Yes	No	No	No	No	Yes	No
**7**	No	Yes	No	No	No	No	No
**8**	Yes	Yes	No	Yes	Yes	Yes	No
**9**	No	No MA conducted	No MA conducted	No MA conducted	No MA conducted	No MA conducted	No
**10**	No	Yes	No	No	No	Yes	No
**11**	No	No	No	No	Yes	No	No
**12**	No MA conducted	Yes	No MA conducted	No MA conducted	No MA conducted	No	No MA conducted
**13**	No MA conducted	No	No MA conducted	No MA conducted	No MA conducted	No	No MA conducted
**14**	No	Yes	No	No	No	No	Yes
**15**	No	Yes	No	No	No	No	No
**16**	No MA conducted	Yes	No MA conducted	No MA conducted	No MA conducted	No	No MA conducted

Questions 2,4,7,9,12 and 14, highlighted, are those of critical domains.

1 - Did the research questions and inclusion criteria for the review include the components of PICO?

2 - Did the report of the review contain an explicit statement that the review methods were established prior to the conduct of the review and did the report justify any significant deviations from the protocol?

3 - Did the review authors explain their selection of the study designs for inclusion in the review?

4 - Did the review authors use a comprehensive literature search strategy?

5 - Did the review authors perform study selection in duplicate?

6 - Did the review authors perform data extraction in duplicate?

7 - Did the review authors provide a list of excluded studies and justify the exclusions?

8 - Did the review authors describe the included studies in adequate detail?

9 - Did the review authors use a satisfactory technique for assessing the risk of bias (RoB) in individual studies that were included in the review?

10 - Did the review authors report on the sources of funding for the studies included in the review?

11 - If meta-analysis was performed did the review authors use appropriate methods for statistical combination of results?

12 - If meta-analysis was performed, did the review authors assess the potential impact of RoB in individual studies on the results of the meta-analysis or other evidence synthesis?

13 - Did the review authors account for RoB in individual studies when interpreting/ discussing the results of the review?

14 - Did the review authors provide a satisfactory explanation for, and discussion of, any heterogeneity observed in the results of the review?

15 - If they performed quantitative synthesis did the review authors carry out an adequate investigation of publication bias (small study bias) and discuss its likely impact on the results of the review?

16 - Did the review authors report any potential sources of conflict of interest, including any funding they received for conducting the review?

Supplementary file 3 - Table 1.3. Summary of AMSTAR 2 rating

AMSTAR 2	Wachira et al. [29]	Pomar et al. [30]	Wilder-Smith et al. [31]	Nithiyanantham et al. [32]	Masel et al. [33]	Barbosa et al. [34]	Minhas et al. [35]
**1**	Yes	Yes	Yes	Yes	Yes	Yes	Yes
**2**	Yes	No	No	No	No	Yes	No
**3**	Yes	No	Yes	Yes	Yes	Yes	Yes
**4**	Partial yes	No	Partial yes	Partial yes	Yes	Partial yes	Partial yes
**5**	Yes	Yes	No	Yes	Yes	Yes	Yes
**6**	Yes	Yes	No	No	Yes	Yes	Yes
**7**	No	No	No	No	No	No	No
**8**	Yes	No	Partial yes	Yes	Yes	Yes	Yes
**9**	No	No	No	No	No	Yes	No
**10**	Yes	No	No	No	No	No	No
**11**	No	No MA conducted	No MA conducted	No	No MA conducted	No MA conducted	No MA conducted
**12**	No MA conducted	No MA conducted	No MA conducted	No	No MA conducted	No MA conducted	No MA conducted
**13**	No MA conducted	No	No	No	No	No	No
**14**	Yes	No	Yes	Yes	No	Yes	No
**15**	Yes	No MA conducted	No MA conducted	Yes	No MA conducted	No MA conducted	No MA conducted
**16**	No MA conducted	Yes	Yes	Yes	Yes	Yes	Yes

Questions 2,4,7,9,12 and 14, highlighted, are those of critical domains.
1 - Did the research questions and inclusion criteria for the review include the components of PICO?
2 - Did the report of the review contain an explicit statement that the review methods were established prior to the conduct of the review and did the report justify any significant deviations from the protocol?
3 - Did the review authors explain their selection of the study designs for inclusion in the review?
4 - Did the review authors use a comprehensive literature search strategy?
5 - Did the review authors perform study selection in duplicate?
6 - Did the review authors perform data extraction in duplicate?
7 - Did the review authors provide a list of excluded studies and justify the exclusions?
8 - Did the review authors describe the included studies in adequate detail?
9 - Did the review authors use a satisfactory technique for assessing the risk of bias (RoB) in individual studies that were included in the review?
10 - Did the review authors report on the sources of funding for the studies included in the review?
11 - If meta-analysis was performed did the review authors use appropriate methods for statistical combination of results?
12 - If meta-analysis was performed, did the review authors assess the potential impact of RoB in individual studies on the results of the meta-analysis or other evidence synthesis?
13 - Did the review authors account for RoB in individual studies when interpreting/ discussing the results of the review?
14 - Did the review authors provide a satisfactory explanation for, and discussion of, any heterogeneity observed in the results of the review?
15 - If they performed quantitative synthesis did the review authors carry out an adequate investigation of publication bias (small study bias) and discuss its likely impact on the results of the review?
16 - Did the review authors report any potential sources of conflict of interest, including any funding they received for conducting the review?
