# Supplementary material for: Health outcomes associated with Zika virus infection in humans: a systematic review of systematic reviews
Source: BMJ Open. 2019 Nov 3;9(11):e032275. doi: 10.1136/bmjopen-2019-032275 (PMC6858219; doi:10.1136/bmjopen-2019-032275)
Supplement: Author's manuscript [file bmjopen-2019-032275.draft_revisions.pdf]

BMJ Open

BMJ Open is committed to open peer review. As part of this commitment we make the peer review history of every article we publish publicly available.

When an article is published we post the peer reviewers' comments and the authors' responses online. We also post the versions of the paper that were used during peer review. These are the versions that the peer review comments apply to.

The versions of the paper that follow are the versions that were submitted during the peer review process. They are not the versions of record or the final published versions. They should not be cited or distributed as the published version of this manuscript.

BMJ Open is an open access journal and the full, final, typeset and author-corrected version of record of the manuscript is available on our site with no access controls, subscription charges or pay-per-view fees (http://bmjopen.bmj.com).

If you have any questions on BMJ Open's open peer review process please email info.bmjopen@bmj.com

BMJ Open

Health outcomes associated with Zika virus infection in humans: a systematic review of systematic reviews

Journal:	*BMJ Open*
Manuscript ID	bmjopen-2019-032275
Article Type:	Research
Date Submitted by the Author:	12-Jun-2019

Complete List of Authors:	Ximenes, Raphael; University Health Network, Toronto Health Economics and Technology Assessment (THETA) Collaborative<br>Ramsay, Lauren; University Health Network, Toronto Health Economics and Technology Assessment (THETA) Collaborative, ; University of Toronto, Institute of Health Policy, Management and Evaluation<br>Miranda, Rafael; University Health Network, Toronto Health Economics and Technology Assessment (THETA) Collaborative<br>Morris, Shaun; Hospital fo sick children, Division of Infectious Diseases and Centre for Global Child Health; University of Toronto Faculty of Medicine, Department of Pediatrics<br>Murphy, Kellie; Mount Sinai Hospital<br>Sander, Beate; University Health Network, Toronto Health Economics and Technology Assessment (THETA) Collaborative; University of Toronto, Institute of Health Policy, Management and Evaluation

Keywords:	Zika Virus infection, Epidemiology < INFECTIOUS DISEASES, Congenital Zika Syndrome, NEUROLOGY, Guillain-Barré syndrome, Microcephaly

SCHOLARONE™
Manuscripts

Health outcomes associated with Zika virus infection in humans: a systematic review of systematic reviews

Raphael Ximenes[1], Lauren C. Ramsay[1,2], Rafael N. Miranda[1], Shaun K. Morris[3,4], Kellie E. Murphy[5], Beate Sander[1,2,6,7]

[1] Toronto Health Economics and Technology Assessment (THETA) Collaborative, University Health Network, Toronto, ON, Canada, raphael.ximenes@theta.utoronto.ca.

[2] Institute of Health Policy, Management and Evaluation, University of Toronto, Toronto, ON, Canada.

[3] Division of Infectious Diseases and Centre for Global Child Health, Hospital for Sick Children, Toronto, ON, Canada.

[4] Department of Pediatrics, Faculty of Medicine, University of Toronto, Toronto, ON, Canada.

[5] Mount Sinai Hospital, Toronto, ON, Canada.

[6] Public Health Ontario, Toronto, ON, Canada.

[7] Institute for Clinical Evaluative Sciences; and Public Health Ontario, Toronto, ON, Canada.

For peer review only

Abstract

Objective: With the emergence of Zika virus (ZIKV) disease in Central and South America in the mid-2010s and recognition of the teratogenic effects of congenital exposure to ZIKV, there has been a substantial increase in new research published on ZIKV. Our objective is to synthesize the literature on health outcomes associated with ZIKV infection in humans.

Methods: We conducted a systematic review (SR) of SRs following PRISMA (Preferred Reporting Items for Systematic Reviews and Meta-Analyses) guidelines. We searched MEDLINE, Embase, and Cochrane databases from inception to February 13, 2019, and included SRs that reported ZIKV associated health outcomes. Three independent reviewers selected eligible studies, extracted data and assessed the quality of included SRs using the A MeaSurement Tool to Assess Systematic Reviews 2 (AMSTAR 2) tool. Conflicts were resolved by consensus or consultation with a third reviewer.

Results: The search yielded 1,402 unique articles, of which 15 SRs met our inclusion criteria. The 15 SRs ranged from descriptive to quantitative data synthesis, including one meta-analysis. The most commonly reported ZIKV-associated manifestations and health outcomes were microcephaly, congenital abnormalities, brain abnormalities, neonatal death, and Guillain-Barré syndrome. The included reviews were highly heterogeneous. The overall quality of the SRs was critically low with all studies having more than one critical weakness.

Conclusion: The evolving nature of the literature on ZIKV-associated health outcomes, together with the critically low quality of existing SRs, demonstrate the need for high-quality SRs to guide patient care and inform policy decision making.

Limitations:

- Lack of SRs on ZIKV in the literature
- Lack of information about the risks of severe outcomes related to ZIKV infection or the presence of specific outcomes

Strengths:

- Broad search strategy
- Without restrictions by language or publication type
- To our knowledge, this is the first SR of SRs about health outcomes associated with ZIKV infection in humans

For peer review only

Introduction

Zika Virus (ZIKV) was first discovered in 1947 in rhesus monkeys in Uganda (1). It is an arbovirus in the flavivirus family and typically causes mild illness in humans characterized by fever and rash. There were reports of sporadic cases of ZIKV infection in humans over the years in Asia and Africa (2), with the first large documented outbreak taking place in Yap, a Micronesian island, in 2007 (3). Since then, there have been reported outbreaks in French Polynesia (in 2013-2014), and most recently in South and Central America and the Caribbean (4). With the emergence of ZIKV in Brazil, there were over 800,000 estimated cases of ZIKV infection reported by countries and territories in the Americas by January 2018 (5). By March 2017, according to the latest World Health Organization (WHO) global situation report on Zika, 84 countries, territories or subnational areas had evidence of vector-borne ZIKV transmission (6). According to the CDC, until May 2019, there were 89 areas with current or past transmission, but no current outbreak of ZIKV (7).

Our understanding of Zika-associated clinical outcomes has evolved over time. Before human pathogenesis was understood, cellular level damage was apparent in animal studies in the 1950s (8). The first study in humans to suggest an association between ZIKV and human disease was a case-control study during an outbreak in French Polynesia between 2013 and 2014, suggesting an association with Guillain-Barre Syndrome (GBS). (9). However, the link between ZIKV in pregnant women and microcephaly in infants was only evident in the 2016 outbreak in South America (10). With the spread of ZIKV to new regions of the world and the extent of the outbreak in South and Central American and Caribbean countries, a substantial body of new research has been published in recent years about Zika.

A bibliometric analysis of ZIKV research that indexed in Web of Science found a significant increase in the number of studies being published beginning in 2015 (n=38 publications) to 2017 (n=1,962 publications) (11). Summarizing the large body of literature on outcomes associated with ZIKV infection is timely and needed.

The purpose of this systematic review (SR) of systematic reviews was to synthesize the currently known health outcomes associated with ZIKV infection in humans.

Methods

Search strategy and selection criteria

We searched MEDLINE, Embase, and Cochrane databases from inception to February 13, 2019. Our search strategy across all databases included concepts related to “Zika” and “systematic review” (complete search strategy found in Supplementary File 1). Our search strategy was not restricted by language or publication type. Three reviewers (RX, first reviewer; LR and RM second reviewers) independently screened titles, abstracts, and relevant full text of identified articles.

The inclusion criteria were defined as SRs that reported health outcomes of ZIKV infection in humans, i.e. clinical presentation and sequelae of ZIKV infection in humans. We excluded studies that

For peer review only

only reported symptoms (e.g., rash, fever) of ZIKV infection, diagnostic techniques, mosquito control, therapeutic regimes, vaccine and trial but not outcomes (e.g., GBS, Congenital Zika Syndrome). We followed the Preferred Reporting Items for Systematic Reviews and Meta-Analyses (PRISMA) guidelines for reporting results (12).

The data extraction was performed in duplicate by the reviewers. The SR methods were established prior to the conduct of the SR and the protocol for the current SR was registered with PROSPERO (CRD42018091087) and there were no deviations from the protocol.

Risk of bias assessment

We used the A MeaSurement Tool to Assess systematic Reviews 2 (AMSTAR 2) tool to critically appraise the included SRs (13). AMSTAR 2 is not intended to generate an overall score, but rather to assist in the identification of high-quality SRs. Three reviewers (RX, first reviewer; LR and RM, second reviewers) independently evaluated the quality of each study based on weaknesses in critical domains as defined by the AMSTAR 2 tool. Studies were rated based on the overall confidence in the results of the SR and defined as either high (zero or one non-critical weakness), moderate (more than one non-critical weakness), low (one critical flaw with or without non-critical weaknesses) or critically low (more than one critical flaw with or without non-critical weaknesses) (14). Critical domains included protocol registration, adequacy of the literature search, justification for excluding studies, risk of bias from individual studies included in the SR, appropriateness of meta-analytical methods, consideration of risk of bias when interpreting results, and assessment of publication bias (14). Any disagreements between the two reviewers were resolved by consensus.

Data analysis

Three reviewers (RX, first reviewer; LR and RM, second reviewers) extracted the data using a structured electronic data extraction form, extracting study characteristics, and measures of effect for outcomes resulting from ZIKV infection. Included studies were summarized narratively, and health outcomes were reported where possible.

Results

We identified 1,402 unique articles from the database searches (Figure 1). After screening titles and abstracts, we selected 62 for full text screening. Of these, fifteen met our inclusion criteria (15–29). The main reasons for exclusion at the full text stage were articles not being SRs (but rather overviews or literature reviews) and studies only reported symptoms but not outcomes.

For peer review only

Study characteristics are summarized in Table 1. The included SRs were published between February 2016 and February 2019. The types of studies eligible for inclusion in the SRs varied across studies; three SR did not include any information on the included studies (21,24,28), all other SRs included observational studies (one limited to only cohort studies (18)), and the majority (73%; n=11) included case reports and case series. One SR considered evidence from modelling studies, animal experiments, and in vitro experiments (15). Another did not limit to reports of primary data and included SRs, narrative reviews, and news articles (20).

The majority of studies included in the SRs were conducted in Brazil, the United States (US), French Polynesia and Colombia.

Risk of bias assessment

Of the fifteen SRs included, there was high inter-rater reliability between the reviewers (91%). The overall quality of the SRs was critically low with all studies identified as having more than one critical weakness with or without non-critical weaknesses. For all studies, the majority of answers for the seven critical domains of AMSTAR 2 tool were no or partial yes (Figure 2). Main weaknesses identified were the lack of an explicit statement that SR methods were established prior to the conduct of the SR, a deficient bibliographic search strategy, and an unsatisfactory assessment of the risk of bias and its likely impact on the results of the SR.

Summary of included SRs and outcomes

Of the 15 included SRs, the most commonly reported outcome was microcephaly, reported in 12 SRs (15–26), 9 SRs reported on GBS (15,16,19,20,22,23,25,27,29), 6 SRs each reported on malformations or congenital abnormalities (18–20,22,26,29) and brain abnormalities (15,24–26,28), 5 SRs reported on ocular disorders (15,18,21,24,25), and 4 SRs on termination of pregnancy, fetal death and perinatal death (15,18–20). Three SRs or fewer reported on intrauterine growth restrictions (15,17,25), auditory disorder (15,26), cardiovascular damage (18,26), neurological complications (16,25), neonatal death (15), abnormal amniotic fluid (15), epilepsy (21), and death due Zika infection (16).

Seven SRs focused on pregnant women (17–20,24,26,28) and 5 SRs included the general population (15,16,22,23,29), while newborns, neonates, perinatal, early birth or infants were included in 5 five SRs (18,19,21,25,26). Adults were the included in two of the 15 SRs (25,27).

Health Outcomes

The Supplementary File 2 reports the health outcome data extracted from the fifteen SRs.

For peer review only

Clinical Presentation of ZIKV Infection

The most commonly reported symptom associated with ZIKV infection was rash; however, none of the included SRs reported the number of cases. Other common symptoms reported were fever (17–20,23,25,27), arthralgia (17,23,25,27), conjunctivitis (17,19,20,23) myalgia (20,23,27), dehydration (20), headache, fatigue (19,25), joint pain in the extremities (18–20), retro orbital and abdominal pain (19,23), diarrhea (17,19,25), vomiting (17,19), constipation, cough (19) and malaise (23,25).

For studies focused on pregnant women, rash was also described as 'a pruritic cutaneous rash with itching in the back and hands', 'generalized, descending macular' or 'generalized maculopapular', 'petechial' or 'generalized' (17). Other reported signs and symptoms of ZIKV infection in pregnant women included fever, chills, malaise, arthralgia, myalgia, myotonia, asthenia, jaundice, paraesthesia, hemiparesis, headache, conjunctivitis or conjunctival injections, lymphadenopathy, pain (eye, abdominal, lumbar, pelvis, body and joint), anaemia, edema in lower limbs, nausea, vomiting, dermal bleeding and 'respiratory findings' (17).

Coinfection

Coinfection was reported with dengue (16–18,25), chikungunya (16,17,25) and HIV (16,17); cytomegalovirus, toxoplasmosis, or other known teratogenic agents (16–18); hepatitis B virus (HBV), hepatitis C virus (HCV), cytomegalovirus (CMV), herpes simplex virus (HSV), Epstein-Barr virus (EBV), rubella, human T lymphotrophic virus (HTLV), parvovirus B19 and syphilis (17).

Clinical Outcomes Associated with ZIKV Infection During Pregnancy

The Supplemental Table shows that the reported outcomes associated with ZIKV infection during pregnancy ranging from malformations to perinatal death. The frequency of infant deaths (miscarriages and perinatal deaths) ranged from 3% to 22%, from two different studies (18). Miscarriage, intrauterine death or stillbirth and termination of pregnancy have also been described (15).

We classified as congenital abnormalities the outcomes reported as 'neural tube defects and early brain malformations eye anomalies, or consequence of CNS dysfunction without brain abnormalities or microcephaly' (18), 'abnormalities detected on ultrasound' (20) or 'malformations of the central nervous system' (19). Brain abnormalities were explicitly reported with data from 19 studies in which 96% (205 in 213 pregnant women) of fetuses were diagnosed after confirmation with imaging tests (15). One SR reported the prevalence of brain abnormalities (28%) including microcephaly in newborns whose mothers were infected with ZIKV in pregnancy (25). Further, three SRs classified the type of brain abnormalities or where the lesions were found (21,24,28) as intracranial calcification, reduction in the constitution of gyri of the severe cerebral cortex, abnormal hypodensity of the white matter, subcortical-cortical junction calcifications, basal ganglia calcification, basal ganglia calcification, ventriculomegaly / hydrocephaly and diffuse involvement of all the cerebral lobes.

Microcephaly was reported in 12 of 15 SRs. Chibueze et al. (2016) provided a trimester-specific modeling estimate risk for microcephaly. When the infection occurs in an indeterminate period of pregnancy, ZIKV associated microcephaly was described by Coelho et al. (2017). The authors performed a meta-analysis and found a prevalence of 2.3% (95% CI 1% - 5.3%) of microcephaly when considering all pregnancies. When considering only live births instead of all pregnancies, the prevalence of microcephaly was 2.7% (95% CI 1.2% - 6%) (18). Microcephaly cases per birth, live births and prevalence were described in four SRs, ranging from 0.03% to 14.3% (15,16,19,20). Microcephaly risk was reported in four SRs. The absolute risk varied between 0.95% (95% CI: 0.34 – 1.91%) to 30% (22,24–26). Death caused by microcephaly was estimated in a study reported by Coelho et al. (2017), reporting a rate of 8.3% (171 deaths among 2063 confirmed cases of microcephaly) (18). The prevalence of microcephaly in asymptomatic ZIKV infection was also reported as 0.36 (0.22 – 0.51) (23). Another SR reported that in a series of 13 infants with congenital ZIKV infection and microcephaly, more than half of the mothers did not report any symptoms of ZIKV prior to delivery (26).

The prevalence of congenital ZIKV syndrome-related outcomes is still unknown for many outcomes. In this SR of SRs we found the intrauterine growth restrictions rate reported varied from 28.57% (15) to 31.43%, from one observational study of 35 infants with microcephaly (17). Another study reported intrauterine growth restriction in 11.9% of fetuses with or without microcephaly (5 fetuses from 42 positives for ZIKV pregnant women) (25). The prevalence of ocular disorder was reported in five SRs ranging from 0.9% to 58.6% (18,25). Abnormal amniotic fluid was described only by Krauer et al. (2017). Auditory disorder was described by Krauer et al. (2018) (prevalence of 13% - 3 cases in 24 mother-infant pairs) and Soriano-Arandes et al. (2018) (prevalence of 7% - 5 cases in 70 children with laboratory diagnosis of ZIKV infection). While the prevalence of cardiovascular damage was reported by Coelho et al. (2017) (prevalence of 1%) and Soriano-Arandes et al. (2018) (prevalence of 13.6% - 14 cases in103 ZIKV cases).

Neurological Complications Associated with ZIKV Infection

Among adults, the proportion of neurological complications associated with ZIKV infection in Bahia (Brazil) was similar to that in French Polynesia. Among these neurological complications, GBS was diagnosed in 1 of every 1,000 reported Zika cases in Brazil and 1.3 per 1,000 in French Polynesia (16). During the French Polynesia outbreak in 2013, the incidence of GBS has been 0.24 per 1,000 ZIKV infections (20), and Simões et al. (2016) reported that the incidence of 1 to 4 cases per 100,000 inhabitants, after infectious processes by dengue virus and chikungunya (19).

Counotte et al. (2018) reported the increased incidence of GBS in seven different countries ranging from 2.0 (95% CI: 1.6-2.6) to 9.8 (95% CI: 7.6-12.5), while Barbi et al. (2018) conducted a meta-analysis of the prevalence of GBS in ZIKV infected cases. Their estimate for the prevalence of GBS in adults infected with ZIKV was 1.23% (CI: 95% 1.17%-1.29%). Krauer et al. (2017) reported the prevalence of symptomatic ZIKV in GBS cases. Padilla et al. (2016), Paixão et al. (2016) and Barbi et al. (2018), described the prevalence of admission to an intensive care unit and mechanical ventilation among the

For peer review only

GBS cases in French Polynesia. The interval between ZIKV and GBS symptoms was described by Krauer et al. (2017), Paixão et al. (2016), Padilla et al. (2016) and Counotte et al. (2018). The highest interval was reported by Paixão et al. (2016), where 88% of GBS cases reported a viral syndrome up to 23 days before the onset of the neurologic syndrome. No deaths due to GBS related with ZIKV infections were reported in this SR.

Epilepsy and sleep profiles were described only by Marques et al. (2019). The prevalence of epilepsy in congenital ZIKV infants ranged from 42% to 67%, and 34% of the ZIKV infected children were defined as poor sleepers (21).

Deaths Associated with ZIKV Infection

Deaths due to Zika infection are rare. According to the Brazilian Ministry of Health, between 440,000 and 1,300,000 cases of Zika occurred in Brazil in 2015 (30,31). Since the beginning of the outbreak 11 deaths among adults were confirmed in Brazil and an additional nine deaths were reported by the countries and territories in the Americas (5).

Discussion

Our SR of SRs identified 15 SRs that reported health outcomes associated with ZIKV infection. Rash, fever, conjunctivitis, myalgia, headache and joint pain were the most commonly reported symptoms associated with ZIKV infection. Microcephaly was the most commonly reported health outcome. Other outcomes reported were fetal death, neonatal death, congenital abnormalities including brain abnormalities, intrauterine growth restrictions, ocular disorders, and infant disorders including auditory disorders, cardiovascular damage, death due ZIKV infection, neurological complications, and epilepsy and finally adult outcomes including GBS.

Overall, we found high heterogeneity among the fifteen included SRs ranging from descriptive SRs, with few data on health outcomes associated with ZIKV infection, to more quantitative SRs, including three meta-analyses. Given this heterogeneity it was not possible to perform the meta-analysis or other quantitative synthesis, making it difficult to compare the results or draw conclusions based on the included SRs. Further, our quality appraisal found that all SRs were of critically low quality.

Our SR has some limitations. Since ZIKV is an emerging disease, one limitation is the lack of SRs on ZIKV in the literature. Given that the Brazilian outbreak prompted much of the recent research, the included SRs were conducted fairly early in the epidemic, which explains the lack of information about the risks of severe outcomes related to ZIKV infection or the presence of specific outcomes, caused by the inability to observe outcomes that are only evident or possible to detect after a longer time after birth.

For peer review only

Our study was strengthened by using a broad search strategy, without restrictions by language or publication type, reducing selection bias. To our knowledge, this is the first SR of SRs about health outcomes associated with ZIKV infection in humans.

As SRs of SRs aim to provide a summary of evidence from other SRs, although we were not able to perform a meta-analysis, our SR synthesizes findings from SRs on health outcomes associated with ZIKV virus infection in humans.

The evolving nature of the literature on ZIKV-associated health outcomes together with the critically low quality of existing SRs, confirm the need for high-quality SRs to better understand the burden of ZIKV, guide patient care and inform health policy.

Conclusion

Our SR demonstrates the need for future SRs on health outcomes associated with ZIKV infection as more research is published. As the ZIKV epidemic continues to evolve and the time since the emergence of the Brazilian outbreak increases we expect more primary observational studies on associated short- and long-term health outcomes to be published and synthesized in future SRs.

Acknowledgements

The authors would like to acknowledge Andrea Tricco and Joanna Bielecki for her guidance on this project.

Author Contributions

RX, LCR and BS conceptualized the research question. RX, LCR and RM conducted the database search, screened the articles, performed data extraction, and performed quality appraisal on the included studies. RX drafted the manuscript. All authors critically reviewed the manuscript.

Funding
This project was funded by the Canadian Institutes of Health Research - Team grant - FRN149784.

Conflicts
The authors have no conflicts of interest to declare.

For peer review only

Table 1. Summary of included systematic reviews

Author, Year	Aim	Number of studies included	Types of studies included in review	Jurisdictions of included studies (n studies)
Coelho, A.V.C., 2017	To summarize evidence and meta-analyze data to estimate prevalence of microcephaly in babies born to ZIKV infected pregnant women	8	Cohort studies	Brazil (1)<br>Colombia (1)<br>French Guiana (1)<br>Puerto Rico (1)<br>USA (4)
Krauer, F., 2017	To assess the relationship between ZIKV infection and congenital brain abnormalities and Guillain-Barré syndrome	106	Case reports, case series, case-control studies, cohort studies, cross-sectional studies, ecological study/outbreak reports, modelling studies, animal experiments, in vitro experiments, sequence analysis and phylogenetics	Not clearly reported. Most data are from Brazil; other jurisdictions included are Cabo Verde, Colombia, El Salvador, French Polynesia, Haiti, Honduras, Martinique, Panama, Puerto Rico, Suriname, Venezuela and Travelers returning from the Americas.
Padilla, C., 2016	To review clinical and basic science literature about ZIKV infection relevant for obstetric anesthesiologists	30	Systematic reviews, narrative reviews, case reports, epidemiologic studies, government reports, and news articles	Not clearly reported.
Simoes, R. 2016	To assess the effects of Zika virus infection on during pregnancy and postpartum periods	30	Case reports, case series, guidelines	Not clearly reported; most data are from Brazil.
Paixao, E.S., 2016	To summarize current knowledge on ZIKV including epidemiology, clinical presentation, and complications	41	Case reports, case series, surveillance reports, cross-sectional studies, epidemiological bulletins and alerts	Not clearly reported. Most data are from Brazil and French Polynesia.
Chibueze, E.C., 2017	To summarize guidance on pregnancy care in the context of ZIKV infection	18	Case reports, case series, observational studies	Brazil (11)<br>Colombia (1)<br>France (1)

For peer review only

Author, Year	Aim	Number of studies included	Types of studies included in review	Jurisdictions of included studies (n studies)
				Puerto Rico (1)<br>Slovenia (1)<br>USA (2)<br>Venezuela (1)
Santos, G., 2018	To analyze the association between Zika-virus and microcephaly during the gestational period	35	Not informed	
Wachira, V. K., 2018	To describe the factors associated with development of GBS, both infectious and non-infectious, through a SR.	34	The most common were case control, cohort, self-controlled case series	French Polynesia
Marques, V. de M., 2019	To map the neurological damage and outcomes related to congenital ZIKV infection	28	Not informed	Brazil (16)<br>USA (3)<br>Colombia (1)
Counotte, M. J., 2018	To summarize the evidence of the casual associations between ZIKV and CZS and GBS	101	Case report, case series, case-control study, cohort study, cross-sectional study, controlled trials, ecological study/outbreak report, modelling study, animal experiment, in vitro experiment, sequencing and phylogenetics, biochemical/protein structure studies	
Haby, M. M., 2018	To estimate the prevalence of asymptomatic Zika virus infection in the general population and in specific population groups from observational epidemiological studies	23	Cross-sectional seroprevalence studies, case series, case-control, cohort	USA (6)<br>Brazil (3)<br>French Polynesia (3)<br>French Guiana (3)<br>Puerto Rico (2)<br>Colombia (2)<br>Spain (2)<br>Micronesia (1)<br>Martinique (1)

For peer review only

**Author, Year**	**Aim**	**Number of studies included**	**Types of studies included in review**	**Jurisdictions of included studies (n studies)**
Sarwar, M. R., 2018	To report on the current literature regarding ZIKV and its hazardous effects on maternofetal health with a special emphasis on risk assessment, virus transmission, associated complications, and possible management	69	Not informed	
Wahid, B., 2018	To summarize the evidence of neurological complications in ZIKV-infected people	35	Case-studies, case-cohort studies, cross-sectional studies, organizational survey reports and case-control studies	Brazil (15)<br>French Polynesia (4)<br>Colombia (3)<br>USA, Slovenia, Suriname, Spain, Haiti, Martinique, Netherlands, Ecuador, Guyana (1)
Soriano-Arandes, A., 2018	To summarize the new evidence in aspects of epidemiology, virology, pathogenesis, associated risk factors during pregnancy, newborn phenotypic signs, neuroimaging, laboratory diagnosis, treatment and vaccines	106	Case series, cohort (prospective/retrospective), cross-sectional or case-control studies	
Barbi, L., 2018	To systematically review the literature and perform a meta-analysis to estimate the prevalence of GBS among ZIKV-infected individuals	3	Case series, epidemiological surveys, cross-sectional or cohort studies	French Polynesia (1)<br>Suriname and Dominican Republic (1)<br>7 South American, Central American and Caribbean countries (1)

For peer review only

References

1. World Health Organization. WHO | The History of Zika Virus. Who [Internet]. 2017 [cited 2018 Dec 10]; Available from: https://www.who.int/emergencies/zika-virus/timeline/en/

2. Posen HJ, Keystone JS, Gubbay JB, Morris SK. Epidemiology of Zika virus, 1947–2007. BMJ Glob Heal [Internet]. 2016 Aug [cited 2019 Feb 9];1(2):e000087. Available from: http://www.ncbi.nlm.nih.gov/pubmed/28588942

3. Duffy MR, Chen T-H, Hancock WT, Powers AM, Kool JL, Lanciotti RS, et al. Zika Virus Outbreak on Yap Island, Federated States of Micronesia. N Engl J Med [Internet]. 2009 Jun 11 [cited 2018 Dec 10];360(24):2536–43. Available from: http://www.nejm.org/doi/abs/10.1056/NEJMoa0805715

4. Kindhauser MK, Allen T, Frank V, Santhana RS, Dye C. Zika: the origin and spread of a mosquito-borne virus. Bull World Health Organ [Internet]. 2016 Sep 1;94(9):675–686C. Available from: http://www.who.int/entity/bulletin/volumes/94/9/16-171082.pdf

5. Pan American Health Organization / World Health Organization. Zika suspected and confirmed cases reported by countries and territories in the Americas Cumulative cases, 2015-2017. Updated as of 04 January 2018 [Internet]. Pan American Health Organization. Washington, D.C.; 2017 [cited 2019 Feb 9]. Available from: https://www.paho.org/hq/index.php?option=com_docman&view=download&category_slug=cumulative-cases-pdf-8865&alias=43296-zika-cumulative-cases-4-january-2018-296&Itemid=270&lang=en

6. World Health Organization. SITUATION REPORT ZIKA VIRUS MICROCEPHALY GUILLAIN-BARRÉ SYNDROME 10 MARCH 2017 DATA AS OF 9 MARCH 2017 [Internet]. [cited 2019 Feb 9]. Available from: https://apps.who.int/iris/bitstream/handle/10665/254714/zikasitrep10Mar17-eng.pdf?sequence=1

7. CDC. Zika Travel Information | Travelers' Health | CDC [Internet]. [cited 2019 May 28]. Available from: https://wwwnc.cdc.gov/travel/page/zika-travel-information

8. Dick GW. Zika virus (II). Pathogenicity and physical properties. Trans R Soc Trop Med Hyg [Internet]. 1952 Sep 1 [cited 2019 May 28];46(5):521–34. Available from: https://academic.oup.com/trstmh/article-lookup/doi/10.1016/0035-9203(52)90043-6

9. Cao-Lormeau VM, Blake A, Mons S, Lastere S, Roche C, Vanhomwegen J, et al. Guillain-Barre Syndrome outbreak associated with Zika virus infection in French Polynesia: a case-control study. Lancet. 2016;387:1531–9.

10. de Araújo TVB, Rodrigues LC, de Alencar Ximenes RA, de Barros Miranda-Filho D, Montarroyos UR, de Melo APL, et al. Association between Zika virus infection and microcephaly in Brazil,

For peer review only

January to May, 2016: preliminary report of a case-control study. Lancet Infect Dis [Internet]. 2016 Dec 1 [cited 2019 Jan 28];16(12):1356–63. Available from: http://www.ncbi.nlm.nih.gov/pubmed/27641777

11. Saima Nasir JA. A Bibliometric Analysis of Research on Zika Virus Indexed in Web of Science. Adv Life Sci [Internet]. 2018 [cited 2018 Dec 4];5(3):88–95. Available from: http://www.als-journal.com/532-18/

12. Moher D, Shamseer L, Clarke M, Ghersi D, Liberatî A, Petticrew M, et al. Preferred reporting items for systematic review and meta-analysis protocols (PRISMA-P) 2015 statement [Internet]. 2015. Available from: http://www.crd.york.ac.uk/prospero

13. AMSTAR - Assessing the Methodological Quality of Systematic Reviews [Internet]. [cited 2018 Dec 4]. Available from: https://amstar.ca/Amstar-2.php

14. Shea BJ, Reeves BC, Wells G, Thuku M, Hamel C, Moran J, et al. AMSTAR 2: a critical appraisal tool for systematic reviews that include randomised or non-randomised studies of healthcare interventions, or both. BMJ [Internet]. 2017 Sep 21 [cited 2018 Dec 4];j4008. Available from: http://www.bmj.com/lookup/doi/10.1136/bmj.j4008

15. Krauer F, Riesen M, Reveiz L, Oladapo OT, Martínez-Vega R, Porgo T V., et al. Zika Virus Infection as a Cause of Congenital Brain Abnormalities and Guillain–Barré Syndrome: Systematic Review. von Seidlein L, editor. PLOS Med [Internet]. 2017 Jan 3;14(1):e1002203. Available from: https://dx.plos.org/10.1371/journal.pmed.1002203

16. Paixão ES, Barreto F, Teixeira M da G, Costa M da CN, Rodrigues LC. History, Epidemiology, and Clinical Manifestations of Zika: A Systematic Review. Am J Public Health [Internet]. 2016 Apr 9;106(4):606–12. Available from: http://ajph.aphapublications.org/doi/10.2105/AJPH.2016.303112

17. Chibueze EC, Tirado V, Lopes K da S, Balogun OO, Takemoto Y, Swa T, et al. Zika virus infection in pregnancy: a systematic review of disease course and complications. Reprod Health [Internet]. 2017 Dec 28;14(1):28. Available from: http://reproductive-health-journal.biomedcentral.com/articles/10.1186/s12978-017-0285-6

18. Coelho A, Crovella S, Coelho AVC, Crovella S. Microcephaly Prevalence in Infants Born to Zika Virus-Infected Women: A Systematic Review and Meta-Analysis. Int J Mol Sci [Internet]. 2017 Aug 5;18(8):1714. Available from: http://www.mdpi.com/1422-0067/18/8/1714

19. Simões R, Buzzini R, Bernardo W, Cardoso F, Salomão A, Cerri G, et al. Zika virus infection and pregnancy. Rev Assoc Med Bras [Internet]. 2016 Apr;62(2):108–15. Available from: http://www.scielo.br/scielo.php?script=sci_arttext&pid=S0104-42302016000200108&lng=en&tlng=en

20. Padilla C, Pan A, Geller A, Zakowski MI. Zika virus: review and obstetric anesthetic clinical

For peer review only

considerations. J Clin Anesth [Internet]. 2016 Dec 1;35:136–44. Available from: https://www.sciencedirect.com/science/article/pii/S0952818016304299

21. Marques V de M, Santos CS, Santiago IG, Marques SM, Nunes Brasil M das G, Lima TT, et al. Neurological Complications of Congenital Zika Virus Infection. Pediatr Neurol [Internet]. 2019 Feb;91:3–10. Available from: http://www.ncbi.nlm.nih.gov/pubmed/30591235

22. Counotte MJ, Egli-Gany D, Riesen M, Abraha M, Porgo TV, Wang J, et al. Zika virus infection as a cause of congenital brain abnormalities and Guillain-Barré syndrome: From systematic review to living systematic review. PLoS Med [Internet]. 2018 Feb 15;7:196. Available from: http://www.ncbi.nlm.nih.gov/pubmed/30631437

23. Haby MM, Pinart M, Elias V, Reveiz L. Prevalence of asymptomatic Zika virus infection: a systematic review. Bull World Health Organ [Internet]. 2018 Jun 1;96(6):402–413D. Available from: http://www.ncbi.nlm.nih.gov/pubmed/29904223

24. Rehan Sarwar M, Saqib A, Iftikhar S. Zika Virus Infection during Pregnancy; Maternofetal Risk Assessment, Transmission, Complications, and Management: A Review of the Literature. Arch Clin Infect Dis [Internet]. 2018 Jun 24;13(3). Available from: http://archcid.com/en/articles/12848.html

25. Wahid B, Ali A, Waqar M, Idrees M. An updated systematic review of Zika virus-linked complications. Asian Pac J Trop Med [Internet]. 2018;11(1):1. Available from: http://www.apjtm.org/text.asp?2018/11/1/1/223527

26. Soriano-Arandes A, Rivero-Calle I, Nastouli E, Espiau M, Frick M, Alarcon A, et al. What we know and what we don't know about perinatal Zika virus infection: a systematic review. Expert Rev Anti Infect Ther [Internet]. 2018 Mar 4;16(3):243–54. Available from: http://www.ncbi.nlm.nih.gov/pubmed/29415586

27. Barbi L, Coelho AVC, Alencar LCA de, Crovella S. Prevalence of Guillain-Barré syndrome among Zika virus infected cases: a systematic review and meta-analysis. Brazilian J Infect Dis [Internet]. 2018 Mar;22(2):137–41. Available from: http://www.ncbi.nlm.nih.gov/pubmed/29545017

28. Santos GRB dos, Aragão FBA, Lobão WJ de M, Lima FR, Andrade LMRL de, Furtado QR, et al. Relationship between microcephaly and Zika virus during pregnancy: a review. Rev Assoc Med Bras [Internet]. 2018 Jul;64(7):635–42. Available from: http://www.ncbi.nlm.nih.gov/pubmed/30365666

29. Wachira VK, Peixoto HM, Fernandes De Oliveira MR. Systematic review of factors associated with the development of Guillain-Barr e syndrome 2007-2017: what has changed? 2018; Available from: https://v2dis-prod.evidencepartners.com/Generic/getAttachment2.php?id=44

30. Anthony Boadle LP. Exclusive: Brazil says Zika virus outbreak worse than believed | Reuters [Internet]. Reuters. 2016 [cited 2018 Dec 4]. Available from: https://www.reuters.com/article/us-

For peer review only

health-zika-brazil-exclusive-idUSKCN0VA331

31. World Health Organization. ZIKA SITUATION REPORT - ZIKA AND POTENTIAL COMPLICATIONS 12 FEBRUARY 2016 [Internet]. 2016 [cited 2018 Dec 4]. Available from: https://www.who.int/emergencies/zika-virus/situation-report/who-zika-situation-report-12-02-2016.pdf

For peer review only

Search Strategy

Database: Embase Classic+Embase <1947 to 2018 February 27>

Search Strategy:

----

1 zika fever/ or zika virus/ or zika virus vaccine/ or zika.mp. (5013)

2 "systematic review"/ or "review"/ (2367967)

3 1 and 2 (569)

****

Database: Ovid MEDLINE(R) <1946 to February Week 3 2018>

Search Strategy:

----

1 exp ZIKA VIRUS INFECTION/ or exp ZIKA VIRUS/ or zika.mp. (2287)

2 "review"/ (2215441)

3 1 and 2 (326)

****

Database: Cochrane

Search Strategy:

1 ZIKA and review (2)

Update – 13/02/2019

Database(s): **Ovid MEDLINE(R)** 1946 to January Week 5 2019

Search Strategy:

----

1 exp ZIKA VIRUS INFECTION/ or exp ZIKA VIRUS/ or zika.mp. (3846)

2 "review"/ (2316361)

3 1 and 2 (576)

4 limit 3 to yr=”2018-Current”

****

Database(s): **Ovid MEDLINE(R)** 1946 to January Week 5 2019

Search Strategy:

----

1 exp ZIKA VIRUS INFECTION/ or exp ZIKA VIRUS/ or zika.mp. (3846)

2 "review"/ (2316361)

3 1 and 2 (576)

4 limit 3 to yr=”2018-Current”

For peer review only

****

Database: Cochrane

Search Strategy:

1 ZIKA and review (0)

For peer review only

Authors			Coelho, A.V.C, 2017	Krauer, F., 2017	Padilla, C., 2016	Simoes, R. 2016	Paixao, E.S., 2016	Chibueze, E.C., 2017	Santos, G R B, 2018	Wachira, V K, 2018	Marques, V D M, 2019	Counotte, M J, 2018	Haby, M. M., 2018	Sarwar, M R, 2018	Wahid, B, 2018	Soriano-Arandes, A, 2018	Barbi, L, 2018
Number of studies included			8	106	30	30	41	18	35	34	28	101	23	69	35	106	3
Abortion due to ZIKA / fetal death / perinatal death			3%, 22%	Miscarriage 2.5% (7 in 278); intrauterine death or stillbirth 1.1% (3 in 278); termination of pregnancy 5.4% (15 in 278)	Fetal death prevalence: 1.39%	1.79%, where 26.4% were investigated and confirmed for microcephaly of changes in the CNS											
Neonatal death				3.2% - from 9 studies, 9 deaths out of 278 mother-infant pairs													
Time point of presumed exposure - symptoms	1st trimester			69% - 81 out of 117 pairs													
	2nd trimester			24% - 28 out of 117 pairs													
	3rd trimester			7% - 8 out of 117 pairs													
Malformations / Congenital abnormalities			Prevalence 2% to 7%		29% of 72 women Zika-positive during pregnancy	10 to 20 cases in 100,000 live births; 8.87% (41 confirmed as associated with Zika infection out of 462 confirmed changes in CNS)						One study: 58 out of 117 women with PCR confirmed ZIKV had adverse congenital outcomes compared to 7 out of 58 women negative ZIKV PCR results. RR=7.96 (95% CI 2.59-24.43). Another study: 27 out of 301 ZIKV positive women had adverse congenital outcomes versus 17 out of 399 in the unexposed group (RR 2.11 (95% CI 1.17-3.79). Another Study: Birth defects were reported in 16 out of 271 (6% 95% CI:4-9%) pregnant asymptomatic women and 10 out of 167 (6% 95% CI: 3-11%) symptomatic pregnant women(no significant difference between the groups).			One study with 88 pregnant women of which 72 were positive for ZIKV and ultrasonography was performed in 42: Fetal abnormalities (12/42) - ventricular calcifications or other central nervous system abnormal amniotic fluid volume or cerebral or umbilical artery flow (7/42) (CNS) lesions (7/42). Another study: 80 of the 185 infants, ZIKV-linked microcephaly in 10 neonates, 5 had additional birth defects such as hydranencephaly, holoprosencephaly, clubfeet, and craniosynostosis and other 3 had cataracts, holoprosencephaly, and ventral pons hypoplasia	Birth defects were reported with the same frequency (6%) for asymptomatic and symptomatic pregnant women. From 1 study: Fetal adverse outcomes were noted regardless of the trimester during which the women were infected with ZIKV, although they were more frequent in the first term of pregnancy (55%) versus the third trimester (29%). Another study: features consistent with fetal immobility, ranging from dimples (30.1%), distal hand/finger contractures (20.5%), and feet malposition (15.7%), to generalized arthrogryposis (9.6%), were present in infants. One study: out of 442 completed pregnancies in women with recent ZIKV infection, birth defects were identified in 26 (6%; CI:95% 4%-8%) fetuses or infants, but some of them had brain abnormalities without microcephaly.	
Brain abnormalities	Prevalence			205 in 213 (96%) of infected mother/infant pairs											Case-cohort study reported 28% brain abnormalities including microcephaly in newborns whose mothers had infected with ZIKV	A study of 48 infants with probable CZS and followed up to the age of 1–8 months: most of them had microcephaly (86.7%) and craniofacial disproportion (95.8%).	
	Intracranial calcification								23 of 23 childen								
	Frontal lobe								69% - 78%								
	Parietal lobe								83% - 87%								
	Corticomedullary junction								53% - 86%								
	Thalamus								39% - 43%								
	Punctate calcificaiton								72% - 100%								
	Distributed in the band format								56% - 75%								
	Reduction in the constitution of gyri of the severe cerebral cortex								0.78								
	Cerebellar hypoplasia								0.74								
	Involving only one cerebellar hemisphere								3 children (13%)								
	Brainstem globally hypoplastic								2 of them (8.7%)								
	Abnormal hypodensity of the white matter								1								
	Diffuse involvement of all the cerebral lobes								0.87								
	Pachygyria													1 case in 7 neonates (postmortem examination)			
	Arthrogryposis													1 in 7 neonates			
	Morphologic microcephalus changes													1 in 7 neonates			
	Head circumference still within range													7 in 7 neonates			
	Subcortical-cortical junction calcifications										92.9% (104 reported cases in 112 newborns - ranging from 25% to 100% in 7 studies)						
	Ventriculomegaly / hydrocephaly										63.10% 94% (16 of 17 patients)			7 in 7 neonates			
	Basal ganglia calcification								57% - 65%		57.10%						
	Cerebellar abnormalities										46.20% 82% (14 of 17 patients)			2 in 7 neonates			
	Corpus callosum abnormalities										47.90% 94.00%						
	Periventricular calcifications										29.50%						
Microcephaly	Undetermined trimester infection		0.3% - 4 cases in 1484 live-birth pregnancies; 14.3% - 1 case in 7 live-birth pregnancies; Prevalence (cases/all pregnancies): 2.3% (1% - 5.3%) Prevalence (cases/live births): 2.7% (1.2% - 6%)	91% (244 cases in 267 mother-infant pairs); 2.8 cases per 10,000 live births Prevalence ratio = 4.7 (1.9 - 13.3)	20 cases per 10,000 births; 1 case in 9 pregnant infected women	10 per 10,000 births	Rate per 100,000 live births: 121.7 (0.12%);				39.7% (1561 of 3931 cases of congenital Zika infection), reached almost 100% when the infection occurred during the first trimester and decreased when the infection occurred in the second or third trimester.	Risk ratio between ZIKV unexposed and exposed: 4.4 (95% CI: 0.2-80.8) in a cohort in Brazil and 6.6 (95% CI: 0.8-56.4) in a cohort in French Guiana; Prospective case-control study in Brazil: women with laboratory-confirmed ZIKV had 55.5 (85% CI: 8.6 - infinity) times the odds of having a baby with microcephaly compared with women without evidence of ZIKV infection. Retrospective case-control study in Hawaii found odds ratio of 11.0 (95% CI: 0.8-147.9) and ZIKV IgM antibody positive mothers were also more likely to deliver babies with microcephaly than mothers who were negative (OR = 6.8, 95% CI = 0.2-195.1).			From 1 study: absolute risk of microcephaly varied between 0% to 30%. Relative risk 100–1 000 (assuming 10% exposure) or 20–200 (assuming 50% exposure) compared to background risk of microcephaly that was around 2 per 10 000 live births. Another study: 1 501 liveborn infants reviewed of whom 602 were suspected ZIKV cases - among 319 definite or probable cases 161 had microcephaly and rash whereas remaining cases had one of two symptoms.		
	Number of cases per 10,000 ZIKV infected pregnant women	1st trimester						95 (34 - 191)						From 1 study: risk of microcephaly was found to be 1%	From 2 studies: Higher risk of microcephaly in pregnant women infected during first trimester. Eight cases of microcephaly were identified and the estimated risk of microcephaly was 0.95% (95 cases per 10 000 women infected in the first trimester)	From one study: Risk: 0.95% (95% CI, 0.34 to 1.91%), on the basis of eight microcephaly cases identified retrospectively in a population of approximately 270,000 people with an estimated rate of ZIKV infection of 66%. ; Rio de Janeiro: Prevalence of 55%	
		2nd trimester						84 (12 - 196)									
		3rd trimester						0 - (0 - 251)								Prevalence: 29% (Rio de Janeiro)	
	Prevalence of asymptomatic ZIKV infection												0.36 (0.22 - 0.51)			In a series of 13 infants with congenital ZIKV infection and progressive microcephaly, more than half of the mothers did not report any symptoms prior to delivery.	
	Death due microcephaly		8.3% (171 in 2063 microcephaly cases) would be 5.7% in case of new confirmed cases are included				1.3% (46 deaths in 3530 suspected microcephaly cases)										
Intrauterine growth restrictions - Rate within mother-infant pairs				10 cases in 35 pairs (28.57%)				31.43% - 11 cases in 35 pairs (35 microcephaly cases)							One study with 88 pregnant women of which 72 were positive for ZIKV and ultrasonography was performed in 42: in utero growth restriction with or without microcephaly (5/42).		
Ocular disorders	Prevalence		0.9% to 1%	42% (49 cases in 116 mother-infant pairs)							108 of 244 infants (44.3%). Bilateral findings were observed in 83 of those with ocular lesions (76.8%). Among 10 studies, ocular findings were present in 20% patients with microcephaly, 33% patients with ventriculomegaly, and 43% patients with calcification.				From 1 study with 40 infants with microcephaly: ophthalmoscopic alterations as well (22/40). Cross-sectional study evaluated the ocular findings with microcephaly associated with ZIKV: ocular abnormalities (17/29). In a case-control study ocular findings were confirmed in 34.5% of ZIKV linked microcephalic infants.		
	Macular lesions										108 eyes with lesion in 108 infants with ocular lesions from 244 infants with congenital ZIKV infection						
	Optical nerve abnormalities										60 eyes with lesion in 108 infants with ocular lesions from 244 infants with congenital ZIKV infection						
	Chorioretinal artrophy/scaring										23 eyes with lesion in 108 infants with ocular lesions from 244 infants with congenital ZIKV infection						
	Focal pigment mottling of retina										15 eyes with lesion in 108 infants with ocular lesions from 244 infants with congenital ZIKV infection						
	Microphthalmia										8 eyes with lesion in 108 infants with ocular lesions from 244 infants with congenital ZIKV infection						
	Glaucoma										5 eyes with lesion in 108 infants with ocular lesions from 244 infants with congenital ZIKV infection						
	Cataract										5 eyes with lesion in 108 infants with ocular lesions from 244 infants with congenital ZIKV infection						
	Iris coloboma										5 eyes with lesion in 108 infants with ocular lesions from 244 infants with congenital ZIKV infection						
	Subluxation										3 eyes with lesion in 108 infants with ocular lesions from 244 infants with congenital ZIKV infection						
	Retinal impairment													28% of 32 ZIKV infected infants			
	Impaired optic nerve													17% of 32 ZIKV infected infants			
	Optic nerve hypoplasia													8% of 32 ZIKV infected infants			
Auditory disorder - Rate				13% (3 cases in 24 mother-infant pairs)												In a study of 70 children with microcephaly and laboratory diagnosys of congenital ZIKV infection, 5 (7%) had sensorineural hearing loss.	
Abnormal amniotic fluid - Rate				18% (6 cases in 33 mother-infant pairs)													

For peer review only

Cardiovascular damage		1%														One study: congenital heart disease was described in 14 of a series of 103 cases (13.6%) in children with CZS.	
Death due ZIKV infection - Prevalence						1.5x10-6 to 2x10-5 ; El Salvador (240 cases, 46 GBS cases and 2 deaths)											
Neurological complications - Rate						2.3 per 1,000									A recent study presented neurological disorders in 12 of 16 patients co-infected with ZIKV, chikungunya virus, and dengue virus in Guayaquil, Ecuador. One patients experienced CNS vasculitis, three had GBS whereas, and six patients were diagnosed with meningitis or encephalitis.		
Epilepsy	Prevalence									67% - 95 in 141 congenital ZIKV cases	42.2% (43 in 102 children with congenital ZIKV)						
	Infantile spasms									72%	21.6%						
	Generalized										11.8%						
	Partial										8.9%						
	Described as brief jerking spells of flexion and/or extension										21.6%						
	Focal motor seizures									21%							
	Tonic seizures									4%							
	Tonic-clonic seizures									2%							
	Myoclonic seizures									1%							
Sleep characteristics										34.1% (30 in 88 congenital ZIKV-infected children) were defined as poor sleepres and 24% (21 in 88) slept less than 9 hours							
GBS Rate				0.24/1,000 ZIKV infections	1 to 4 cases per 100,000 inhabitants	1 to 1.3/1,000 ZIKV infections			OR: 59.7 (CI: 95% 10.4 - ∞); Other study: no statistical significance between ZIKV and GBS			One study: compares the reported pre-ZIKV GBS incidence with the incidence during the ZIKV transmission period in seven different countries, rate-ratios are significantly higher for all countries, ranging from 2.0 (95% CI: 1.6-2.6) to 9.8 (95% CI: 7.6-12.5) increase in incidence. Another study: increase from an average of 0.67 GBS cases per month to 5.4 cases per month. Surveillance data from Colombia: increase of GBS during the ZIKV outbreak from 20 cases per month to 90 cases per month.			About 43% of GBS patients were found to be positive for ZIKV. Another study confirmed ZIKV-linked GBS in 1 of 3 patients.		Meta-analysis: 1513 GBS cases in 164,651 ZIKV-infected individuals (0.92%). Estimative the prevalence of GBS to be 1.23% (CI: 95% 1.17%-1.29%) of all ZIKV infection cases in adults.
ZIKV symptomatic cases when confirmed GBS - Prevalence			74% - 84 symptomatic ZIKV in 113 GBS cases	88%		1 to 1.3 per 1,000											
ZIKV asymptomatic cases when confirmed GBS - Prevalence													0.12 (0.00 - 0.32)				
Rate of GBS cases in ZIKV positive in any test (serology/RT-PCR)			100%														
Interval between ZIKV and GBS symptoms - Days			Median 10 (range 3 - 12); Median 6 (QR 4-10); 7-15	6	7	Up to 23						5 to 10					
Sequelae caused by GBS - prevalence (Intensive care; mechanical ventilation)				38% - intensive care unit 29% - required mechanical ventilation		36% - intensive care 21% - mechanical ventilation											16 in 38 GBS cases (42%) needed intensive care unit hospitalization (French Polynesia)

For peer review only

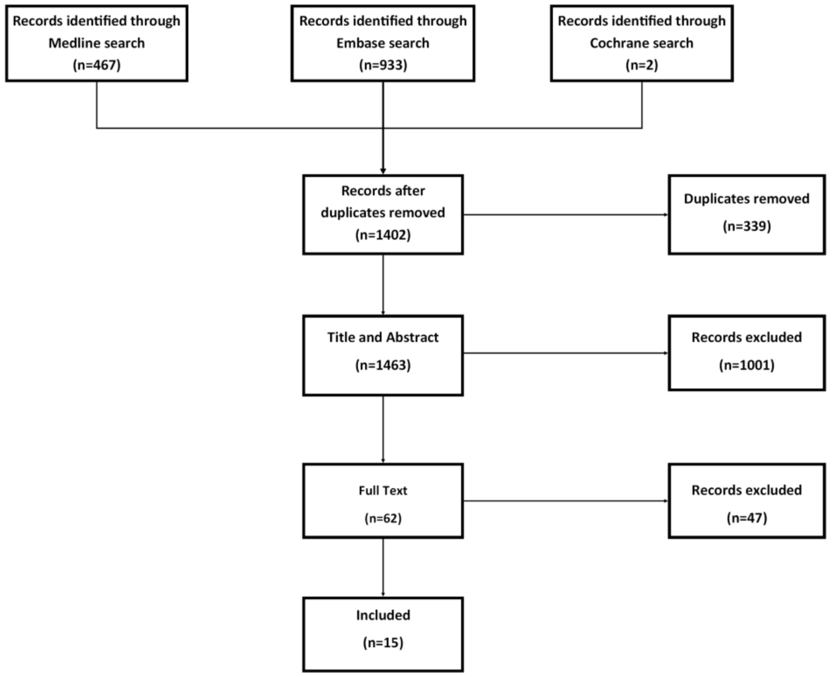

Figure 1. PRISMA flow diagram of search results and study selection

279x215mm (300 x 300 DPI)

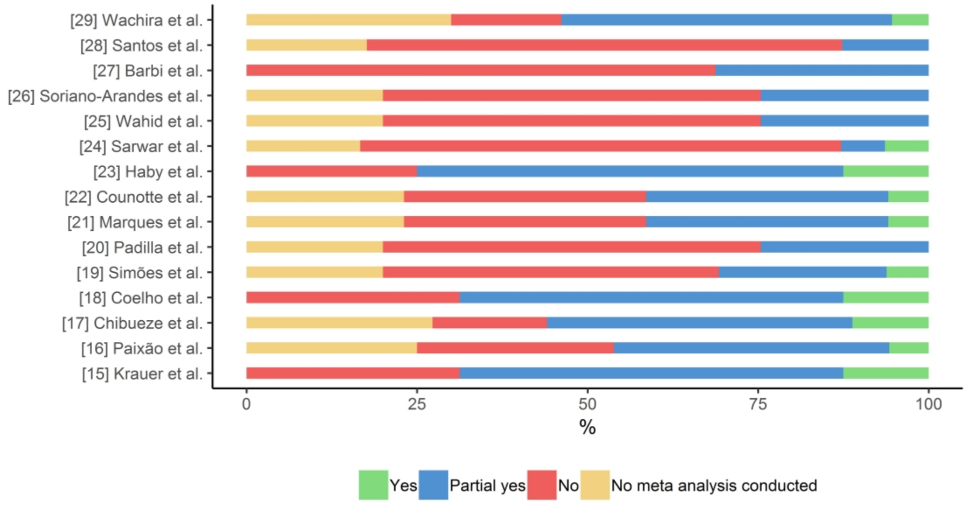

Figure 2. Individual study results of critical appraisal using AMSTAR 2

189x99mm (600 x 600 DPI)

PRISMA 2009 Checklist

Section/topic	#	Checklist item	Reported on page #
**TITLE**			
Title	1	Identify the report as a systematic review, meta-analysis, or both.	1
**ABSTRACT**			
Structured summary	2	Provide a structured summary including, as applicable: background; objectives; data sources; study eligibility criteria, participants, and interventions; study appraisal and synthesis methods; results; limitations; conclusions and implications of key findings; systematic review registration number.	2
**INTRODUCTION**			
Rationale	3	Describe the rationale for the review in the context of what is already known.	3
Objectives	4	Provide an explicit statement of questions being addressed with reference to participants, interventions, comparisons, outcomes, and study design (PICOS).	3
**METHODS**			
Protocol and registration	5	Indicate if a review protocol exists, if and where it can be accessed (e.g., Web address), and, if available, provide registration information including registration number.	3,4
Eligibility criteria	6	Specify study characteristics (e.g., PICOS, length of follow-up) and report characteristics (e.g., years considered, language, publication status) used as criteria for eligibility, giving rationale.	3,4
Information sources	7	Describe all information sources (e.g., databases with dates of coverage, contact with study authors to identify additional studies) in the search and date last searched.	3,4
Search	8	Present full electronic search strategy for at least one database, including any limits used, such that it could be repeated.	3
Study selection	9	State the process for selecting studies (i.e., screening, eligibility, included in systematic review, and, if applicable, included in the meta-analysis).	3,4
Data collection process	10	Describe method of data extraction from reports (e.g., piloted forms, independently, in duplicate) and any processes for obtaining and confirming data from investigators.	3,4
Data items	11	List and define all variables for which data were sought (e.g., PICOS, funding sources) and any assumptions and simplifications made.	
Risk of bias in individual studies	12	Describe methods used for assessing risk of bias of individual studies (including specification of whether this was done at the study or outcome level), and how this information is to be used in any data synthesis.	4
Summary measures	13	State the principal summary measures (e.g., risk ratio, difference in means).	5-6
Synthesis of results	14	Describe the methods of handling data and combining results of studies, if done, including measures of consistency (e.g., I^2) for each meta-analysis.	

For peer review only - http://bmjopen.bmj.com/site/about/guidelines.xhtml

PRISMA 2009 Checklist

Section/topic	#	Checklist item	Reported on page #
Risk of bias across studies	15	Specify any assessment of risk of bias that may affect the cumulative evidence (e.g., publication bias, selective reporting within studies).	
Additional analyses	16	Describe methods of additional analyses (e.g., sensitivity or subgroup analyses, meta-regression), if done, indicating which were pre-specified.	
**RESULTS**			
Study selection	17	Give numbers of studies screened, assessed for eligibility, and included in the review, with reasons for exclusions at each stage, ideally with a flow diagram.	4
Study characteristics	18	For each study, present characteristics for which data were extracted (e.g., study size, PICOS, follow-up period) and provide the citations.	5
Risk of bias within studies	19	Present data on risk of bias of each study and, if available, any outcome level assessment (see item 12).	5
Results of individual studies	20	For all outcomes considered (benefits or harms), present, for each study: (a) simple summary data for each intervention group (b) effect estimates and confidence intervals, ideally with a forest plot.	
Synthesis of results	21	Present results of each meta-analysis done, including confidence intervals and measures of consistency.	
Risk of bias across studies	22	Present results of any assessment of risk of bias across studies (see Item 15).	
Additional analysis	23	Give results of additional analyses, if done (e.g., sensitivity or subgroup analyses, meta-regression [see Item 16]).	
**DISCUSSION**			
Summary of evidence	24	Summarize the main findings including the strength of evidence for each main outcome; consider their relevance to key groups (e.g., healthcare providers, users, and policy makers).	6-8
Limitations	25	Discuss limitations at study and outcome level (e.g., risk of bias), and at review-level (e.g., incomplete retrieval of identified research, reporting bias).	8
Conclusions	26	Provide a general interpretation of the results in the context of other evidence, and implications for future research.	9
**FUNDING**			
Funding	27	Describe sources of funding for the systematic review and other support (e.g., supply of data); role of funders for the systematic review.	9

From: Moher D, Liberati A, Tetzlaff J, Altman DG, The PRISMA Group (2009). Preferred Reporting Items for Systematic Reviews and Meta-Analyses: The PRISMA Statement. PLoS Med 6(7): e1000097. doi:10.1371/journal.pmed1000097

For more information, visit: **www.prisma-statement.org**.

For peer review only - http://bmjopen.bmj.com/site/about/guidelines.xhtml

BMJ Open

Health outcomes associated with Zika virus infection in humans: a systematic review of systematic reviews

Journal:	*BMJ Open*
Manuscript ID	bmjopen-2019-032275.R1
Article Type:	Original research
Date Submitted by the Author:	22-Aug-2019
Complete List of Authors:	Ximenes, Raphael; University Health Network, Toronto Health Economics and Technology Assessment (THETA) Collaborative, University Health Network<br>Ramsay, Lauren; University Health Network, Toronto Health Economics and Technology Assessment (THETA) Collaborative, University Health Network; University of Toronto, Institute of Health Policy, Management and Evaluation, University of Toronto<br>Miranda, Rafael; University Health Network, University Health Network, Toronto Health Economics and Technology Assessment (THETA) Collaborative, University Health Network<br>Morris, Shaun; Hospital fo sick children, Division of Infectious Diseases and Centre for Global Child Health, Hospital for Sick Children; University of Toronto Faculty of Medicine, Department of Pediatrics, Faculty of Medicine, University of Toronto<br>Murphy, Kellie; Mount Sinai Hospital<br>Sander, Beate; University Health Network, Toronto Health Economics and Technology Assessment (THETA) Collaborative, University Health Network; University of Toronto, Institute of Health Policy, Management and Evaluation, Management and Evaluation, University of Toronto
**Primary Subject Heading**:	Infectious diseases
Secondary Subject Heading:	Global health, Epidemiology, Obstetrics and gynaecology, Paediatrics, Neurology
Keywords:	Zika Virus infection, Epidemiology < INFECTIOUS DISEASES, Congenital Zika Syndrome, NEUROLOGY, Guillain-Barré syndrome, Microcephaly

SCHOLARONE™
Manuscripts

Health outcomes associated with Zika virus infection in humans: a systematic review of systematic reviews

Raphael Ximenes[1], Lauren C. Ramsay[1,2], Rafael N. Miranda[1], Shaun K. Morris[3,4], Kellie E. Murphy[5], RADAM-LAC Research Team, Beate Sander[1,2,6,7]

[1] Toronto Health Economics and Technology Assessment (THETA) Collaborative, University Health Network, Toronto, ON, Canada, raphael.ximenes@theta.utoronto.ca.

[2] Institute of Health Policy, Management and Evaluation, University of Toronto, Toronto, ON, Canada.

[3] Division of Infectious Diseases and Centre for Global Child Health, Hospital for Sick Children, Toronto, ON, Canada.

[4] Department of Pediatrics, Faculty of Medicine, University of Toronto, Toronto, ON, Canada.

[5] Mount Sinai Hospital, Toronto, ON, Canada.

[6] Public Health Ontario, Toronto, ON, Canada.

[7] ICES, Toronto, ON, Canada.

RADAM-LAC Research Team members are Beate Sander, Camila Gonzalez, Manisha Kulkarni, Marcos Miretti, Mauricio Espinel, Jianhong Wu and Varsovia Cevallos.

For peer review only

Abstract

Objective: With the emergence of Zika virus (ZIKV) disease in Central and South America in the mid-2010s and recognition of the teratogenic effects of congenital exposure to ZIKV, there has been a substantial increase in new research published on ZIKV. Our objective is to synthesize the literature on health outcomes associated with ZIKV infection in humans.

Methods: We conducted a systematic review (SR) of SRs following PRISMA (Preferred Reporting Items for Systematic Reviews and Meta-Analyses) guidelines. We searched MEDLINE, Embase, Cochrane and LILACS databases from inception to July 22, 2019, and included SRs that reported ZIKV associated health outcomes. Three independent reviewers selected eligible studies, extracted data and assessed the quality of included SRs using the A MeaSurement Tool to Assess Systematic Reviews 2 (AMSTAR 2) tool. Conflicts were resolved by consensus or consultation with a third reviewer.

Results: The search yielded 1,382 unique articles, of which 21 SRs met our inclusion criteria. The 21 SRs ranged from descriptive to quantitative data synthesis, including four meta-analysis. The most commonly reported ZIKV-associated manifestations and health outcomes were microcephaly, congenital abnormalities, brain abnormalities, neonatal death, and Guillain-Barré syndrome. The included reviews were highly heterogeneous. The overall quality of the SRs was critically low with all studies having more than one critical weakness.

Conclusion: The evolving nature of the literature on ZIKV-associated health outcomes, together with the critically low quality of existing SRs, demonstrate the need for high-quality SRs to guide patient care and inform policy decision making.

Strengths and limitations:

- Lack of SRs on ZIKV in the literature
- Lack of information about the risks of severe outcomes related to ZIKV infection or the presence of specific outcomes
- Broad search strategy
- Without restrictions by language or publication type
- To our knowledge, this is the first SR of SRs about health outcomes associated with ZIKV infection in humans

For peer review only

Introduction

Zika Virus (ZIKV) was first discovered in 1947 in rhesus monkeys in Uganda (1). It is an arbovirus in the flavivirus family and typically causes mild illness in humans characterized by fever and rash. There were reports of sporadic cases of ZIKV infection in humans over the years in Asia and Africa (2), with the first large documented outbreak taking place in Yap, a Micronesian island, in 2007 (3). Since then, there have been reported outbreaks in French Polynesia (in 2013-2014), and most recently in South and Central America and the Caribbean (4). With the emergence of ZIKV in Brazil, there were over 800,000 estimated cases of ZIKV infection reported by countries and territories in the Americas by January 2018 (5). By March 2017, according to the latest World Health Organization (WHO) global situation report on Zika, 84 countries, territories or subnational areas had evidence of vector-borne ZIKV transmission (6). According to the CDC, until May 2019, there were 89 areas with current or past transmission, but no current outbreak of ZIKV (7).

Our understanding of Zika-associated clinical outcomes has evolved over time. Before human pathogenesis was understood, cellular level damage was apparent in animal studies in the 1950s (8). The first study in humans to suggest an association between ZIKV and human disease was a case-control study during an outbreak in French Polynesia between 2013 and 2014, suggesting an association with Guillain-Barre Syndrome (GBS). (9). However, the link between ZIKV in pregnant women and microcephaly in infants was only evident in the 2015-2016 outbreak in South America (10). With the spread of ZIKV to new regions of the world and the extent of the outbreak in South and Central American and Caribbean countries, a substantial body of new research has been published in recent years about Zika.

A bibliometric analysis of ZIKV research that indexed in Web of Science found a significant increase in the number of studies being published beginning in 2015 (n=38 publications) to 2017 (n=1,962 publications) (11). Summarizing the large body of literature on outcomes associated with ZIKV infection is timely and needed.

The purpose of this systematic review (SR) of systematic reviews was to synthesize the currently known health outcomes associated with ZIKV infection in humans.

Methods

Search strategy and selection criteria

We searched MEDLINE, Embase, Cochrane and LILACS databases from inception to July 22, 2019. Our search strategy across all databases included concepts related to “Zika” and “systematic review” (complete search strategy found in Supplementary File 1). Our search strategy was not restricted by language or publication type. Three reviewers (RX, first reviewer; LR and RM second reviewers) independently screened titles, abstracts, and relevant full text of identified articles.

For peer review only

The inclusion criteria were defined as SRs that reported health outcomes of ZIKV infection in humans, i.e. clinical presentation and sequelae of ZIKV infection in humans. We excluded studies that only reported symptoms (e.g., rash, fever) of ZIKV infection, diagnostic techniques, mosquito control, therapeutic regimes, vaccine and trial but not outcomes (e.g., GBS, Congenital Zika Syndrome). We followed the Preferred Reporting Items for Systematic Reviews and Meta-Analyses (PRISMA) guidelines for reporting results (12).

The data extraction was performed in duplicate by the reviewers. The SR methods were established prior to the conduct of the SR and the protocol for the current SR was registered with PROSPERO (CRD42018091087) and there were no deviations from the protocol, except for adding the LILACS database to the search.

Patient and Public Involvement

No patient involved.

Quality appraisal

We used the A MeaSurement Tool to Assess systematic Reviews 2 (AMSTAR 2) tool to critically appraise the included SRs (13). AMSTAR 2 is not intended to generate an overall score, but rather to assist in the identification of high-quality SRs. Three reviewers (RX, first reviewer; LR and RM, second reviewers) independently evaluated the quality of each study based on weaknesses in critical domains as defined by the AMSTAR 2 tool. Studies were rated based on the overall confidence in the results of the SR and defined as either high (zero or one non-critical weakness), moderate (more than one non-critical weakness), low (one critical flaw with or without non-critical weaknesses) or critically low (more than one critical flaw with or without non-critical weaknesses) (14). Critical domains included protocol registration, adequacy of the literature search, justification for excluding studies, risk of bias from individual studies included in the SR, appropriateness of meta-analytical methods, consideration of risk of bias when interpreting results, and assessment of publication bias (14). Any disagreements between the two reviewers were resolved by consensus.

Data analysis

Three reviewers (RX, first reviewer; LR and RM, second reviewers) extracted the data using a structured electronic data extraction form, extracting study characteristics, and measures of effect for outcomes resulting from ZIKV infection. Included studies were summarized narratively, and health outcomes were reported where possible.

For peer review only

Results

We identified 1,382 unique articles from the database searches (Figure 1). After screening titles and abstracts, we selected 85 for full text screening. Of these, twenty-one met our inclusion criteria (15–35). The main reasons for exclusion at the full text stage were articles not being SRs (but rather overviews or literature reviews) and studies only reported symptoms but not outcomes.

Study characteristics are summarized in Table 1. The included SRs were published between February 2016 and May 2019. The types of studies eligible for inclusion in the SRs varied across studies; four SR did not include any information on the included studies (21,24,28,30), all other SRs included observational studies (one limited to only cohort studies (18)), and the majority (71%; n=15) included case reports and case series. Three SRs considered evidence from modelling studies, animal experiments, and in vitro experiments (15,33,35). Another did not limit to reports of primary data and included SRs, narrative reviews, and news articles (20).

The majority of studies included in the SRs were conducted in Brazil, the United States (US), French Polynesia and Colombia.

Summary of included SRs and outcomes

Of the 21 included SRs, the most commonly reported outcome was microcephaly, reported in 14 SRs (15–26,30,32), 12 SRs reported on GBS (15,16,19,20,22,23,25,27,29–31,33), 11 SRs reported on malformations or congenital abnormalities (18–20,22,26,30–34), 9 reported on brain (15,17,21,24–26,28,30,32), 7 SRs reported on ocular disorders (15,18,21,24,25,30,32), and 6 SRs on termination of pregnancy, fetal death and perinatal death (15,18–20,30,33). Three SRs or fewer reported on auditory disorder (15,26,34), cardiovascular damage (18,26,35), neurological complications (16,25,33), intrauterine growth restrictions (15,25), abnormal amniotic fluid (15), epilepsy (21), and death due Zika infection (16).

Seven SRs focused on pregnant women (17–20,24,26,28) and 5 SRs included the general population (15,16,22,23,29), while newborns, neonates, perinatal, early birth or infants were included in 5 five SRs (18,19,21,25,26). One SR focused in travelers returning to the US and Europe (31). Adults were the included in two of the 15 SRs (25,27).

Overlap between systematic reviews

Our SR includes 615 studies. Out of the 615 studies, 477 (77.56%) were cited only once in the included SRs, and the remainder were cited in up to 10 SRs, 83 (13.50%) were cited twice, 29 (4.72%) three times, 8 (1.30%) four times, 8 (1.30%) five times, 6 (0.98%) six times, 2 (0.33%) seven times, one (0.16%) eight times and one (0.16%) ten times (Table 2, Figure 2).

For peer review only

Health Outcomes

The Supplementary File 2 reports the health outcome data extracted from the twenty-one SRs.

Clinical Outcomes Associated with ZIKV Infection During Pregnancy

The Supplemental File 2 shows that the reported outcomes associated with ZIKV infection during pregnancy ranging from adverse birth outcomes to perinatal death. The frequency of infant deaths (miscarriages, perinatal deaths, intrauterine death or stillbirth and termination of pregnancy) was reported by 6 of 21 SRs (15,18–20,30,33), ranging from 4.8% to 22%.

Congenital Zika syndrome (CZS) was reported in many different ways. Some studies reported specific outcomes related to CZS (e.g. brain abnormalities, ocular disorder or microcephaly) while others reported CZS as a nonspecific outcome. The prevalence of CZS ranged from 2% (5 cases in 250 ZIKV-infected pregnant women) (18) to 50% (58 adverse congenital outcomes out of 117 women with PCR confirmed ZIKV) (22).

Brain abnormalities were explicitly reported with data from 19 studies in which 96% (205 in 213 pregnant women) of fetuses were diagnosed after confirmation with imaging tests (15). One SR reported the prevalence of brain abnormalities (28%) including microcephaly in newborns whose mothers were infected with ZIKV in pregnancy (25) while other SR reported an observational study of 35 infants with microcephaly, 11 fetuses had intra-uterine brain injury accompanied by stunting of cerebral growth prior to birth (17). Further, five SRs classified the type of brain abnormalities or where the lesions were found (21,24,28,30,32)as intracranial calcification, reduction in the constitution of gyri of the severe cerebral cortex, abnormal hypodensity of the white matter, malformations of cortical development, subcortical-cortical junction calcifications, basal ganglia calcification, brain calcification, intraventicular synechiae and periventricular cystic, brain volume loss, ventriculomegaly / hydrocephaly and diffuse involvement of all the cerebral lobes.

Microcephaly was reported in 14 of 21 SRs. Chibueze et al. (2016) provided a trimester-specific modeling estimate risk for microcephaly. When the infection occurs in an indeterminate period of pregnancy, ZIKV associated microcephaly was described by Coelho et al. (2017). The authors performed a meta-analysis and found a prevalence of 2.3% (95% CI 1% - 5.3%) of microcephaly when considering all pregnancies (2,941 mother-infant pairs). When considering only live births (2,648 live births), the prevalence of microcephaly was 2.7% (95% CI 1.2% - 6%) (18). Nithiyanantham et al. (2019) also performed a meta-analysis of the proportion of congenital disorders in infants born to ZIKV-infected mothers, reporting a prevalence of 3.9% (95% CI 2.4% – 5.4%) (32). Pomar et al. (2019) reported the prevalence of microcephaly in CZS ranging from 33.3% to 64% (30). Four SRs reported microcephaly cases per live-birth pregnancies, ranging from 0.2% (20 cases per 10,000 live births) to 14.3% (1 case in 7 live-birth pregnancies) (15,16,18,20) and one SRs reported 10 microcephaly cases per 10,000 births (19). Microcephaly risk in infected pregnant women was reported in four SRs. The absolute risk varied between 0.95% (95% CI: 0.34 – 1.91%) during the first trimester of pregnancy to 30% (22,24–26)

For peer review only

(trimester not reported). Death caused by microcephaly was estimated in a study reported by Coelho et al. (2017), reporting a rate of 8.3% (171 deaths among 2,063 confirmed cases of microcephaly) (18). The prevalence of microcephaly in asymptomatic ZIKV infection was also reported as 0.36% (0.22% – 0.51%) (23). Another SR reported that in a series of 13 infants with congenital ZIKV infection and microcephaly, more than half of the mothers did not report any symptoms of ZIKV prior to delivery (26).

The prevalence of congenital ZIKV syndrome-related outcomes is still unknown. In this SR of SRs we found the intrauterine growth restrictions rate reported varied from 28.57% (10 cases in 35 mother-infant pairs) (15) to 31.43%, from one observational study of 35 infants with microcephaly (17). Another study reported intrauterine growth restriction in 11.9% of fetuses with or without microcephaly (5 fetuses from 42 positives for ZIKV pregnant women) (25). Pomar et al. (2019) reported the prevalence of intrauterine growth restriction in 14% of CZS cases. The prevalence of ocular disorder was reported in five SRs ranging from 0.9% % (from one study with 395 live-birth pregnancies) to 58.6% (17 ocular findings with microcephaly associated in 29 infants) (15,18,21,24,25,30,32). Abnormal amniotic fluid was described only by Krauer et al. (2017). Auditory disorder was described by Krauer et al. (2018) (prevalence of 13% - 3 cases in 24 mother-infant pairs) and Soriano-Arandes et al. (2018) (prevalence of 7% - 5 cases in 70 children with laboratory diagnosis of ZIKV infection) and Barbosa et al. (2019) (variations in the frequency of altered otoacoustic emissions testing (OAE) and automated auditory brainstem (ABR) response testing across the studies in 515 children: altered OAE varied from 0% to 75%, while altered a-ABR varied from 0% to 29.2%). The prevalence of cardiovascular damage was reported by Coelho et al. (2017) (prevalence of 1% - 3 cases in 301 live-birth pregnancies), Soriano-Arandes et al. (2018) (prevalence of 13.6% - 14 cases in103 ZIKV cases) and Minhas et al. (2017) (prevalence of 67% of heart failure in a cohort with 9 adults positive for ZIKV and no previous cardiac history).

Neurological Complications Associated with ZIKV Infection

Neurological complications were reported by 12 of 21 SRs (16,19–23,25,27,29–31,33), where GBS was the most commonly reported neurological complication.

Among adults, the proportion of neurological complications associated with ZIKV infection in Bahia (Brazil) was similar to that in French Polynesia. Among these neurological complications, GBS was diagnosed in 1 of every 1,000 reported Zika cases in Brazil and 1.3 per 1,000 in French Polynesia (16). During the French Polynesia outbreak in 2013, the incidence of GBS has been 0.24 per 1,000 ZIKV infections (20), and Simões et al. (2016) described one case report in French Polynesia in which GBS was diagnosed in a patient with ZIKV (19).

Counotte et al. (2018) reported the increased incidence of GBS incidence ratio between during and pre-ZIKV outbreak periods in seven different countries; which ranged from 2.0 (95% CI: 1.6-2.6) to 9.8 (95% CI: 7.6-12.5), while Barbi et al. (2018) conducted a meta-analysis of the prevalence of GBS in ZIKV infected cases. Their estimate for the prevalence of GBS in adults infected with ZIKV was 1.23% (CI: 95% 1.17%-1.29%). This same study was reported by Pomar et al. (2019). Krauer et al. (2017) reported

the prevalence of symptomatic ZIKV in GBS cases (74-84% symptomatic ZIKV in GBS cases). Paixão et al. (2016), Padilla et al. (2016) and Barbi et al. (2018), described the prevalence of admission to an intensive care unit (ranging from 36% to 42%, among 42 and 38 GBS cases respectively) and mechanical ventilation (21% to 29% among 42 GBS cases) in French Polynesia. The interval between ZIKV and GBS symptoms was described by Krauer et al. (2017), Paixão et al. (2016), Padilla et al. (2016) and Counotte et al. (2018). The highest interval was reported by Paixão et al. (2016), where 88% of GBS cases reported a viral syndrome up to 23 days before the onset of the neurologic syndrome. No deaths due to GBS related with ZIKV infections were reported in this SR.

Epilepsy and sleep profiles were described in two SRs. For Marques et al. (2019), the prevalence of epilepsy in congenital ZIKV infants ranged from 42% (43 in 102 children with congenital ZIKV) to 67% (95 in 141 congenital ZIKV), and 34% (30 in 88 congenital ZIKV-infected children) of the ZIKV infected children were defined as poor sleepers (21). Pomar et al. (2019) reported that 9% to 95.5% of congenital ZIKV infections were associated with epilepsy.

Idiopatic thrombocytopaenia purpura (ITP) related with ZIKV infection was reported by Counotte et al. (2018). They reported 11 cases of ITP across 18 studies; however, there is no information about the total number of ZIKV infected subjects in these studies.

Deaths Associated with ZIKV Infection

Deaths due to Zika infection are rare. According to the Brazilian Ministry of Health, between 440,000 and 1,300,000 cases of Zika occurred in Brazil in 2015 (36,37). Since the beginning of the outbreak 11 deaths among adults were confirmed in Brazil and an additional nine deaths were reported by the countries and territories in the Americas (5).

Coinfection

Coinfection was reported with dengue (16–18,25), chikungunya (16,17,25) and HIV (16,17); cytomegalovirus, toxoplasmosis, or other known teratogenic agents (16–18); hepatitis B virus (HBV), hepatitis C virus (HCV), cytomegalovirus (CMV), herpes simplex virus (HSV), Epstein-Barr virus (EBV), rubella, human T lymphotrophic virus (HTLV), parvovirus B19 and syphilis (17).

Masel et al. (2019) found no association of prior exposure to DENV and fetal loss, or clinical neurological assessment of fetus, and no statistical difference in prior DENV exposed patients with or without GBS after ZIKV infection.

Quality assessment

Of the twenty-one SRs included, there was high inter-rater reliability between the reviewers (91%). The overall quality of the SRs was critically low with all studies identified as having more than one critical weakness with or without non-critical weaknesses (Figure 3). For all studies, the majority (65%) of answers for the six critical domains of AMSTAR 2 tool (questions 2, 4, 7, 9, 12 and 14) were 'no' or 'partial yes' (53% and 12% respectively) (Figure 4 and Supplementary File 3). Main weaknesses identified

For peer review only - http://bmjopen.bmj.com/site/about/guidelines.xhtml

were a deficient bibliographic search strategy and the lack of an explicit statement that SR methods were established prior to the conduct of the SR.

Discussion

Our SR of SRs identified 21 SRs that reported health outcomes associated with ZIKV infection. Microcephaly was the most commonly reported health outcome. Other outcomes reported were fetal death, neonatal death, congenital abnormalities including brain abnormalities, intrauterine growth restrictions, ocular disorders, and infant disorders including auditory disorders, cardiovascular damage, death due ZIKV infection, neurological complications, epilepsy and finally adult outcomes including GBS. The included SRs indicate that ZIKV infection is causally associated with congenital abnormalities, including microcephaly, and that ZIKV infection is a trigger of GBS, considering evidence on biological plausibility, the strength of association, and the exclusion of alternative explanations.

Overall, we found high heterogeneity among the twenty-one included SRs ranging from descriptive SRs, with few data on health outcomes associated with ZIKV infection, to more quantitative SRs, including four meta-analyses. There was some overlap (22%) of included studies across the SRs, indicating that the SRs are relatively distinct from each other and consistent with the included SRs reporting on different aspects of ZIKV infection. Given this heterogeneity it was not possible to perform a quantitative synthesis, making it difficult to compare the results or draw conclusions based on the included SRs. Further, our quality appraisal found that all SRs were of critically low quality, with only three or fewer of six critical domains of AMSTAR 2 tool met in any study.

Further research into the magnitude of effects, potential other immediate and late outcomes, and long-term sequelae is warranted to understand the full impact of ZIKV infection, particularly long-term follow up studies of infants born to ZIKV-infected mothers and infants and children infected with ZIKV early in life. In a recent study, Nielsen-Saines et al. (2019) reinforce this conclusion. They observed that the neurologic phenotype in some ZIKV-exposed children may change from abnormal to normal from birth into early childhood, and vice versa (38).

Our SR has some limitations. Since ZIKV is an emerging disease, and despite the increasing number of SRs, one limitation is the lack of SRs on ZIKV in the literature. Because the Brazilian outbreak prompted much of the recent research, 7 of 21 (33%) included SRs were conducted fairly early in the epidemic between 2016 and 2017, 43% in 2018 and 24% in 2019, which can explain the lack of information on severe outcomes related to ZIKV infection or the presence of specific outcomes, caused by the inability to observe outcomes that are only evident or possible to detect in older children. Often the reported data are unclear as to the nature of the infection, i.e. whether included subjects are suspected ZIKV cases or confirmed ZIKV cases. Further, some of the included SRs did not report denominators, making interpretation difficult.

For peer review only

The low quality of the included SRs may indicate an important publication bias related to rare outcomes such as ITP, and those poorly reported, but not rare, such as sleep disorders, epilepsy and auditory disorder.

Our study was strengthened by using a broad search strategy, without restrictions by language or publication type, reducing selection bias. To our knowledge, this is the first SR of SRs about health outcomes associated with ZIKV infection in humans.

As SRs of SRs aim to provide a summary of evidence from other SRs, although we were not able to perform a meta-analysis, our SR synthesizes findings from SRs on health outcomes associated with ZIKV infection in humans.

The evolving nature of the literature on ZIKV-associated health outcomes together with the critically low quality of existing SRs, confirm the need for high-quality SRs to better understand the burden of ZIKV, guide patient care and inform health policy.

Conclusion

Our SR demonstrates the need for future SRs on health outcomes associated with ZIKV infection as more research is published. As the ZIKV epidemic continues to evolve and the time since the emergence of the Brazilian outbreak increases we expect more primary observational studies on associated short- and long-term health outcomes to be published and synthesized in future SRs.

For peer review only

Acknowledgements

The authors would like to acknowledge Andrea Tricco and Joanna Bielecki for their guidance on this project.

Author Contributions

Raphael Ximenes: Conceptualization of the study, performed the systematic review, critically appraising the scientific literature, analysis, drafting and revising the manuscript.

Rafael N. Miranda: Performed the systematic review, critically appraising the scientific literature, revising the manuscript.

Lauren C. Ramsay: Performed the systematic review and critically appraising the scientific literature.

Shaun K. Morris: Critical revision of the manuscript.

Kellie E. Murphy: Critical revision of the manuscript.

RADAM-LAC Research Team: Contribution to study conception and design.

Beate Sander: Conceptualization of the study, critical revision of the manuscript, supervision of the study.

Funding
This project was funded by the Canadian Institutes of Health Research - Team grant - FRN149784 and the International Development Research Centre (IDRC).

Conflicts
The authors have no conflicts of interest to declare.

Data availability

All data underlining the results are available as part of the article and no additional source data are required.

For peer review only

References

1. World Health Organization. WHO | The History of Zika Virus. Who [Internet]. 2017 [cited 2018 Dec 10]; Available from: https://www.who.int/emergencies/zika-virus/timeline/en/

2. Posen HJ, Keystone JS, Gubbay JB, Morris SK. Epidemiology of Zika virus, 1947–2007. BMJ Glob Heal [Internet]. 2016 Aug [cited 2019 Feb 9];1(2):e000087. Available from: http://www.ncbi.nlm.nih.gov/pubmed/28588942

3. Duffy MR, Chen T-H, Hancock WT, Powers AM, Kool JL, Lanciotti RS, et al. Zika Virus Outbreak on Yap Island, Federated States of Micronesia. N Engl J Med [Internet]. 2009 Jun 11 [cited 2018 Dec 10];360(24):2536–43. Available from: http://www.nejm.org/doi/abs/10.1056/NEJMoa0805715

4. Kindhauser MK, Allen T, Frank V, Santhana RS, Dye C. Zika: the origin and spread of a mosquito-borne virus. Bull World Health Organ [Internet]. 2016 Sep 1;94(9):675–686C. Available from: http://www.who.int/entity/bulletin/volumes/94/9/16-171082.pdf

5. Pan American Health Organization / World Health Organization. Zika suspected and confirmed cases reported by countries and territories in the Americas Cumulative cases, 2015-2017. Updated as of 04 January 2018 [Internet]. Pan American Health Organization. Washington, D.C.; 2017 [cited 2019 Feb 9]. Available from: https://www.paho.org/hq/index.php?option=com_docman&view=download&category_slug=cumulative-cases-pdf-8865&alias=43296-zika-cumulative-cases-4-january-2018-296&Itemid=270&lang=en

6. World Health Organization. SITUATION REPORT ZIKA VIRUS MICROCEPHALY GUILLAIN-BARRÉ SYNDROME 10 MARCH 2017 DATA AS OF 9 MARCH 2017 [Internet]. [cited 2019 Feb 9]. Available from: https://apps.who.int/iris/bitstream/handle/10665/254714/zikasitrep10Mar17-eng.pdf?sequence=1

7. CDC. Zika Travel Information | Travelers' Health | CDC [Internet]. [cited 2019 May 28]. Available from: https://wwwnc.cdc.gov/travel/page/zika-travel-information

8. Dick GW. Zika virus (II). Pathogenicity and physical properties. Trans R Soc Trop Med Hyg [Internet]. 1952 Sep 1 [cited 2019 May 28];46(5):521–34. Available from: https://academic.oup.com/trstmh/article-lookup/doi/10.1016/0035-9203(52)90043-6

9. Cao-Lormeau VM, Blake A, Mons S, Lastere S, Roche C, Vanhomwegen J, et al. Guillain-Barre Syndrome outbreak associated with Zika virus infection in French Polynesia: a case-control study. Lancet. 2016;387:1531–9.

10. de Araújo TVB, Rodrigues LC, de Alencar Ximenes RA, de Barros Miranda-Filho D, Montarroyos UR, de Melo APL, et al. Association between Zika virus infection and microcephaly in Brazil, January to May, 2016: preliminary report of a case-control study. Lancet Infect Dis [Internet]. 2016 Dec 1 [cited 2019 Jan 28];16(12):1356–63. Available from: http://www.ncbi.nlm.nih.gov/pubmed/27641777

11. Saima Nasir JA. A Bibliometric Analysis of Research on Zika Virus Indexed in Web of Science. Adv Life Sci [Internet]. 2018 [cited 2018 Dec 4];5(3):88–95. Available from: http://www.als-journal.com/532-18/

For peer review only

12. Moher D, Shamseer L, Clarke M, Ghersi D, Liberatî A, Petticrew M, et al. Preferred reporting items for systematic review and meta-analysis protocols (PRISMA-P) 2015 statement [Internet]. 2015. Available from: http://www.crd.york.ac.uk/prospero

13. AMSTAR - Assessing the Methodological Quality of Systematic Reviews [Internet]. [cited 2018 Dec 4]. Available from: https://amstar.ca/Amstar-2.php

14. Shea BJ, Reeves BC, Wells G, Thuku M, Hamel C, Moran J, et al. AMSTAR 2: a critical appraisal tool for systematic reviews that include randomised or non-randomised studies of healthcare interventions, or both. BMJ [Internet]. 2017 Sep 21 [cited 2018 Dec 4];j4008. Available from: http://www.bmj.com/lookup/doi/10.1136/bmj.j4008

15. Krauer F, Riesen M, Reveiz L, Oladapo OT, Martínez-Vega R, Porgo T V., et al. Zika Virus Infection as a Cause of Congenital Brain Abnormalities and Guillain–Barré Syndrome: Systematic Review. von Seidlein L, editor. PLOS Med [Internet]. 2017 Jan 3;14(1):e1002203. Available from: https://dx.plos.org/10.1371/journal.pmed.1002203

16. Paixão ES, Barreto F, Teixeira M da G, Costa M da CN, Rodrigues LC. History, Epidemiology, and Clinical Manifestations of Zika: A Systematic Review. Am J Public Health [Internet]. 2016 Apr 9;106(4):606–12. Available from: http://ajph.aphapublications.org/doi/10.2105/AJPH.2016.303112

17. Chibueze EC, Tirado V, Lopes K da S, Balogun OO, Takemoto Y, Swa T, et al. Zika virus infection in pregnancy: a systematic review of disease course and complications. Reprod Health [Internet]. 2017 Dec 28;14(1):28. Available from: http://reproductive-health-journal.biomedcentral.com/articles/10.1186/s12978-017-0285-6

18. Coelho A, Crovella S, Coelho AVC, Crovella S. Microcephaly Prevalence in Infants Born to Zika Virus-Infected Women: A Systematic Review and Meta-Analysis. Int J Mol Sci [Internet]. 2017 Aug 5;18(8):1714. Available from: http://www.mdpi.com/1422-0067/18/8/1714

19. Simões R, Buzzini R, Bernardo W, Cardoso F, Salomão A, Cerri G, et al. Zika virus infection and pregnancy. Rev Assoc Med Bras [Internet]. 2016 Apr;62(2):108–15. Available from: http://www.scielo.br/scielo.php?script=sci_arttext&pid=S0104-42302016000200108&lng=en&tlng=en

20. Padilla C, Pan A, Geller A, Zakowski MI. Zika virus: review and obstetric anesthetic clinical considerations. J Clin Anesth [Internet]. 2016 Dec 1;35:136–44. Available from: https://www.sciencedirect.com/science/article/pii/S0952818016304299

21. Marques V de M, Santos CS, Santiago IG, Marques SM, Nunes Brasil M das G, Lima TT, et al. Neurological Complications of Congenital Zika Virus Infection. Pediatr Neurol [Internet]. 2019 Feb;91:3–10. Available from: http://www.ncbi.nlm.nih.gov/pubmed/30591235

22. Counotte MJ, Egli-Gany D, Riesen M, Abraha M, Porgo TV, Wang J, et al. Zika virus infection as a cause of congenital brain abnormalities and Guillain-Barré syndrome: From systematic review to living systematic review. PLoS Med [Internet]. 2018 Feb 15;7:196. Available from: http://www.ncbi.nlm.nih.gov/pubmed/30631437

23. Haby MM, Pinart M, Elias V, Reveiz L. Prevalence of asymptomatic Zika virus infection: a systematic review. Bull World Health Organ [Internet]. 2018 Jun 1;96(6):402–413D. Available

For peer review only

from: http://www.ncbi.nlm.nih.gov/pubmed/29904223

24. Rehan Sarwar M, Saqib A, Iftikhar S. Zika Virus Infection during Pregnancy; Maternofetal Risk Assessment, Transmission, Complications, and Management: A Review of the Literature. Arch Clin Infect Dis [Internet]. 2018 Jun 24;13(3). Available from: http://archcid.com/en/articles/12848.html

25. Wahid B, Ali A, Waqar M, Idrees M. An updated systematic review of Zika virus-linked complications. Asian Pac J Trop Med [Internet]. 2018;11(1):1. Available from: http://www.apjtm.org/text.asp?2018/11/1/1/223527

26. Soriano-Arandes A, Rivero-Calle I, Nastouli E, Espiau M, Frick M, Alarcon A, et al. What we know and what we don't know about perinatal Zika virus infection: a systematic review. Expert Rev Anti Infect Ther [Internet]. 2018 Mar 4;16(3):243–54. Available from: http://www.ncbi.nlm.nih.gov/pubmed/29415586

27. Barbi L, Coelho AVC, Alencar LCA de, Crovella S. Prevalence of Guillain-Barré syndrome among Zika virus infected cases: a systematic review and meta-analysis. Brazilian J Infect Dis [Internet]. 2018 Mar;22(2):137–41. Available from: http://www.ncbi.nlm.nih.gov/pubmed/29545017

28. Santos GRB dos, Aragão FBA, Lobão WJ de M, Lima FR, Andrade LMRL de, Furtado QR, et al. Relationship between microcephaly and Zika virus during pregnancy: a review. Rev Assoc Med Bras [Internet]. 2018 Jul;64(7):635–42. Available from: http://www.ncbi.nlm.nih.gov/pubmed/30365666

29. Wachira VK, Peixoto HM, Fernandes De Oliveira MR. Systematic review of factors associated with the development of Guillain-Barr e syndrome 2007-2017: what has changed? 2018; Available from: https://v2dis-prod.evidencepartners.com/Generic/getAttachment2.php?id=44

30. Pomar L, Musso D, Malinger G, Vouga M, Panchaud A, Baud D. Zika virus during pregnancy: From maternal exposure to congenital Zika virus syndrome. Prenat Diagn [Internet]. 2019 May [cited 2019 Aug 1];39(6):420–30. Available from: http://www.ncbi.nlm.nih.gov/pubmed/30866073

31. Wilder-Smith A, Chang CR, Leong WY. Zika in travellers 1947-2017: a systematic review. J Travel Med [Internet]. 2018 [cited 2019 Aug 1];25(1). Available from: http://www.ncbi.nlm.nih.gov/pubmed/30016469

32. Nithiyanantham SF, Badawi A. Maternal infection with Zika virus and prevalence of congenital disorders in infants: systematic review and meta-analysis. Can J Public Heal [Internet]. 2019 May 10 [cited 2019 Aug 1]; Available from: http://www.ncbi.nlm.nih.gov/pubmed/31077071

33. Masel J, McCracken MK, Gleeson T, Morrison B, Rutherford G, Imrie A, et al. Does prior dengue virus exposure worsen clinical outcomes of Zika virus infection? A systematic review, pooled analysis and lessons learned. Diemert DJ, editor. PLoS Negl Trop Dis [Internet]. 2019 Jan 25 [cited 2019 Aug 1];13(1):e0007060. Available from: http://www.ncbi.nlm.nih.gov/pubmed/30682026

34. Barbosa MH de M, Magalhães-Barbosa MC de, Robaina JR, Prata-Barbosa A, Lima MA de MT de, Cunha AJLA da. Auditory findings associated with Zika virus infection: an integrative review. Braz J Otorhinolaryngol [Internet]. 2019 Jun 18 [cited 2019 Aug 1]; Available from: http://www.ncbi.nlm.nih.gov/pubmed/31296482

For peer review only

35. Minhas AM, Nayab A, Iyer S, Narmeen M, Fatima K, Khan MS, et al. Association of Zika Virus with Myocarditis, Heart Failure, and Arrhythmias: A Literature Review. Cureus [Internet]. 2017 Jun 27 [cited 2019 Aug 1];9(6):e1399. Available from: http://www.ncbi.nlm.nih.gov/pubmed/28856072

36. Anthony Boadle LP. Exclusive: Brazil says Zika virus outbreak worse than believed | Reuters [Internet]. Reuters. 2016 [cited 2018 Dec 4]. Available from: https://www.reuters.com/article/us-health-zika-brazil-exclusive-idUSKCN0VA331

37. World Health Organization. ZIKA SITUATION REPORT - ZIKA AND POTENTIAL COMPLICATIONS 12 FEBRUARY 2016 [Internet]. 2016 [cited 2018 Dec 4]. Available from: https://www.who.int/emergencies/zika-virus/situation-report/who-zika-situation-report-12-02-2016.pdf

38. Nielsen-Saines K, Brasil P, Kerin T, Vasconcelos Z, Gabaglia CR, Damasceno L, et al. Delayed childhood neurodevelopment and neurosensory alterations in the second year of life in a prospective cohort of ZIKV-exposed children. Nat Med [Internet]. 2019 Aug 8 [cited 2019 Aug 14];25(8):1213–7. Available from: http://www.nature.com/articles/s41591-019-0496-1

39. Brasil P, Pereira JP, Moreira ME, Ribeiro Nogueira RM, Damasceno L, Wakimoto M, et al. Zika Virus Infection in Pregnant Women in Rio de Janeiro. N Engl J Med [Internet]. 2016 Dec 15 [cited 2019 Aug 6];375(24):2321–34. Available from: http://www.ncbi.nlm.nih.gov/pubmed/26943629

40. Mlakar J, Korva M, Tul N, Popović M, Poljšak-Prijatelj M, Mraz J, et al. Zika Virus Associated with Microcephaly. N Engl J Med [Internet]. 2016 Mar 10 [cited 2019 Aug 6];374(10):951–8. Available from: http://www.ncbi.nlm.nih.gov/pubmed/26862926

41. de Paula Freitas B, de Oliveira Dias JR, Prazeres J, Sacramento GA, Ko AI, Maia M, et al. Ocular Findings in Infants With Microcephaly Associated With Presumed Zika Virus Congenital Infection in Salvador, Brazil. JAMA Ophthalmol [Internet]. 2016 May 1 [cited 2019 Aug 6];134(5):529. Available from: http://www.ncbi.nlm.nih.gov/pubmed/26865554

42. Schuler-Faccini L, Ribeiro EM, Feitosa IML, Horovitz DDG, Cavalcanti DP, Pessoa A, et al. Possible Association Between Zika Virus Infection and Microcephaly — Brazil, 2015. MMWR Morb Mortal Wkly Rep [Internet]. 2016 Jan 29 [cited 2019 Aug 6];65(3):59–62. Available from: http://www.ncbi.nlm.nih.gov/pubmed/26820244

43. Cao-Lormeau V-M, Blake A, Mons S, Lastère S, Roche C, Vanhomwegen J, et al. Guillain-Barré Syndrome outbreak associated with Zika virus infection in French Polynesia: a case-control study. Lancet [Internet]. 2016 Apr 9 [cited 2018 Dec 4];387(10027):1531–9. Available from: https://www.sciencedirect.com/science/article/pii/S0140673616005626

44. Honein MA, Dawson AL, Petersen EE, Jones AM, Lee EH, Yazdy MM, et al. Birth Defects Among Fetuses and Infants of US Women With Evidence of Possible Zika Virus Infection During Pregnancy. JAMA [Internet]. 2017 Jan 3 [cited 2019 Aug 6];317(1):59. Available from: http://jama.jamanetwork.com/article.aspx?doi=10.1001/jama.2016.19006

45. Meaney-Delman D, Hills SL, Williams C, Galang RR, Iyengar P, Hennenfent AK, et al. Zika Virus Infection Among U.S. Pregnant Travelers — August 2015–February 2016. MMWR Morb Mortal Wkly Rep [Internet]. 2016 Mar 4 [cited 2019 Aug 6];65(8):211–4. Available from: http://www.ncbi.nlm.nih.gov/pubmed/26938703

For peer review only

46. Cauchemez S, Besnard M, Bompard P, Dub T, Guillemette-Artur P, Eyrolle-Guignot D, et al. Association between Zika virus and microcephaly in French Polynesia, 2013–15: a retrospective study. Lancet [Internet]. 2016 May 21 [cited 2019 Aug 6];387(10033):2125–32. Available from: http://www.ncbi.nlm.nih.gov/pubmed/26993883

47. Oliveira Melo AS, Malinger G, Ximenes R, Szejnfeld PO, Alves Sampaio S, Bispo de Filippis AM. Zika virus intrauterine infection causes fetal brain abnormality and microcephaly: tip of the iceberg? Ultrasound Obstet Gynecol [Internet]. 2016 Jan 1 [cited 2019 Aug 6];47(1):6–7. Available from: http://doi.wiley.com/10.1002/uog.15831

48. Besnard M, Eyrolle-Guignot D, Guillemette-Artur P, Lastère S, Bost-Bezeaud F, Marcelis L, et al. Congenital cerebral malformations and dysfunction in fetuses and newborns following the 2013 to 2014 Zika virus epidemic in French Polynesia. Eurosurveillance [Internet]. 2016 Mar 31 [cited 2019 Aug 6];21(13):30181. Available from: http://www.ncbi.nlm.nih.gov/pubmed/27063794

49. van der Linden V, Pessoa A, Dobyns W, Barkovich AJ, Júnior H van der L, Filho ELR, et al. Description of 13 Infants Born During October 2015–January 2016 With Congenital Zika Virus Infection Without Microcephaly at Birth — Brazil. MMWR Morb Mortal Wkly Rep [Internet]. 2016 Dec 2 [cited 2019 Aug 6];65(47):1343–8. Available from: http://www.cdc.gov/mmwr/volumes/65/wr/mm6547e2.htm

50. Soares de Oliveira-Szejnfeld P, Levine D, Melo AS de O, Amorim MMR, Batista AGM, Chimelli L, et al. Congenital Brain Abnormalities and Zika Virus: What the Radiologist Can Expect to See Prenatally and Postnatally. Radiology [Internet]. 2016 Oct [cited 2019 Aug 6];281(1):203–18. Available from: http://www.ncbi.nlm.nih.gov/pubmed/27552432

51. Calvet G, Aguiar RS, Melo ASO, Sampaio SA, de Filippis I, Fabri A, et al. Detection and sequencing of Zika virus from amniotic fluid of fetuses with microcephaly in Brazil: a case study. Lancet Infect Dis [Internet]. 2016 Jun [cited 2019 Aug 6];16(6):653–60. Available from: http://www.ncbi.nlm.nih.gov/pubmed/26897108

52. França GVA, Schuler-Faccini L, Oliveira WK, Henriques CMP, Carmo EH, Pedi VD, et al. Congenital Zika virus syndrome in Brazil: a case series of the first 1501 livebirths with complete investigation. Lancet [Internet]. 2016 Aug 27 [cited 2019 Aug 6];388(10047):891–7. Available from: http://www.ncbi.nlm.nih.gov/pubmed/27372398

53. Besnard M, Lastère S, Teissier A, Cao-Lormeau V, Musso D. Evidence of perinatal transmission of Zika virus, French Polynesia, December 2013 and February 2014. Eurosurveillance [Internet]. 2014 Apr 3 [cited 2019 Aug 6];19(13):20751. Available from: http://www.eurosurveillance.org/ViewArticle.aspx?ArticleId=20751

54. Kleber de Oliveira W, Cortez-Escalante J, De Oliveira WTGH, do Carmo GMI, Henriques CMP, Coelho GE, et al. Increase in Reported Prevalence of Microcephaly in Infants Born to Women Living in Areas with Confirmed Zika Virus Transmission During the First Trimester of Pregnancy — Brazil, 2015. MMWR Morb Mortal Wkly Rep [Internet]. 2016 Mar 11 [cited 2019 Aug 6];65(9):242–7. Available from: http://www.ncbi.nlm.nih.gov/pubmed/26963593

55. Oehler E, Watrin L, Larre P, Leparc-Goffart I, Lastere S, Valour F, et al. Zika virus infection complicated by Guillain-Barre syndrome--case report, French Polynesia, December 2013. Euro Surveill [Internet]. 2014 Mar 6 [cited 2019 Aug 16];19(9). Available from:

For peer review only

http://www.ncbi.nlm.nih.gov/pubmed/24626205

For peer review only

Table 1. Summary of included systematic reviews

Author, Year	Aim	Search period	Number of studies included	Types of studies included in review	Jurisdictions of included studies (n studies)
Krauer et al. (2017) (15)	To assess the relationship between ZIKV infection and congenital brain abnormalities and Guillain-Barré syndrome	From inception until May 30, 2016	106	Case reports, case series, case-control studies, cohort studies, cross-sectional studies, ecological study/outbreak reports, modelling studies, animal experiments, in vitro experiments, sequence analysis and phylogenetics	Brazil (6), Cabo Verde (2), Colombia (1), French Polynesia (2), Martinique (2), Panama (5), El Salvador (1), Haiti (119), Puerto Rico (1), Venezuela (1), Slovenia*, Netherlands*, Dominican Republic*, French Guiana*, Honduras*, Paraguay*, Suriname*, Micronesia*, Pacific Islands*<br>* Not possible to know number of studies from these countries
Paixão et al. (2016) (16)	To summarize current knowledge on ZIKV including epidemiology, clinical presentation, and complications	1954 to Jan 2016	41	Case reports, case series, surveillance reports, cross-sectional studies, epidemiological bulletins and alerts	Not clearly reported.<br>Most data are from Brazil and French Polynesia.
Chibueze et al. (2017) (17)	To summarize guidance on pregnancy care in the context of ZIKV infection	From inception until March 3, 2016	18	Case reports, case series, observational studies	Brazil (11)<br>Colombia (1)<br>France (1)<br>Puerto Rico (1)<br>Slovenia (1)<br>USA (2)<br>Venezuela (1)
Coelho et al. (2017) (18)	To summarize evidence and meta-analyze data to estimate prevalence	Not reported	8	Cohort studies	Brazil (1)<br>Colombia (1)<br>French Guiana (1)<br>Puerto Rico (1)

For peer review only

Author, Year	Aim	Search period	Number of studies included	Types of studies included in review	Jurisdictions of included studies (n studies)
	of microcephaly in babies born to ZIKV infected pregnant women				USA (4)
Simões et al. (2016) (19)	To assess the effects of Zika virus infection on during pregnancy and postpartum periods	From inception until Feb 23, 2016	30	Case reports, case series, guidelines	Not clearly reported; most data are from Brazil.
Padilla et al. (2016) (20)	To review clinical and basic science literature about ZIKV infection relevant for obstetric anesthesiologists	From inception until Apr 15, 2016	30	Systematic reviews, narrative reviews, case reports, epidemiologic studies, government reports, and news articles	Not clearly reported.
Marques et al. (2019) (21)	To map the neurological damage and outcomes related to congenital ZIKV infection	Jan 1966 to Aug 2018	28	Not informed	Brazil (16)<br>USA (3)<br>Colombia (1)
Counotte et al. (2018) (22)	To summarize the evidence of the casual associations between ZIKV and CZS and GBS	May 30, 2016 to Jan 18, 2017	101	Case report, case series, case-control study, cohort study, cross-sectional study, controlled trials, ecological study/outbreak report, modelling study, animal experiment, in vitro experiment, sequencing and phylogenetics, biochemical/protein structure studies	USA, Martinique, Brazil, Suriname, Colombia, French Guiana, Slovenia, Spain, Uganda, Nicaragua, Barbados, Belize, Dominican Republic, El Salvador, Guatemala, Haiti, Honduras; Mexico, Republic of Marshall Islands, Venezuela, French Polynesia, Ecuador, France, Puerto Rico, Guadeloupe,

For peer review only

Author, Year	Aim	Search period	Number of studies included	Types of studies included in review	Jurisdictions of included studies (n studies)
					Guyana, New Zealand, French Southern Territories
Haby et al., (2018) (23)	To estimate and meta-analyze the prevalence of asymptomatic Zika virus infection in the general population and in specific population groups from observational epidemiological studies	From inception until Jan 26, 2018	23	Cross-sectional seroprevalence studies, case series, case-control, cohort	USA (6), Brazil (3), French Polynesia (3), French Guiana (3), Puerto Rico (2), Colombia (2), Spain (2), Micronesia (1), Martinique (1)
Sarwar et al. (2018) (24)	To report on the current literature regarding ZIKV and its hazardous effects on maternofetal health with a special emphasis on risk assessment, virus transmission, associated complications, and possible management	2007 to May 2017	69	Not informed	Argentina, Bolivia, Brazil, Colombia, French Guiana, Suriname, Paraguay, Trinidad and Tobago, Canada, Dominican Republic, Grenada, Guadeloupe, Guatemala, Haiti, Martinique, Puerto Rico, USA, Costa Rica, El Salvador, Honduras, Nicaragua, Panama, Europe, Slovenia, Spain, Thailand, Vietnam, French Polynesia, Marshall Islands, Cape Verde
Wahid et al. (2018) (25)	To summarize the evidence of neurological complications in ZIKV-infected people	2015 to March 2017	35	Case-studies, case-cohort studies, cross-sectional studies, organizational survey reports and case-control studies	Brazil (15)<br>French Polynesia (4)<br>Colombia (3)

For peer review only

**Author, Year**	**Aim**	**Search period**	**Number of studies included**	**Types of studies included in review**	**Jurisdictions of included studies (n studies)**
					USA, Slovenia, Suriname, Spain, Haiti, Martinique, Netherlands, Ecuador, Guyana (1)
Soriano-Arandes et al. (2018) (26)	To summarize the new evidence in aspects of epidemiology, virology, pathogenesis, associated risk factors during pregnancy, newborn phenotypic signs, neuroimaging, laboratory diagnosis, treatment and vaccines	From inception until Nov 30, 2017	106	Case series, cohort (prospective/retrospective), cross-sectional or case-control studies	Brazil, French Polynesia, USA, Martinique, Colombia
Barbi et al. (2018) (27)	To systematically review the literature and perform a meta-analysis to estimate the prevalence of GBS among ZIKV-infected individuals	From inception until Nov 2017	3	Case series, epidemiological surveys, cross-sectional or cohort studies	French Polynesia (1), Suriname and Dominican Republic (1), South American, Central American and Caribbean countries (1)
Santos et al. (2018) (28)	To analyze the association between Zika-virus and microcephaly during the gestational period	From inception until Dec 2016	35	Not informed	Brazil
Wachira et al. (2018) (29)	To describe the factors associated with development of GBS, both infectious and	Jan 1, 2007 to Jun 30, 2017	34	The most common were case control, cohort, self-controlled case series	French Polynesia

For peer review only

Author, Year	Aim	Search period	Number of studies included	Types of studies included in review	Jurisdictions of included studies (n studies)
	non-infectious, through a SR.				
Pomar et al. (2019) (30)	Present a review to describe the risks and complications of maternal and subsequent fetal infection by ZIKV.	Jun 2009 to Nov 2018	68	Not informed	Colombia (3), Puerto Rico (1), French Guiana (3), Brazil (1), Yap Island (1), USA (2)
Wilder-Smith et al. (2018) (31)	Describe the burden of ZIKV infection in international travelers over time; estimate the proportion of birth defects as a result of maternal ZIKV infection in travelers; track the extent of sexual transmission; summarize data on ZIKV cases in travelers identifying counties with reports on local transmission	1947 to Apr 2017	65	Surveillance reports, case reports, retrospective (multi-centre study), descriptive retrospective analysis and prospective cohort study	USA (9), Canada (2), Germany (3), Norway (1), France (5), Italy (7), Japan (2), Australia (4), New Caledonia (1), Finland (1), Mexico (1), Slovenia (1), Netherlands (4), Belgium (1), Portugal (1), Switzerland (3), Israel (1), Taiwan (2), Spain (1), China (7), South Korea (2), UK (2), Singapore (1), Malaysia (1)
Nithiyanantham et al. (2019) (32)	To conduct a systematic review and meta-analysis on the prevalence of congenital Zika-related disorders in infants of mothers	From inception until Oct 31, 2017	25	Case series, epidemiological reports, prospective and retrospective studies, cohort studies and cross-sectional studies	USA (8), Brazil (6), Colombia (2), Puerto Rico (1), French Polynesia (1), Martinique (1), Trinidad and Tobago (1), French Guiana (1), Ecuador (1), Spain (1)

For peer review only

Author, Year	Aim	Search period	Number of studies included	Types of studies included in review	Jurisdictions of included studies (n studies)
	infected with ZIKV during pregnancy.				
Masel et al. (2019) (33)	To determine if prior infection with DENV, as compared with those with no prior DENV infection, is associated with a greater risk of ZIKV complications (including neurological and teratogenic outcomes), greater ZIKV peak viremia, greater area-under-the-curve of viremia or other putative laboratory proxies of ZIKV severity.	From inception until Mar 25, 2018	5	Case control study	Brazil (2), French Polynesia (5)
Barbosa et al. (2019) (34)	To describe the auditory alterations, pathogenesis and recommendations for follow-up in individuals with prenatal or acquired ZIKV infection.	From inception until Apr 2019	27	Case report and case series	Brazil (14), Colombia (3), USA (2), French Polynesia (1), Puerto Rico (1)
Minhas et al. (2017) (35)	Focuses on the potential threat that ZIKV may pose to the heart like that of	From inception until March 2017	3	Case report and prospective observational multicenter study	France (1), Venezuela (1), China (1)

For peer review only

Author, Year	Aim	Search period	Number of studies included	Types of studies included in review	Jurisdictions of included studies (n studies)
	similar arboviral diseases.				

For peer review only

Table 2. Overlap between systematic reviews

Number of citations	Title	Author	Cited by
10	Zika virus infection in pregnant women in Rio de Janeiro	Brasil et al. (2016) (39)	(15,17,18,20–22,25,26,30,32)
8	Zika virus associated with microcephaly	Mlakar et al. (2016) (40)	(15,17,19,24–26,28,31)
7	Ocular findings in infants with microcephaly associated with presumed Zika virus congenital infection in Salvador, Brazil	de Paula Freitas et al. (2016) (41)	(15,17,19–21,25,30)
7	Possible association between Zika virus infection and microcephaly - Brazil, 2015	Schuler-Faccini et al. (2016) (42)	(15,17,21,25,26,28,30)
6	Guillain-Barre Syndrome outbreak associated with Zika virus infection in French Polynesia: a case-control study	Cao-Lormeau et al. (2016) (43)	(15,20,23–25,33)
6	Birth defects among fetuses and infants of US women with evidence of possible Zika virus infection during pregnancy	Honein et al. (2017) (44)	(18,21,22,24,26,32)
6	Zika virus infection among U.S. pregnant travelers - August 2015 - February 2016	Meaney-Delman et al. (2016) (45)	(15,17,18,20,31,32)
6	Zika virus outbreak on Yap Island, Federated States of Micronesia	Duffy et al. (2009) (3)	(15,16,19,23,24,30)
6	Association between Zika virus and microcephaly in French Polynesia, 2013 - 15: a retrospective study	Cauchemez et al. (2016) (46)	(15,17,24–26,30)
6	Zika virus intrauterine infection causes fetal brain abnormality and microcephaly: tip of the iceberg?	Oliveira et al. (2016) (47)	(15,17,20,21,26,28)
5	Congenital cerebral malformations and dysfunction in fetuses and newborns following the 2013 to 2014 Zika virus epidemic in French Polynesia	Besnard et al. (2016) (48)	(15,25,30,32,34)
5	Description of 13 infants born during October 2015 - January 2016 with congenital Zika virus infection without microcephaly at birth - Brazil	van der Linden et al. (2016) (49)	(21,22,26,30,34)
5	Congenital brain abnormalities and Zika virus: what the radiologist can expect to see prenatally and postnatally	Oliveira-Szejnfeld et al. (2016) (50)	(21,22,26,30,32)
5	Detection and sequencing of Zika virus from amniotic fluid of fetuses with microcephaly in Brazil: a case study	Calvet et al. (2016) (51)	(15,17,19,28,30)

For peer review only

5	Congenital Zika virus syndrome in Brazil: a case series of the first 1501 livebirths with complete investigation	França et al. (2016) (52)	(21,22,25,26,30)
5	Evidence of perinatal transmission of Zika virus, French Polynesia, December 2013 and February 2014	Besnard et al. (2014) (53)	(16,17,24,26,28)
5	Increase in reported prevalence of microcephaly in infants born to women living in areas with confirmed Zika virus transmission during the first trimester of pregnancy - Brazil, 2015	Oliveira et al. (2016) (54)	(15,17,20,24,25)
5	Zika virus infection complicated by Guillain-Barre syndrome - case report, French Polynesia, December 2013	Oehler et al. (2014) (55)	(15,16,19,20,25)

Figure legends

Figure 1: PRISMA flow diagram of search results and study selection.

Figure 2: Overlap between studies cited in at least 5 systematic reviews.

Figure 3: Individual study results of quality assessment using AMSTAR 2 - Result for all questions of AMSTAR 2 tool.

Figure 4: Individual study results of quality assessment using AMSTAR 2 - Critical domains of AMSTAR 2 tool.

For peer review only

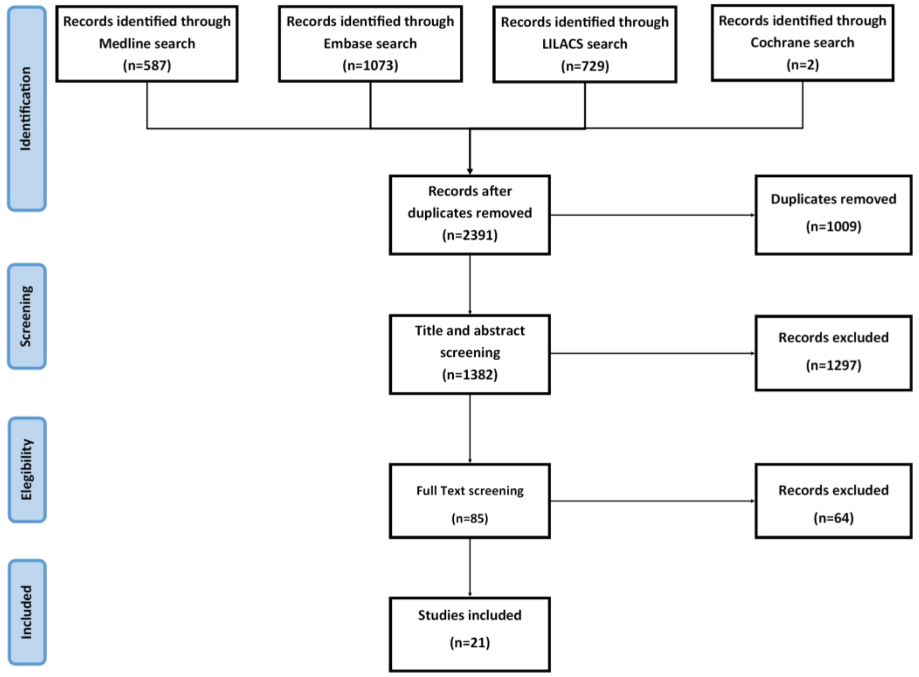

PRISMA flow diagram of search results and study selection.

279x215mm (300 x 300 DPI)

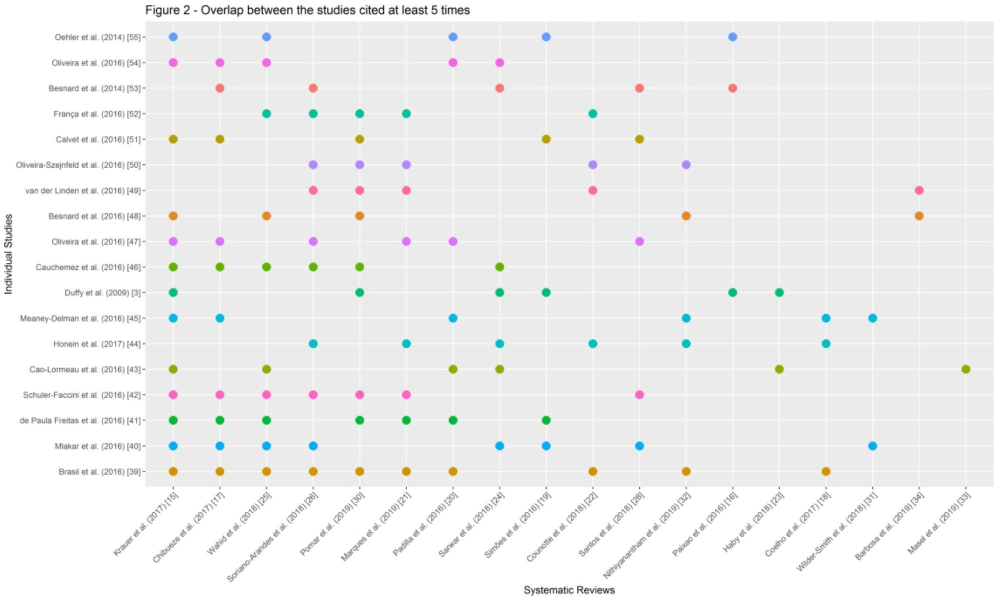

Overlap between studies cited in at least 5 systematic reviews.

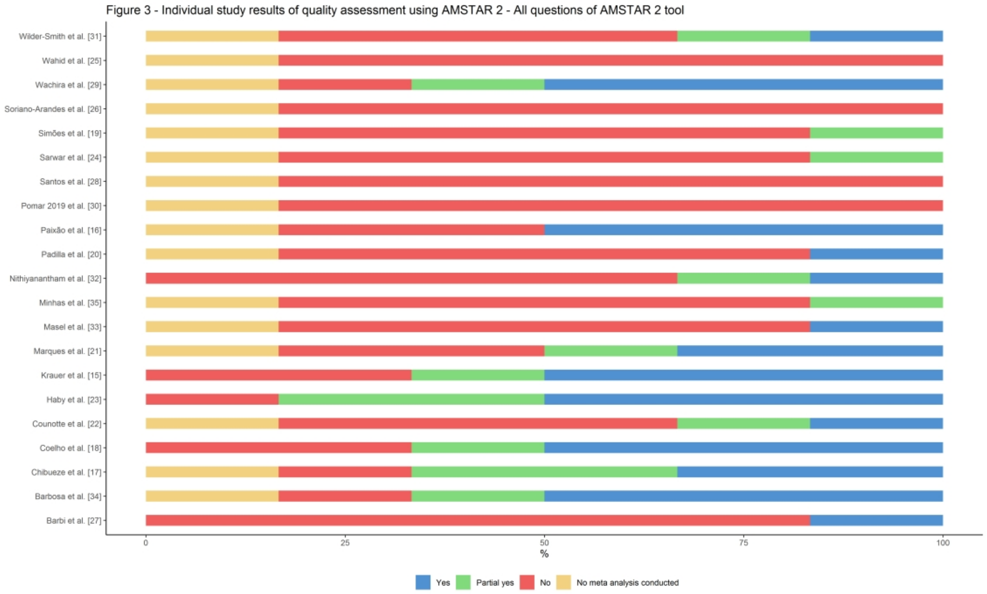

Individual study results of quality assessment using AMSTAR 2 - Result for all questions of AMSTAR 2 tool.

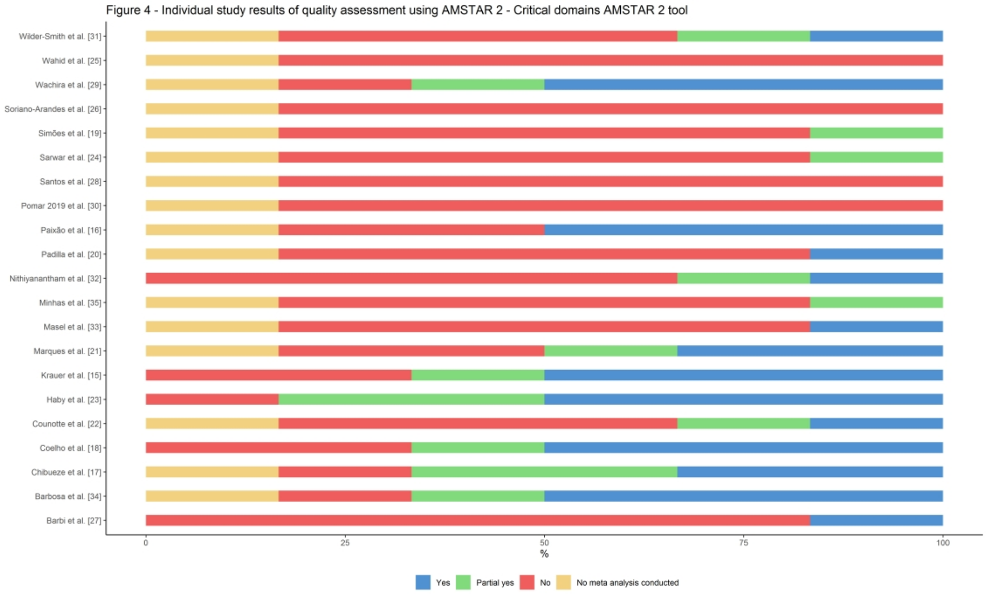

Individual study results of quality assessment using AMSTAR 2 - Critical domains of AMSTAR 2 tool.

Search Strategy

Database: Embase Classic+Embase <1947 to 2018 February 27>

Search Strategy:

1 zika fever/ or zika virus/ or zika virus vaccine/ or zika.mp. (5013)

2 "systematic review"/ or "review"/ (2367967)

3 1 and 2 (569)

Database: Ovid MEDLINE(R) <1946 to February Week 3 2018>

Search Strategy:

1 exp ZIKA VIRUS INFECTION/ or exp ZIKA VIRUS/ or zika.mp. (2287)

2 "review"/ (2215441)

3 1 and 2 (326)

Database: Cochrane

Search Strategy:

1 ZIKA and review (2)

Update – 22/07/2019

Database: LILACS

Search Strategy:

(tw:((tw:(ZIKA VIRUS INFECTION)) OR (tw:(ZIKA VIRUS)) OR (tw:(zika.mp)))) AND (tw:(systematic review)) (729)

Database(s): Ovid MEDLINE(R) 1946 to July Week 2 2019

Search Strategy:

1 exp ZIKA VIRUS INFECTION/ or exp ZIKA VIRUS/ or zika.mp. (4560)

2 "review"/ (2360456)

3 1 and 2 (722)

4 limit 3 to yr="2018-Current" (261)

Database(s): Embase Classic+Embase <1947 to 2019 July 19>

Search Strategy:

1 zika fever/ or zika virus/ or zika virus vaccine/ or zika.mp. (8593)

2 "systematic review"/ or "review"/ (2553024)

3 1 and 2 (1056)

4 limit 3 to yr="2018-Current" (504)

For peer review only

Database: Cochrane

Search Strategy:

1 ZIKA and review (0)

For peer review only

Supplementary file 2 - Table 1. Health outcomes - Congenital Zika syndrome

Authors	Malformations / Congenital abnormalities	Brain abnormalities	Microcephaly	Ocular disorders	Auditory disorder - Rate	Cardiovascular damage
Krauer et al. (2017) [15]		Prevalence: 96% of cases	91% of cases; Prevalence ratio over states with no reported cases of microcephaly= 4.67	Prevalence: 42% (49 cases in 116 mother-infant pairs)	13% (3 cases in 24 mother-infant pairs)	
Paixao et al. (2016) [16]		In 2015, the prevalence of microcephaly in Brazil was 20 cases per 10,000 live births; Zika infection during 9 pregnancies confirmed by CDC resulted in the birth of a neonate with microcephaly.	Rate per 100,000 live births: 121.7 (0.12%) in 2015 in Brazil; Death due microcephaly: 1.3% in suspected microcephaly cases			
Chibueze et al. (2017) [17]		In one observational study of 35 infants with microcephaly, 11 fetuses had intra-uterine brain injury accompanied by stunting of cerebral growth prior to birth.	One observational study provided a trimester-specific modelling estimate risk for microcephaly per 10,000 ZIKV infected pregnant women per trimester of pregnancy: 1st 95 (34 - 191), 2nd 84 (12 - 196), 3rd trimester: 0 - (0 - 251)			
Coelho et al. (2017) [18]	Other organs damage: French Guiana 2% in 250 live births or mother-infant pair. USA: 7%. Not clear if the denominator is the number of live births or mother-infant pair (301 or 498 respectively)		0.3% in live-birth pregnancies; 14.3% - in live-birth pregnancies; Prevalence (cases/all pregnancies): 2.3%. Prevalence (cases/live births): 2.7%. Death due to microcephaly: 8.3%, would be 5.7% in case of new confirmed cases are included.	Two studies reported a prevalence of ocular damage (0.9% and 1%). It is not clear if the denominator is the number of live births (395 and 301, respectively) or the number mother-infant pair (442 and 498, respectively)		French Guiana: Cardiovascular damage equal to 1%. The denominator is unclear if is the number of live births or mother-infant pair (301 and 498 respectively)
Simoes et al. (2016) [19]	Prevalence of CZS: 10 to 20 cases in 100,000 live births; 8.87% of cases with confirmed changes in CNS		The Ministry of Health in Brazil reported an increase in the number of cases of microcephaly close to 20 times that previously reported (aproximately 0.5 cases for each 10,000 live births) which means 10 microcephaly cases per 10,000 births.			
Padilla et al. (2016) [20]	In 72 women with Zika-positive serology during pregnancy in Brazil, 29% had abnormalities detected on fetal ultrasound. Central nervous system abnormalities were noted after Zika infections as late as 27 weeks' gestation, and placental insufficiency was noted with even later gestational ages.		In 2015, the prevalence of microcephaly in Brazil was 20 cases per 10,000 live births; Zika infection during 9 pregnancies confirmed by CDC resulted in the birth of a neonate with microcephaly.			
Marques et al. (2019) [21]		% of neurological malformations: Subcortical-cortical junction calcifications: 92.9%, Basal ganglia calcifications: 57.1%, Periventricular calcifications: 29.5%. Ventriculomegaly/hydrocephaly: 63.1%. Cerebellar abnormalities: 46.2%, 82% (14 of 17 patients). Corpus callosum abnormalities: 47.9%	39.7% in cases of congenital Zika infection. Almost 100% when the infection occurred during the first trimester and decreased when the infection occurred in the second or third trimester	Prevalence: 44.3% in congenital ZIKV infection, 20% in patients with microcephaly, 33% in patients with ventriculomegaly, and 43% in patients with calcification. Bilateral findings: 76.8% of infants with ocular lesions. In eyes of infants with ocular lesions and congenital ZIKV infection: Macular lesions in 50%, Optical nerve abnormalities: 27.78%, Chorioretinal artrophy/scaring: 10.65%, Focal pigment mottling of retina: 6.94%, Microphtalmia: 3.70%, Glaucoma: 2.31%, Cataract: 2.31%, Iris coloboma: 2.31%, Subluxation: 1.39%		
Counotte et al. (2018) [22]	Prevalence of adverse congenital outcomes: 8.97-49.57% in ZIKV positive women. Birth defects: 5.9% in pregnant asymptomatic women and 5.98% in symptomatic pregnant women		RR between ZIKV exposed and unexposed: 4.4-6.6. OR between women with confirmed ZIKV and without evidence of ZIKV infection: 11.0-55.5			
Haby et al. (2018) [23]			Prevalence of asymptomatic ZIKV infection in mothers who gave birth to babies with microcephaly: 0.36			

Authors	Malformations / Congenital abnormalities	Brain abnormalities	Microcephaly	Ocular disorders	Auditory disorder - Rate	Cardiovascular damage
Sarwar et al. (2018) [24]		Prevalence in dead neonates of ZIKV infected mothers: Pachygyria: 14.28%, Arthrogryposis: 14.28%. Morphologic microcephalus changes: 14.28%. Ventriculomegaly / hydrocephaly: 100%. Cerebellar abnormalities: 28.57%	Risk of 1% when infection occurred in the first trimester of pregnancy	In ZIKV infected infants: Retinal impairment: 28%, Impaired optic nerve: 17%, Optic nerve hypoplasia: 8%		
Wahid et al. (2018) [25]	Fetal abnormalities 28.57% in infected pregnant women. Ventricular calcifications or other central nervous system abnormal amniotic fluid volume or cerebral or umbilical artery flow: 16.67%. (CNS) lesions: 16.67%. 80 of the 185 infants, ZIKV-linked microcephaly: 10 (the value of the denominator is not clear) neonates, 5 of 80 or 185 birth defects such as hydranencephaly, holoprosencephaly, clubfeet, and craniosynostosis, 3 of 80 or 185: cataracts, holoprosencephaly, and ventral pons hypoplasia	Prevalence: 28% (including microcephaly) in newborns of mothers infected with ZIKV	Risk of microcephaly: 0-30%. Relative Risk 100–1,000 (assuming 10% exposure) or 20–200 (assuming 50% exposure) compared to background risk of microcephaly. Prevalence: 50.47% among definite or probable ZIKV cases. Higher risk of microcephaly in pregnant women infected during first trimester. Estimated risk of microcephaly: 0.95% in women infected in the first trimester	In infants with microcephaly: ophthalmoscopic alterations in 50% (not clear if ZIKV-related infection) . Ocular findings 34.5-58.62% of ZIKV linked microcephalic infants		
Soriano-Arandes et al. (2018) [26]	Birth defects: 6% in asymptomatic and symptomatic pregnant women. From 1 study: Fetal adverse outcomes in women infected with ZIKV: 55% in the first term of pregnancy, 29% in the third trimester. In infants with CZS: Dimples: 30.1%, Distal hand/finger contractures: 20.5%, feet malposition: 15.7%, generalized arthrogryposis: 9.6%, birth defects in women with recent ZIKV infection: 6%	Prevalence: Microcephaly in 86.7% and craniofacial disproportion in 95.8% of infants with probable CZS	In infected women in the first trimester: Risk of 0.95% in a population with an estimated rate of ZIKV infection of 66%; Prevalence of 55% in Rio de Janeiro. infection in the 3rd trimester: Prevalence: 29% (Rio de Janeiro). In a series of 13 infants with congenital ZIKV infection and progressive microcephaly, more than half of the mothers did not report any symptoms prior to delivery.		In a study of 70 children with microcephaly and laboratory diagnosys of congenital ZIKV infection, 5 (7%) had sensorineural hearing loss.	One study: congenital heart disease was described in 14 of a series of 103 cases (13.6%) in children with CZS.
Santos et al. (2018) [28]		Intracranial calcifitcation: 23 of 23 childen. Frontal lobe: 69% - 78%. Parietal lobe: 83% - 87%. Corticomedullary junction: 53% - 86%. Thalamus: 39% - 43%. Punctate calcification: 72% - 100%. Distributed in the band format: 56% - 75%. Reduction in the constitution of gyri of the severe cerebral cortex: 0.78. Cerebellar hypoplasia: 0.74. Involving only one cerebellar hemisphere : 13%. Brainstem globally hypoplastic: 8.7%. Abnormal hypodensity of the white matter: 1. Diffuse involvement of all the cerebral lobes: 0.87. Basal ganglia calcification: 57% - 65%				
Pomar et al. (2019) [30]	CZS: 4-9% of pregnancies of women infected by ZIKV. Malformations of cortical development: 79-82% of CZS cases. Intraventicular synechiae and periventricular cystic degeneration: 58% of CZS cases. Malformations of the corpus callosum: 71-100%. Vermian hypoplasia: 42% of CZS cases. 21% to 82%. Swallowing disorders and hydramnios: 25%. Partial immobilization or arthrogryposes: 10-25%. Motor abnormalities : 77.3-100% of CZS cases. Adverse outcomes - No signs/complications: 45% of proven infected fetuses/newborn. Adverse outcomes - Mild / moderate signs: 20% of proven infected fetuses/newborn. Adverse outcomes - Severe complications: 21% of proven infected fetuses/newborn. Risk of neurodevelopmental abnormality: 9% of infants born from infected mothers	Brain volume loss: 92%. Ventriculomegaly in CZS: 63.1-92%. Calcifications in CZS: 71-92%	Prevalence of microcephaly in CZS: 33.3-64%	Eye abnormalities: 25% in infants with CZS		

Authors	Malformations / Congenital abnormalities	Brain abnormalities	Microcephaly	Ocular disorders	Auditory disorder - Rate	Cardiovascular damage
**Wilder-Smith et al. (2018) [31]**	From infected pregnant travelers: Fetuses or infants with birth defects: 6% for asymptomatic women and 6% for symptomatic women with evidence of possible recent ZIKV infection. Zika virus-associated birth defects in infants with ZIKV infection: 10% in completed pregnancies with reported outcomes; 5% in infants with possible ZIKV-associated birth defects from women with confirmed or probably ZIKV infection) (5% among symptomatic and 4% among asymptomatic women). Among 1,508 pregnancies with lab-confirmed ZIKV (5% among symptomatic and 7% among asymptomatic woman). Adverse fetal outcomes: 7% in pregnant women with symptomatic ZIKV infection. Adverse outcomes: 3 of 4 ZIKV infected pregnant women.					
**Nithiyanantham et al. (2019) [32]**	Prevalence of joint abnormalities: 13.2% in infants of ZIKV-infected mothers	In infants of ZIKV-infected mothers: Ventriculomegaly / hydrocephaly: 21.8% (95% CI,15.2-28.4); Brain calcifications: 42.6% (95% CI, 30.8-54.4)	Prevalence of 3.9% in infants of ZIKV-infected mothers	Prevalence: 4.2% in infants of ZIKV-infected mothers		
**Masel et al. (2019) [33]**	No association of prior exposure to DENV and fetal imaging abnormalities					
**Barbosa et al. (2019) [34]**	Microcephaly or neurologic changes: 50.10% on 962 fetus or children studied				Altered OAE varied from 0% to 75%, while altered a-ABR varied from 0% to 29.9%. Among patients who underwent OAE assessments (n=244), 18,4% presented alterations while 25% of microcephaly cases dislpayed alterations. Among the 448 patients who reportedly underwent the first a-ABR test, 15.2% presented alterations. Among three studies that included 102 children with laboratory confirmation of congenital ZIKV infection, 18 (17.6%) had hearing alterations, five in the ABR and 13 in the HINE.	
**Minhas et al. (2017) [35]**						Cohort with 9 adults positive for ZIKV and no previous cardiac history. 8 of the cases had arrhythmicas and 6 presented heart failure. Of the 8 arrhythmias, 3 were acute atrial fibrillation (two paroxysmal, one persistent), 2 were non-sustained atrial tachycardia, and 2 were ventricular arrhythmias. 5 of the 6 heart failure patients had a low ejection fraction (EF), and one had preserved EF with pre-eclampsia and moderate to severe pericardial effusion.

Supplementary file 2 - Table 2. Health outcomes - Neurological

Authors	Neurological complications	Epilepsy	Sleep characteristics	GBS
**Krauer et al. (2017) [15]**				74-84% symptomatic ZIKV in GBS cases; ZIKV laboratory-confirmed in GBS cases investigated: 100%
**Paixao et al. (2016) [16]**	French Polynesia outbreak: Amongg patients that visited health care facilities with Zika-like symptoms, 2.3 per 1,000 had neurological complications			In Bahia, Brazil, GBS was diagnosed in 1 of every 1,000 reported ZIKV cases. French Polynesia outbreak: Among patients that visited health care facilities with Zika-like symptoms, 1.3/1,000 ZIKV infections had GBS. ZIKV symptomatic cases when confirmed Among 42 GBS cases, 36% required intensive care and 21% required mechanical ventilation; El salvador: Prevalence of 35% (84 GBS cases in 240 ZIKV infections)
**Simoes et al. (2016) [19]**				In the primary databases consulted, there is only one case report occurred in French Polynesia in which GBS was diagnosed in a patient infected with Zika virus.
**Padilla et al. (2016) [20]**				An analysis of 42 patients who developed GBS during the French Polynesia outbreak estimates the incidence of the disease to be 0.24 per 1000 Zika virus infections. 88% of these patients reported symptoms and 93% of patients showed evidence of recent disease with ZIKV confirmed by the presence of IgM antibodies. Of these patients, 38% required admission to an intensive care unit and 29% required mechanical ventilation.
**Marques et al. (2019) [21]**		Prevalence of epilepsy: 42.2-67% in children with congenital ZIKV. Infantile spasms: 72%, 21.6%. Generalized: 11.8%. Partial: 8.9%. Described as brief jerking spells of flexion and/or extension movements that lasted a few seconds : 21.57%. Focal motor seizures: 21%. Tonic seizures: 4%. Myoclonic seizures: 2%. Myoclonic seizures: 1%.	34.1% (30 in 88 congenital ZIKV-infected children) were defined as poor sleepres and 24% (21 in 88) slept less than 9 hours	

For peer review only - http://bmjopen.bmj.com/site/about/guidelines.xhtml

Authors	Neurological complications	Epilepsy	Sleep characteristics	GBS
**Counotte et al. (2018) [22]**				Prevalence ratio during the ZIKV transmission over pre-outbreak period: 2.0-9.8.
**Haby et al. (2018) [23]**				Prevalence of asymptomatic ZIKV infection in patients with GBS: 0.12
**Wahid et al. (2018) [25]**	A recent study presented neurological disorders in 12 of 16 patients co-infected with ZIKV, chikungunya virus, and dengue virus in Guayaquil, Ecuador. One patients experienced CNS vasculitis, three had GBS whereas, and six patients were diagnosed with meningitis or encephalitis.			About 43% of GBS patients were found to be positive for ZIKV. Another study confirmed ZIKV-linked GBS in 1 of 3 patients.
**Barbi et al. (2018) [27]**				Meta-analysis: 1513 GBS cases in 164,651 ZIKV-infected individuals (0.92%). Estimative the prevalence of GBS to be 1.23% (CI: 95% 1.17%-1.29%) of all ZIKV infection cases in adults. 16 in 38 GBS cases (42%) needed intensive care unit hospitalization (French Polynesia)
**Wachira et al. (2018) [29]**				OR: 59.7 (CI: 95% 10.4 - ∞); Other study: no statistical significance between ZIKV and GBS
**Pomar et al. (2019) [30]**		9-95.5% in congenital ZIKV infection		Prevalence of 1.23% (95% CI, 1.17%-1.29%) in general ZIKV infected-population)
**Wilder-Smith et al. (2018) [31]**				2.15% (2 cases in 93 ZIKV cases recorded in Geosentinel sites)
**Masel et al. (2019) [33]**	No association of prior exposure to DENV and clinical neurological assessment of fetus			No statistically significant difference in patients with GBS with or without prior DENV exposure. No statistical difference in prior DENV exposed patients with or without GBS after ZIKV infection.

For peer review only - http://bmjopen.bmj.com/site/about/guidelines.xhtml

Supplementary file 2 - Table 3. Health outcomes – Adverse outcomes

Authors	Death due ZIKV infection	Abortion due to ZIKA / fetal death / perinatal death / neonatal death	Intrauterine growth restrictions - Rate within mother-infant pairs	Abnormal amniotic fluid	Adverse birth outcomes
**Krauer et al. (2017) [15]**		Prevalence in all pregnancy outcomes: Miscarriage 2.5%; intrauterine death or stillbirth 1.1%; termination of pregnancy 5.4%; Neonatal death: 3.2%	28.57% of cases	Rate: 18% of infected pregnant women	
**Paixão et al. (2016) [16]**	In Brazil, 2 deaths of adults were attribuited to Zika and 7 are under investigation by the Ministry of Health; El Salvador (240 ZIKV cases, 2 deaths)				
**Chibueze et al. (2017) [17]**					
**Coelho et al. (2017) [18]**		Miscarriages and perinatal deaths: USA (22% - 2 deaths in 9 ZIKV infected pregnant women), Brazil (6.7% - 9 deaths in 135 ZIKV infected pregnant women), Puerto Rico (3% - 2 deaths in 67 ZIKV infected pregnant women), USA (10.6% - 47 deaths in 442 ZIKV infected pregnant women), French Guiana (4% - 20 deaths in 498 ZIKV infected pregnant women).			
**Simões et al. (2016) [19]**		In Brazil, 1.79% (91/5,079) of microcephaly reported cases, progressed to miscarriage or postpartum death. According to the classification, 64.8% (59/91) remained under investigation; 838% (8/91) were investigated and discarded, and 26.4% (24/91) were investigated and confirmed for microcephaly and/or changes in the CNS.			
**Padilla et al. (2016) [20]**		In 72 women with Zika-positive serology during pregnancy in Brazil, the fetal death rate was 4.8%; Zika infection during 9 pregnancies confirmed by CDC resulted in outcomes of 2 spontaneous abortions and 2 elective abortions.			
**Wahid et al. (2018) [25]**			One study with 88 pregnant women of which 72 were positive for ZIKV and ultrasonography was performed in 42: in utero growth restriction with or without microcephaly (5/42).		
**Pomar et al. (2019) [30]**		14% of proven infected fetuses/newborn	Prevalence of IUGR in CZS: 14%		
**Masel et al. (2019) [33]**		No association of prior exposure to DENV and fetal loss			Occured in 46.4% of those ZIKV infected participants

For peer review only

Supplementary file 3 - Table 1.1. Summary of AMSTAR 2 rating

AMSTAR 2	Krauer et al. [15]	Paixão et al. [16]	Chibueze et al. [17]	Coelho et al. [18]	Simões et al. [19]	Padilla et al. [20]	Marques et al. [21]
1	Yes	Yes	Yes	Yes	Yes	No	Yes
2	No	No	Partial yes	No	No	No	Yes
3	Yes	Yes	Yes	Yes	Yes	Yes	Yes
4	Partial yes	Yes	Partial yes	Partial yes	Partial yes	Yes	Partial yes
5	Yes	Yes	Yes	Yes	Yes	Yes	Yes
6	Yes	No	Yes	Yes	No	No	No
7	Yes	Yes	Yes	Yes	No	No	No
8	Partial yes	Partial yes	Yes	Partial yes	No	No	No
9	No	No	No	No	No	No	No
10	Yes	No	Yes	Yes	No	No	Yes
11	No	No	No	No	No	No	No
12	Yes	No MA conducted	No MA conducted	Yes	No MA conducted	No MA conducted	No MA conducted
13	No	No MA conducted	No MA conducted	No	No MA conducted	No MA conducted	No MA conducted
14	No	No	No	No	No	No	No
15	Yes	Yes	No	Yes	No	No	No
16	No	No MA conducted	No MA conducted	No	No MA conducted	No MA conducted	No MA conducted

*MA - Meta-analysis

Questions 2,4,7,9,12 and 14, highlighted, are those of critical domains.

1 - Did the research questions and inclusion criteria for the review include the components of PICO?

2 - Did the report of the review contain an explicit statement that the review methods were established prior to the conduct of the review and did the report justify any significant deviations from the protocol?

3 - Did the review authors explain their selection of the study designs for inclusion in the review?

4 - Did the review authors use a comprehensive literature search strategy?

5 - Did the review authors perform study selection in duplicate?

6 - Did the review authors perform data extraction in duplicate?

7 - Did the review authors provide a list of excluded studies and justify the exclusions?

8 - Did the review authors describe the included studies in adequate detail?

9 - Did the review authors use a satisfactory technique for assessing the risk of bias (RoB) in individual studies that were included in the review?

10 - Did the review authors report on the sources of funding for the studies included in the review?

11 - If meta-analysis was performed did the review authors use appropriate methods for statistical combination of results?

12 - If meta-analysis was performed, did the review authors assess the potential impact of RoB in individual studies on the results of the meta-analysis or other evidence synthesis?

13 - Did the review authors account for RoB in individual studies when interpreting/ discussing the results of the review?

14 - Did the review authors provide a satisfactory explanation for, and discussion of, any heterogeneity observed in the results of the review?

15 - If they performed quantitative synthesis did the review authors carry out an adequate investigation of publication bias (small study bias) and discuss its likely impact on the results of the review?

16 - Did the review authors report any potential sources of conflict of interest, including any funding they received for conducting the review?

For peer review only

Supplementary file 3 - Table 1.2. Summary of AMSTAR 2 rating

AMSTAR 2	Counotte et al. [22]	Haby et al. [23]	Sarwar et al. [24]	Wahid et al. [25]	Soriano-Arandes et al. [26]	Barbi et al. [27]	Santos et al. [28]
1	Yes	Yes	Yes	Yes	Yes	Yes	Yes
2	Yes	Partial yes	No	No	No	No	No
3	No	Yes	No	No	No	No	No
4	Partial yes	Partial yes	Partial yes	No	No	No	No
5	Yes	No	No	Yes	No	No	No
6	Yes	No	No	No	No	Yes	No
7	No	Yes	No	No	No	No	No
8	Yes	Yes	No	Yes	Yes	Yes	No
9	No	No MA conducted	No MA conducted	No MA conducted	No MA conducted	No MA conducted	No
10	No	Yes	No	No	No	Yes	No
11	No	No	No	No	Yes	No	No
12	No MA conducted	Yes	No MA conducted	No MA conducted	No MA conducted	No	No MA conducted
13	No MA conducted	No	No MA conducted	No MA conducted	No MA conducted	No	No MA conducted
14	No	Yes	No	No	No	No	Yes
15	No	Yes	No	No	No	No	No
16	No MA conducted	Yes	No MA conducted	No MA conducted	No MA conducted	No	No MA conducted

Questions 2,4,7,9,12 and 14, highlighted, are those of critical domains.
1 - Did the research questions and inclusion criteria for the review include the components of PICO?
2 - Did the report of the review contain an explicit statement that the review methods were established prior to the conduct of the review and did the report justify any significant deviations from the protocol?
3 - Did the review authors explain their selection of the study designs for inclusion in the review?
4 - Did the review authors use a comprehensive literature search strategy?
5 - Did the review authors perform study selection in duplicate?
6 - Did the review authors perform data extraction in duplicate?
7 - Did the review authors provide a list of excluded studies and justify the exclusions?
8 - Did the review authors describe the included studies in adequate detail?
9 - Did the review authors use a satisfactory technique for assessing the risk of bias (RoB) in individual studies that were included in the review?
10 - Did the review authors report on the sources of funding for the studies included in the review?
11 - If meta-analysis was performed did the review authors use appropriate methods for statistical combination of results?
12 - If meta-analysis was performed, did the review authors assess the potential impact of RoB in individual studies on the results of the meta-analysis or other evidence synthesis?
13 - Did the review authors account for RoB in individual studies when interpreting/ discussing the results of the review?
14 - Did the review authors provide a satisfactory explanation for, and discussion of, any heterogeneity observed in the results of the review?
15 - If they performed quantitative synthesis did the review authors carry out an adequate investigation of publication bias (small study bias) and discuss its likely impact on the results of the review?
16 - Did the review authors report any potential sources of conflict of interest, including any funding they received for conducting the review?

For peer review only

Supplementary file 3 - Table 1.3. Summary of AMSTAR 2 rating

AMSTAR 2	Wachira et al. [29]	Pomar et al. [30]	Wilder-Smith et al. [31]	Nithiyanantham et al. [32]	Masel et al. [33]	Barbosa et al. [34]	Minhas et al. [35]
**1**	Yes	Yes	Yes	Yes	Yes	Yes	Yes
**2**	Yes	No	No	No	No	Yes	No
**3**	Yes	No	Yes	Yes	Yes	Yes	Yes
**4**	Partial yes	No	Partial yes	Partial yes	Yes	Partial yes	Partial yes
**5**	Yes	Yes	No	Yes	Yes	Yes	Yes
**6**	Yes	Yes	No	No	Yes	Yes	Yes
**7**	No	No	No	No	No	No	No
**8**	Yes	No	Partial yes	Yes	Yes	Yes	Yes
**9**	No	No	No	No	No	Yes	No
**10**	Yes	No	No	No	No	No	No
**11**	No	No MA conducted	No MA conducted	No	No MA conducted	No MA conducted	No MA conducted
**12**	No MA conducted	No MA conducted	No MA conducted	No	No MA conducted	No MA conducted	No MA conducted
**13**	No MA conducted	No	No	No	No	No	No
**14**	Yes	No	Yes	Yes	No	Yes	No
**15**	Yes	No MA conducted	No MA conducted	Yes	No MA conducted	No MA conducted	No MA conducted
**16**	No MA conducted	Yes	Yes	Yes	Yes	Yes	Yes

Questions 2,4,7,9,12 and 14, highlighted, are those of critical domains.
1 - Did the research questions and inclusion criteria for the review include the components of PICO?
2 - Did the report of the review contain an explicit statement that the review methods were established prior to the conduct of the review and did the report justify any significant deviations from the protocol?
3 - Did the review authors explain their selection of the study designs for inclusion in the review?
4 - Did the review authors use a comprehensive literature search strategy?
5 - Did the review authors perform study selection in duplicate?
6 - Did the review authors perform data extraction in duplicate?
7 - Did the review authors provide a list of excluded studies and justify the exclusions?
8 - Did the review authors describe the included studies in adequate detail?
9 - Did the review authors use a satisfactory technique for assessing the risk of bias (RoB) in individual studies that were included in the review?
10 - Did the review authors report on the sources of funding for the studies included in the review?
11 - If meta-analysis was performed did the review authors use appropriate methods for statistical combination of results?
12 - If meta-analysis was performed, did the review authors assess the potential impact of RoB in individual studies on the results of the meta-analysis or other evidence synthesis?
13 - Did the review authors account for RoB in individual studies when interpreting/ discussing the results of the review?
14 - Did the review authors provide a satisfactory explanation for, and discussion of, any heterogeneity observed in the results of the review?
15 - If they performed quantitative synthesis did the review authors carry out an adequate investigation of publication bias (small study bias) and discuss its likely impact on the results of the review?
16 - Did the review authors report any potential sources of conflict of interest, including any funding they received for conducting the review?

For peer review only

PRISMA 2009 Checklist

Section/topic	#	Checklist item	Reported on page #
**TITLE**			
Title	1	Identify the report as a systematic review, meta-analysis, or both.	1
**ABSTRACT**			
Structured summary	2	Provide a structured summary including, as applicable: background; objectives; data sources; study eligibility criteria, participants, and interventions; study appraisal and synthesis methods; results; limitations; conclusions and implications of key findings; systematic review registration number.	2
**INTRODUCTION**			
Rationale	3	Describe the rationale for the review in the context of what is already known.	3
Objectives	4	Provide an explicit statement of questions being addressed with reference to participants, interventions, comparisons, outcomes, and study design (PICOS).	3
**METHODS**			
Protocol and registration	5	Indicate if a review protocol exists, if and where it can be accessed (e.g., Web address), and, if available, provide registration information including registration number.	3,4
Eligibility criteria	6	Specify study characteristics (e.g., PICOS, length of follow-up) and report characteristics (e.g., years considered, language, publication status) used as criteria for eligibility, giving rationale.	3,4
Information sources	7	Describe all information sources (e.g., databases with dates of coverage, contact with study authors to identify additional studies) in the search and date last searched.	3,4
Search	8	Present full electronic search strategy for at least one database, including any limits used, such that it could be repeated.	3
Study selection	9	State the process for selecting studies (i.e., screening, eligibility, included in systematic review, and, if applicable, included in the meta-analysis).	3,4
Data collection process	10	Describe method of data extraction from reports (e.g., piloted forms, independently, in duplicate) and any processes for obtaining and confirming data from investigators.	3,4
Data items	11	List and define all variables for which data were sought (e.g., PICOS, funding sources) and any assumptions and simplifications made.	
Risk of bias in individual studies	12	Describe methods used for assessing risk of bias of individual studies (including specification of whether this was done at the study or outcome level), and how this information is to be used in any data synthesis.	4
Summary measures	13	State the principal summary measures (e.g., risk ratio, difference in means).	5-6
Synthesis of results	14	Describe the methods of handling data and combining results of studies, if done, including measures of consistency (e.g., I^2) for each meta-analysis.	

For peer review only

PRISMA 2009 Checklist

Section/topic	#	Checklist item	Reported on page #
Risk of bias across studies	15	Specify any assessment of risk of bias that may affect the cumulative evidence (e.g., publication bias, selective reporting within studies).	
Additional analyses	16	Describe methods of additional analyses (e.g., sensitivity or subgroup analyses, meta-regression), if done, indicating which were pre-specified.	
**RESULTS**			
Study selection	17	Give numbers of studies screened, assessed for eligibility, and included in the review, with reasons for exclusions at each stage, ideally with a flow diagram.	4
Study characteristics	18	For each study, present characteristics for which data were extracted (e.g., study size, PICOS, follow-up period) and provide the citations.	5
Risk of bias within studies	19	Present data on risk of bias of each study and, if available, any outcome level assessment (see item 12).	5
Results of individual studies	20	For all outcomes considered (benefits or harms), present, for each study: (a) simple summary data for each intervention group (b) effect estimates and confidence intervals, ideally with a forest plot.	
Synthesis of results	21	Present results of each meta-analysis done, including confidence intervals and measures of consistency.	
Risk of bias across studies	22	Present results of any assessment of risk of bias across studies (see Item 15).	
Additional analysis	23	Give results of additional analyses, if done (e.g., sensitivity or subgroup analyses, meta-regression [see Item 16]).	
**DISCUSSION**			
Summary of evidence	24	Summarize the main findings including the strength of evidence for each main outcome; consider their relevance to key groups (e.g., healthcare providers, users, and policy makers).	6-8
Limitations	25	Discuss limitations at study and outcome level (e.g., risk of bias), and at review-level (e.g., incomplete retrieval of identified research, reporting bias).	8
Conclusions	26	Provide a general interpretation of the results in the context of other evidence, and implications for future research.	9
**FUNDING**			
Funding	27	Describe sources of funding for the systematic review and other support (e.g., supply of data); role of funders for the systematic review.	9

From: Moher D, Liberati A, Tetzlaff J, Altman DG, The PRISMA Group (2009). Preferred Reporting Items for Systematic Reviews and Meta-Analyses: The PRISMA Statement. PLoS Med 6(7): e1000097. doi:10.1371/journal.pmed1000097

For more information, visit: **www.prisma-statement.org**.

For peer review only - http://bmjopen.bmj.com/site/about/guidelines.xhtml

BMJ Open

Health outcomes associated with Zika virus infection in humans: a systematic review of systematic reviews

Journal:	*BMJ Open*
Manuscript ID	bmjopen-2019-032275.R2
Article Type:	Original research
Date Submitted by the Author:	18-Sep-2019
Complete List of Authors:	Ximenes, Raphael; University Health Network, Toronto Health Economics and Technology Assessment (THETA) Collaborative, University Health Network<br>Ramsay, Lauren; University Health Network, Toronto Health Economics and Technology Assessment (THETA) Collaborative, University Health Network; University of Toronto, Institute of Health Policy, Management and Evaluation, University of Toronto<br>Miranda, Rafael; University Health Network, University Health Network, Toronto Health Economics and Technology Assessment (THETA) Collaborative, University Health Network<br>Morris, Shaun; Hospital fo sick children, Division of Infectious Diseases and Centre for Global Child Health, Hospital for Sick Children; University of Toronto Faculty of Medicine, Department of Pediatrics, Faculty of Medicine, University of Toronto<br>Murphy, Kellie; Mount Sinai Hospital<br>Sander, Beate; University Health Network, Toronto Health Economics and Technology Assessment (THETA) Collaborative, University Health Network; University of Toronto, Institute of Health Policy, Management and Evaluation, Management and Evaluation, University of Toronto
**Primary Subject Heading**:	Infectious diseases
Secondary Subject Heading:	Global health, Epidemiology, Obstetrics and gynaecology, Paediatrics, Neurology
Keywords:	Zika Virus infection, Epidemiology < INFECTIOUS DISEASES, Congenital Zika Syndrome, NEUROLOGY, Guillain-Barré syndrome, Microcephaly

SCHOLARONE™
Manuscripts

Health outcomes associated with Zika virus infection in humans: a systematic review of systematic reviews

Raphael Ximenes[1], Lauren C. Ramsay[1,2], Rafael N. Miranda[1], Shaun K. Morris[3,4], Kellie E. Murphy[5], RADAM-LAC Research Team, Beate Sander[1,2,6,7]

[1] Toronto Health Economics and Technology Assessment (THETA) Collaborative, University Health Network, Toronto, ON, Canada, raphael.ximenes@theta.utoronto.ca.

[2] Institute of Health Policy, Management and Evaluation, University of Toronto, Toronto, ON, Canada.

[3] Division of Infectious Diseases and Centre for Global Child Health, Hospital for Sick Children, Toronto, ON, Canada.

[4] Department of Pediatrics, Faculty of Medicine, University of Toronto, Toronto, ON, Canada.

[5] Mount Sinai Hospital, Toronto, ON, Canada.

[6] Public Health Ontario, Toronto, ON, Canada.

[7] ICES, Toronto, ON, Canada.

Correspondence to

Raphael Ximenes: raphael.ximenes@theta.utoronto.ca

RADAM-LAC Research Team members are Beate Sander, Camila Gonzalez, Manisha Kulkarni, Marcos Miretti, Mauricio Espinel, Jianhong Wu and Varsovia Cevallos.

For peer review only

Abstract

Objective: With the emergence of Zika virus (ZIKV) disease in Central and South America in the mid-2010s and recognition of the teratogenic effects of congenital exposure to ZIKV, there has been a substantial increase in new research published on ZIKV. Our objective is to synthesize the literature on health outcomes associated with ZIKV infection in humans.

Methods: We conducted a systematic review (SR) of SRs following PRISMA (Preferred Reporting Items for Systematic Reviews and Meta-Analyses) guidelines. We searched MEDLINE, Embase, Cochrane and LILACS databases from inception to July 22, 2019, and included SRs that reported ZIKV associated health outcomes. Three independent reviewers selected eligible studies, extracted data and assessed the quality of included SRs using the A MeaSurement Tool to Assess Systematic Reviews 2 (AMSTAR 2) tool. Conflicts were resolved by consensus or consultation with a third reviewer.

Results: The search yielded 1,382 unique articles, of which 21 SRs met our inclusion criteria. The 21 SRs ranged from descriptive to quantitative data synthesis, including four meta-analysis. The most commonly reported ZIKV-associated manifestations and health outcomes were microcephaly, congenital abnormalities, brain abnormalities, neonatal death, and Guillain-Barré syndrome. The included reviews were highly heterogeneous. The overall quality of the SRs was critically low with all studies having more than one critical weakness.

Conclusion: The evolving nature of the literature on ZIKV-associated health outcomes, together with the critically low quality of existing SRs, demonstrate the need for high-quality SRs to guide patient care and inform policy decision making.

Strengths and limitations:

- Lack of SRs on ZIKV in the literature
- Lack of information about the risks of severe outcomes related to ZIKV infection or the presence of specific outcomes
- Broad search strategy
- Without restrictions by language or publication type
- To our knowledge, this is the first SR of SRs about health outcomes associated with ZIKV infection in humans

For peer review only

Introduction

Zika Virus (ZIKV) was first discovered in 1947 in rhesus monkeys in Uganda (1). It is an arbovirus in the flavivirus family and typically causes mild illness in humans characterized by fever and rash. There were reports of sporadic cases of ZIKV infection in humans over the years in Asia and Africa (2), with the first large documented outbreak taking place in Yap, a Micronesian island, in 2007 (3). Since then, there have been reported outbreaks in French Polynesia (in 2013-2014), and most recently in South and Central America and the Caribbean (4). With the emergence of ZIKV in Brazil, there were over 800,000 estimated cases of ZIKV infection reported by countries and territories in the Americas by January 2018 (5). By March 2017, according to the latest World Health Organization (WHO) global situation report on Zika, 84 countries, territories or subnational areas had evidence of vector-borne ZIKV transmission (6). According to the CDC, until May 2019, there were 89 areas with current or past transmission, but no current outbreak of ZIKV (7).

Our understanding of Zika-associated clinical outcomes has evolved over time. Before human pathogenesis was understood, cellular level damage was apparent in animal studies in the 1950s (8). The first study in humans to suggest an association between ZIKV and human disease was a case-control study during an outbreak in French Polynesia between 2013 and 2014, suggesting an association with Guillain-Barre Syndrome (GBS). (9). However, the link between ZIKV in pregnant women and microcephaly in infants was only evident in the 2015-2016 outbreak in South America (10). With the spread of ZIKV to new regions of the world and the extent of the outbreak in South and Central American and Caribbean countries, a substantial body of new research has been published in recent years about Zika.

A bibliometric analysis of ZIKV research that indexed in Web of Science found a significant increase in the number of studies being published beginning in 2015 (n=38 publications) to 2017 (n=1,962 publications) (11). Summarizing the large body of literature on outcomes associated with ZIKV infection is timely and needed.

The purpose of this systematic review (SR) of systematic reviews was to synthesize the currently known health outcomes associated with ZIKV infection in humans.

Methods

Search strategy and selection criteria

We searched MEDLINE, Embase, Cochrane and LILACS databases from inception to July 22, 2019. Our search strategy across all databases included concepts related to “Zika” and “systematic review” (complete search strategy found in Supplementary File 1). Our search strategy was not restricted by language or publication type. Three reviewers (RX, first reviewer; LR and RM second reviewers) independently screened titles, abstracts, and relevant full text of identified articles.

For peer review only

The inclusion criteria were defined as SRs that reported health outcomes of ZIKV infection in humans, i.e. clinical presentation and sequelae of ZIKV infection in humans. We excluded studies that only reported symptoms (e.g., rash, fever) of ZIKV infection, diagnostic techniques, mosquito control, therapeutic regimes, vaccine and trial but not outcomes (e.g., GBS, Congenital Zika Syndrome). We followed the Preferred Reporting Items for Systematic Reviews and Meta-Analyses (PRISMA) guidelines for reporting results (12).

The data extraction was performed in duplicate by the reviewers. The SR methods were established prior to the conduct of the SR and the protocol for the current SR was registered with PROSPERO (CRD42018091087) and there were no deviations from the protocol, except for adding the LILACS database to the search.

Patient and Public Involvement

No patient involved.

Quality appraisal

We used the A MeaSurement Tool to Assess systematic Reviews 2 (AMSTAR 2) tool to critically appraise the included SRs (13). AMSTAR 2 is not intended to generate an overall score, but rather to assist in the identification of high-quality SRs. Three reviewers (RX, first reviewer; LR and RM, second reviewers) independently evaluated the quality of each study based on weaknesses in critical domains as defined by the AMSTAR 2 tool. Studies were rated based on the overall confidence in the results of the SR and defined as either high (zero or one non-critical weakness), moderate (more than one non-critical weakness), low (one critical flaw with or without non-critical weaknesses) or critically low (more than one critical flaw with or without non-critical weaknesses) (14). Critical domains included protocol registration, adequacy of the literature search, justification for excluding studies, risk of bias from individual studies included in the SR, appropriateness of meta-analytical methods, consideration of risk of bias when interpreting results, and assessment of publication bias (14). Any disagreements between the two reviewers were resolved by consensus.

Data analysis

Three reviewers (RX, first reviewer; LR and RM, second reviewers) extracted the data using a structured electronic data extraction form, extracting study characteristics, and measures of effect for outcomes resulting from ZIKV infection. Included studies were summarized narratively, and health outcomes were reported where possible.

For peer review only

Results

We identified 1,382 unique articles from the database searches (Figure 1). After screening titles and abstracts, we selected 85 for full text screening. Of these, twenty-one met our inclusion criteria (15–35). The main reasons for exclusion at the full text stage were articles not being SRs (but rather overviews or literature reviews) and studies only reported symptoms but not outcomes.

Study characteristics are summarized in Table 1. The included SRs were published between February 2016 and May 2019. The types of studies eligible for inclusion in the SRs varied across studies; four SR did not include any information on the included studies (21,24,28,30), all other SRs included observational studies (one limited to only cohort studies (18)), and the majority (71%; n=15) included case reports and case series. Three SRs considered evidence from modelling studies, animal experiments, and in vitro experiments (15,33,35). Another did not limit to reports of primary data and included SRs, narrative reviews, and news articles (20).

The majority of studies included in the SRs were conducted in Brazil, the United States (US), French Polynesia and Colombia.

Summary of included SRs and outcomes

Of the 21 included SRs, the most commonly reported outcome was microcephaly, reported in 14 SRs (15–26,30,32), 12 SRs reported on GBS (15,16,19,20,22,23,25,27,29–31,33), 11 SRs reported on malformations or congenital abnormalities (18–20,22,26,30–34), 9 reported on brain (15,17,21,24–26,28,30,32), 7 SRs reported on ocular disorders (15,18,21,24,25,30,32), and 6 SRs on termination of pregnancy, fetal death and perinatal death (15,18–20,30,33). Three SRs or fewer reported on auditory disorder (15,26,34), cardiovascular damage (18,26,35), neurological complications (16,25,33), intrauterine growth restrictions (15,25), abnormal amniotic fluid (15), epilepsy (21), and death due Zika infection (16).

Seven SRs focused on pregnant women (17–20,24,26,28) and 5 SRs included the general population (15,16,22,23,29), while newborns, neonates, perinatal, early birth or infants were included in 5 five SRs (18,19,21,25,26). One SR focused in travelers returning to the US and Europe (31). Adults were the included in two of the 15 SRs (25,27).

Overlap between systematic reviews

Our SR includes 21 SRs. The 21 SRs included 860 studies (Table 1), 615 of which were not duplicates. Out of the 615 studies, 477 (77.56%) were cited only once in the included SRs, and the remainder were cited in up to 10 SRs, 83 (13.50%) were cited twice, 29 (4.72%) three times, 8 (1.30%) four times, 8 (1.30%) five times, 6 (0.98%) six times, 2 (0.33%) seven times, one (0.16%) eight times and one (0.16%) ten times (Table 2, Figure 2).

For peer review only

Health Outcomes

The Supplementary File 2 reports the health outcome data extracted from the twenty-one SRs.

Clinical Outcomes Associated with ZIKV Infection During Pregnancy

The Supplemental File 2 shows that the reported outcomes associated with ZIKV infection during pregnancy ranging from adverse birth outcomes to perinatal death. The frequency of infant deaths (miscarriages, perinatal deaths, intrauterine death or stillbirth and termination of pregnancy) was reported by 6 of 21 SRs (15,18–20,30,33), ranging from 4.8% to 22%.

Congenital Zika syndrome (CZS) was reported in many different ways. Some studies reported specific outcomes related to CZS (e.g. brain abnormalities, ocular disorder or microcephaly) while others reported CZS as a nonspecific outcome. The prevalence of CZS ranged from 2% (5 cases in 250 ZIKV-infected pregnant women) (18) to 50% (58 adverse congenital outcomes out of 117 women with PCR confirmed ZIKV) (22).

Brain abnormalities were explicitly reported with data from 19 studies in which 96% (205 in 213 pregnant women) of fetuses were diagnosed after confirmation with imaging tests (15). One SR reported the prevalence of brain abnormalities (28%) including microcephaly in newborns whose mothers were infected with ZIKV in pregnancy (25) while other SR reported an observational study of 35 infants with microcephaly, 11 fetuses had intra-uterine brain injury accompanied by stunting of cerebral growth prior to birth (17). Further, five SRs classified the type of brain abnormalities or where the lesions were found (21,24,28,30,32)as intracranial calcification, reduction in the constitution of gyri of the severe cerebral cortex, abnormal hypodensity of the white matter, malformations of cortical development, subcortical-cortical junction calcifications, basal ganglia calcification, brain calcification, intraventicular synechiae and periventricular cystic, brain volume loss, ventriculomegaly / hydrocephaly and diffuse involvement of all the cerebral lobes.

Microcephaly was reported in 14 of 21 SRs. Chibueze et al. (2016) provided a trimester-specific modeling estimate risk for microcephaly. When the infection occurs in an indeterminate period of pregnancy, ZIKV associated microcephaly was described by Coelho et al. (2017). The authors performed a meta-analysis and found a prevalence of 2.3% (95% CI 1% - 5.3%) of microcephaly when considering all pregnancies (2,941 mother-infant pairs). When considering only live births (2,648 live births), the prevalence of microcephaly was 2.7% (95% CI 1.2% - 6%) (18). Nithiyanantham et al. (2019) also performed a meta-analysis of the proportion of congenital disorders in infants born to ZIKV-infected mothers, reporting a prevalence of 3.9% (95% CI 2.4% – 5.4%) (32). Pomar et al. (2019) reported the prevalence of microcephaly in CZS ranging from 33.3% to 64% (30). Four SRs reported microcephaly cases per live-birth pregnancies, ranging from 0.2% (20 cases per 10,000 live births) to 14.3% (1 case in 7 live-birth pregnancies) (15,16,18,20) and one SRs reported 10 microcephaly cases per 10,000 births (19). Microcephaly risk in infected pregnant women was reported in four SRs. The absolute risk varied between 0.95% (95% CI: 0.34 – 1.91%) during the first trimester of pregnancy to 30% (22,24–26)

For peer review only

(trimester not reported). Death caused by microcephaly was estimated in a study reported by Coelho et al. (2017), reporting a rate of 8.3% (171 deaths among 2,063 confirmed cases of microcephaly) (18). The prevalence of microcephaly in asymptomatic ZIKV infection was also reported as 0.36% (0.22% – 0.51%) (23). Another SR reported that in a series of 13 infants with congenital ZIKV infection and microcephaly, more than half of the mothers did not report any symptoms of ZIKV prior to delivery (26).

The prevalence of congenital ZIKV syndrome-related outcomes is still unknown. In this SR of SRs we found the intrauterine growth restrictions rate reported varied from 28.57% (10 cases in 35 mother-infant pairs) (15) to 31.43%, from one observational study of 35 infants with microcephaly (17). Another study reported intrauterine growth restriction in 11.9% of fetuses with or without microcephaly (5 fetuses from 42 positives for ZIKV pregnant women) (25). Pomar et al. (2019) reported the prevalence of intrauterine growth restriction in 14% of CZS cases. The prevalence of ocular disorder was reported in five SRs ranging from 0.9% % (from one study with 395 live-birth pregnancies) to 58.6% (17 ocular findings with microcephaly associated in 29 infants) (15,18,21,24,25,30,32). Abnormal amniotic fluid was described only by Krauer et al. (2017). Auditory disorder was described by Krauer et al. (2018) (prevalence of 13% - 3 cases in 24 mother-infant pairs) and Soriano-Arandes et al. (2018) (prevalence of 7% - 5 cases in 70 children with laboratory diagnosis of ZIKV infection) and Barbosa et al. (2019) (variations in the frequency of altered otoacoustic emissions testing (OAE) and automated auditory brainstem (ABR) response testing across the studies in 515 children: altered OAE varied from 0% to 75%, while altered a-ABR varied from 0% to 29.2%). The prevalence of cardiovascular damage was reported by Coelho et al. (2017) (prevalence of 1% - 3 cases in 301 live-birth pregnancies), Soriano-Arandes et al. (2018) (prevalence of 13.6% - 14 cases in103 ZIKV cases) and Minhas et al. (2017) (prevalence of 67% of heart failure in a cohort with 9 adults positive for ZIKV and no previous cardiac history).

Neurological Complications Associated with ZIKV Infection

Neurological complications were reported by 12 of 21 SRs (16,19–23,25,27,29–31,33), where GBS was the most commonly reported neurological complication.

Among adults, the proportion of neurological complications associated with ZIKV infection in Bahia (Brazil) was similar to that in French Polynesia. Among these neurological complications, GBS was diagnosed in 1 of every 1,000 reported Zika cases in Brazil and 1.3 per 1,000 in French Polynesia (16). During the French Polynesia outbreak in 2013, the incidence of GBS has been 0.24 per 1,000 ZIKV infections (20), and Simões et al. (2016) described one case report in French Polynesia in which GBS was diagnosed in a patient with ZIKV (19).

Counotte et al. (2018) reported the increased incidence of GBS incidence ratio between during and pre-ZIKV outbreak periods in seven different countries; which ranged from 2.0 (95% CI: 1.6-2.6) to 9.8 (95% CI: 7.6-12.5), while Barbi et al. (2018) conducted a meta-analysis of the prevalence of GBS in ZIKV infected cases. Their estimate for the prevalence of GBS in adults infected with ZIKV was 1.23% (CI: 95% 1.17%-1.29%). This same study was reported by Pomar et al. (2019). Krauer et al. (2017) reported

the prevalence of symptomatic ZIKV in GBS cases (74-84% symptomatic ZIKV in GBS cases). Paixão et al. (2016), Padilla et al. (2016) and Barbi et al. (2018), described the prevalence of admission to an intensive care unit (ranging from 36% to 42%, among 42 and 38 GBS cases respectively) and mechanical ventilation (21% to 29% among 42 GBS cases) in French Polynesia. The interval between ZIKV and GBS symptoms was described by Krauer et al. (2017), Paixão et al. (2016), Padilla et al. (2016) and Counotte et al. (2018). The highest interval was reported by Paixão et al. (2016), where 88% of GBS cases reported a viral syndrome up to 23 days before the onset of the neurologic syndrome. No deaths due to GBS related with ZIKV infections were reported in this SR.

Epilepsy and sleep profiles were described in two SRs. For Marques et al. (2019), the prevalence of epilepsy in congenital ZIKV infants ranged from 42% (43 in 102 children with congenital ZIKV) to 67% (95 in 141 congenital ZIKV), and 34% (30 in 88 congenital ZIKV-infected children) of the ZIKV infected children were defined as poor sleepers (21). Pomar et al. (2019) reported that 9% to 95.5% of congenital ZIKV infections were associated with epilepsy.

Idiopatic thrombocytopaenia purpura (ITP) related with ZIKV infection was reported by Counotte et al. (2018). They reported 11 cases of ITP across 18 studies; however, there is no information about the total number of ZIKV infected subjects in these studies.

Deaths Associated with ZIKV Infection

Deaths due to Zika infection are rare. According to the Brazilian Ministry of Health, between 440,000 and 1,300,000 cases of Zika occurred in Brazil in 2015 (36,37). Since the beginning of the outbreak 11 deaths among adults were confirmed in Brazil and an additional nine deaths were reported by the countries and territories in the Americas (5).

Coinfection

Coinfection was reported with dengue (16–18,25), chikungunya (16,17,25) and HIV (16,17); cytomegalovirus, toxoplasmosis, or other known teratogenic agents (16–18); hepatitis B virus (HBV), hepatitis C virus (HCV), cytomegalovirus (CMV), herpes simplex virus (HSV), Epstein-Barr virus (EBV), rubella, human T lymphotrophic virus (HTLV), parvovirus B19 and syphilis (17).

Masel et al. (2019) found no association of prior exposure to DENV and fetal loss, or clinical neurological assessment of fetus, and no statistical difference in prior DENV exposed patients with or without GBS after ZIKV infection.

Quality assessment

Of the twenty-one SRs included, there was high inter-rater reliability between the reviewers (91%). The overall quality of the SRs was critically low with all studies identified as having more than one critical weakness with or without non-critical weaknesses (Figure 3). For all studies, the majority (65%) of answers for the six critical domains of AMSTAR 2 tool (questions 2, 4, 7, 9, 12 and 14) were 'no' or 'partial yes' (53% and 12% respectively) (Figure 4 and Supplementary File 3). Main weaknesses identified

For peer review

were a deficient bibliographic search strategy and the lack of an explicit statement that SR methods were established prior to the conduct of the SR.

Discussion

Our SR of SRs identified 21 SRs that reported health outcomes associated with ZIKV infection. Microcephaly was the most commonly reported health outcome. Other outcomes reported were fetal death, neonatal death, congenital abnormalities including brain abnormalities, intrauterine growth restrictions, ocular disorders, and infant disorders including auditory disorders, cardiovascular damage, death due ZIKV infection, neurological complications, epilepsy and finally adult outcomes including GBS. The included SRs indicate that ZIKV infection is causally associated with congenital abnormalities, including microcephaly, and that ZIKV infection is a trigger of GBS, considering evidence on biological plausibility, the strength of association, and the exclusion of alternative explanations.

Overall, we found high heterogeneity among the twenty-one included SRs ranging from descriptive SRs, with few data on health outcomes associated with ZIKV infection, to more quantitative SRs, including four meta-analyses. There was some overlap (22%) of included studies across the SRs, indicating that the SRs are relatively distinct from each other and consistent with the included SRs reporting on different aspects of ZIKV infection. Given this heterogeneity it was not possible to perform a quantitative synthesis, making it difficult to compare the results or draw conclusions based on the included SRs. Further, our quality appraisal found that all SRs were of critically low quality, with only three or fewer of six critical domains of AMSTAR 2 tool met in any study.

Further research into the magnitude of effects, potential other immediate and late outcomes, and long-term sequelae is warranted to understand the full impact of ZIKV infection, particularly long-term follow up studies of infants born to ZIKV-infected mothers and infants and children infected with ZIKV early in life. In a recent study, Nielsen-Saines et al. (2019) reinforce this conclusion. They observed that the neurologic phenotype in some ZIKV-exposed children may change from abnormal to normal from birth into early childhood, and vice versa (38).

Our SR has some limitations. Since ZIKV is an emerging disease, and despite the increasing number of SRs, one limitation is the lack of SRs on ZIKV in the literature. Because the Brazilian outbreak prompted much of the recent research, 7 of 21 (33%) included SRs were conducted fairly early in the epidemic between 2016 and 2017, 43% in 2018 and 24% in 2019, which can explain the lack of information on severe outcomes related to ZIKV infection or the presence of specific outcomes, caused by the inability to observe outcomes that are only evident or possible to detect in older children. Often the reported data are unclear as to the nature of the infection, i.e. whether included subjects are suspected ZIKV cases or confirmed ZIKV cases. Further, some of the included SRs did not report denominators, making interpretation difficult.

For peer review only

The low quality of the included SRs may indicate an important publication bias related to rare (e.g., ITP) or poorly reported outcomes (e.g., sleep disorders, epilepsy and auditory disorder) as these may not be captured in the search strategy.

Our study was strengthened by using a broad search strategy, without restrictions by language or publication type, reducing selection bias. To our knowledge, this is the first SR of SRs about health outcomes associated with ZIKV infection in humans.

As SRs of SRs aim to provide a summary of evidence from other SRs, although we were not able to perform a meta-analysis, our SR synthesizes findings from SRs on health outcomes associated with ZIKV infection in humans.

The evolving nature of the literature on ZIKV-associated health outcomes together with the critically low quality of existing SRs, confirm the need for high-quality SRs to better understand the burden of ZIKV, guide patient care and inform health policy.

Conclusion

Our SR demonstrates the need for future SRs on health outcomes associated with ZIKV infection as more research is published. As the ZIKV epidemic continues to evolve and the time since the emergence of the Brazilian outbreak increases we expect more primary observational studies on associated short- and long-term health outcomes to be published and synthesized in future SRs.

For peer review only

Acknowledgements

The authors would like to acknowledge Andrea Tricco and Joanna Bielecki for their guidance on this project.

Author Contributions

Raphael Ximenes: Conceptualization of the study, performed the systematic review, critically appraising the scientific literature, analysis, drafting and revising the manuscript.

Rafael N. Miranda: Performed the systematic review, critically appraising the scientific literature, revising the manuscript.

Lauren C. Ramsay: Performed the systematic review and critically appraising the scientific literature.

Shaun K. Morris: Critical revision of the manuscript.

Kellie E. Murphy: Critical revision of the manuscript.

RADAM-LAC Research Team: Contribution to study conception and design.

Beate Sander: Conceptualization of the study, critical revision of the manuscript, supervision of the study.

Funding
This project was funded by the Canadian Institutes of Health Research - Team grant - FRN149784 and the International Development Research Centre (IDRC).

Conflicts
The authors have no conflicts of interest to declare.

Data availability

All data underlining the results are available as part of the article and no additional source data are required.

For peer review only

References

1. World Health Organization. WHO | The History of Zika Virus. Who [Internet]. 2017 [cited 2018 Dec 10]; Available from: https://www.who.int/emergencies/zika-virus/timeline/en/

2. Posen HJ, Keystone JS, Gubbay JB, Morris SK. Epidemiology of Zika virus, 1947–2007. BMJ Glob Heal [Internet]. 2016 Aug [cited 2019 Feb 9];1(2):e000087. Available from: http://www.ncbi.nlm.nih.gov/pubmed/28588942

3. Duffy MR, Chen T-H, Hancock WT, Powers AM, Kool JL, Lanciotti RS, et al. Zika Virus Outbreak on Yap Island, Federated States of Micronesia. N Engl J Med [Internet]. 2009 Jun 11 [cited 2018 Dec 10];360(24):2536–43. Available from: http://www.nejm.org/doi/abs/10.1056/NEJMoa0805715

4. Kindhauser MK, Allen T, Frank V, Santhana RS, Dye C. Zika: the origin and spread of a mosquito-borne virus. Bull World Health Organ [Internet]. 2016 Sep 1;94(9):675–686C. Available from: http://www.who.int/entity/bulletin/volumes/94/9/16-171082.pdf

5. Pan American Health Organization / World Health Organization. Zika suspected and confirmed cases reported by countries and territories in the Americas Cumulative cases, 2015-2017. Updated as of 04 January 2018 [Internet]. Pan American Health Organization. Washington, D.C.; 2017 [cited 2019 Feb 9]. Available from: https://www.paho.org/hq/index.php?option=com_docman&view=download&category_slug=cumulative-cases-pdf-8865&alias=43296-zika-cumulative-cases-4-january-2018-296&Itemid=270&lang=en

6. World Health Organization. SITUATION REPORT ZIKA VIRUS MICROCEPHALY GUILLAIN-BARRÉ SYNDROME 10 MARCH 2017 DATA AS OF 9 MARCH 2017 [Internet]. [cited 2019 Feb 9]. Available from: https://apps.who.int/iris/bitstream/handle/10665/254714/zikasitrep10Mar17-eng.pdf?sequence=1

7. CDC. Zika Travel Information | Travelers' Health | CDC [Internet]. [cited 2019 May 28]. Available from: https://wwwnc.cdc.gov/travel/page/zika-travel-information

8. Dick GW. Zika virus (II). Pathogenicity and physical properties. Trans R Soc Trop Med Hyg [Internet]. 1952 Sep 1 [cited 2019 May 28];46(5):521–34. Available from: https://academic.oup.com/trstmh/article-lookup/doi/10.1016/0035-9203(52)90043-6

9. Cao-Lormeau VM, Blake A, Mons S, Lastere S, Roche C, Vanhomwegen J, et al. Guillain-Barre Syndrome outbreak associated with Zika virus infection in French Polynesia: a case-control study. Lancet. 2016;387:1531–9.

10. de Araújo TVB, Rodrigues LC, de Alencar Ximenes RA, de Barros Miranda-Filho D, Montarroyos UR, de Melo APL, et al. Association between Zika virus infection and microcephaly in Brazil, January to May, 2016: preliminary report of a case-control study. Lancet Infect Dis [Internet]. 2016 Dec 1 [cited 2019 Jan 28];16(12):1356–63. Available from: http://www.ncbi.nlm.nih.gov/pubmed/27641777

11. Saima Nasir JA. A Bibliometric Analysis of Research on Zika Virus Indexed in Web of Science. Adv Life Sci [Internet]. 2018 [cited 2018 Dec 4];5(3):88–95. Available from: http://www.als-journal.com/532-18/

For peer review only

12. Moher D, Shamseer L, Clarke M, Ghersi D, Liberatî A, Petticrew M, et al. Preferred reporting items for systematic review and meta-analysis protocols (PRISMA-P) 2015 statement [Internet]. 2015. Available from: http://www.crd.york.ac.uk/prospero

13. AMSTAR - Assessing the Methodological Quality of Systematic Reviews [Internet]. [cited 2018 Dec 4]. Available from: https://amstar.ca/Amstar-2.php

14. Shea BJ, Reeves BC, Wells G, Thuku M, Hamel C, Moran J, et al. AMSTAR 2: a critical appraisal tool for systematic reviews that include randomised or non-randomised studies of healthcare interventions, or both. BMJ [Internet]. 2017 Sep 21 [cited 2018 Dec 4];j4008. Available from: http://www.bmj.com/lookup/doi/10.1136/bmj.j4008

15. Krauer F, Riesen M, Reveiz L, Oladapo OT, Martínez-Vega R, Porgo T V., et al. Zika Virus Infection as a Cause of Congenital Brain Abnormalities and Guillain–Barré Syndrome: Systematic Review. von Seidlein L, editor. PLOS Med [Internet]. 2017 Jan 3;14(1):e1002203. Available from: https://dx.plos.org/10.1371/journal.pmed.1002203

16. Paixão ES, Barreto F, Teixeira M da G, Costa M da CN, Rodrigues LC. History, Epidemiology, and Clinical Manifestations of Zika: A Systematic Review. Am J Public Health [Internet]. 2016 Apr 9;106(4):606–12. Available from: http://ajph.aphapublications.org/doi/10.2105/AJPH.2016.303112

17. Chibueze EC, Tirado V, Lopes K da S, Balogun OO, Takemoto Y, Swa T, et al. Zika virus infection in pregnancy: a systematic review of disease course and complications. Reprod Health [Internet]. 2017 Dec 28;14(1):28. Available from: http://reproductive-health-journal.biomedcentral.com/articles/10.1186/s12978-017-0285-6

18. Coelho A, Crovella S, Coelho AVC, Crovella S. Microcephaly Prevalence in Infants Born to Zika Virus-Infected Women: A Systematic Review and Meta-Analysis. Int J Mol Sci [Internet]. 2017 Aug 5;18(8):1714. Available from: http://www.mdpi.com/1422-0067/18/8/1714

19. Simões R, Buzzini R, Bernardo W, Cardoso F, Salomão A, Cerri G, et al. Zika virus infection and pregnancy. Rev Assoc Med Bras [Internet]. 2016 Apr;62(2):108–15. Available from: http://www.scielo.br/scielo.php?script=sci_arttext&pid=S0104-42302016000200108&lng=en&tlng=en

20. Padilla C, Pan A, Geller A, Zakowski MI. Zika virus: review and obstetric anesthetic clinical considerations. J Clin Anesth [Internet]. 2016 Dec 1;35:136–44. Available from: https://www.sciencedirect.com/science/article/pii/S0952818016304299

21. Marques V de M, Santos CS, Santiago IG, Marques SM, Nunes Brasil M das G, Lima TT, et al. Neurological Complications of Congenital Zika Virus Infection. Pediatr Neurol [Internet]. 2019 Feb;91:3–10. Available from: http://www.ncbi.nlm.nih.gov/pubmed/30591235

22. Counotte MJ, Egli-Gany D, Riesen M, Abraha M, Porgo TV, Wang J, et al. Zika virus infection as a cause of congenital brain abnormalities and Guillain-Barré syndrome: From systematic review to living systematic review. PLoS Med [Internet]. 2018 Feb 15;7:196. Available from: http://www.ncbi.nlm.nih.gov/pubmed/30631437

23. Haby MM, Pinart M, Elias V, Reveiz L. Prevalence of asymptomatic Zika virus infection: a systematic review. Bull World Health Organ [Internet]. 2018 Jun 1;96(6):402–413D. Available

For peer review only

from: http://www.ncbi.nlm.nih.gov/pubmed/29904223

24. Rehan Sarwar M, Saqib A, Iftikhar S. Zika Virus Infection during Pregnancy; Maternofetal Risk Assessment, Transmission, Complications, and Management: A Review of the Literature. Arch Clin Infect Dis [Internet]. 2018 Jun 24;13(3). Available from: http://archcid.com/en/articles/12848.html

25. Wahid B, Ali A, Waqar M, Idrees M. An updated systematic review of Zika virus-linked complications. Asian Pac J Trop Med [Internet]. 2018;11(1):1. Available from: http://www.apjtm.org/text.asp?2018/11/1/1/223527

26. Soriano-Arandes A, Rivero-Calle I, Nastouli E, Espiau M, Frick M, Alarcon A, et al. What we know and what we don't know about perinatal Zika virus infection: a systematic review. Expert Rev Anti Infect Ther [Internet]. 2018 Mar 4;16(3):243–54. Available from: http://www.ncbi.nlm.nih.gov/pubmed/29415586

27. Barbi L, Coelho AVC, Alencar LCA de, Crovella S. Prevalence of Guillain-Barré syndrome among Zika virus infected cases: a systematic review and meta-analysis. Brazilian J Infect Dis [Internet]. 2018 Mar;22(2):137–41. Available from: http://www.ncbi.nlm.nih.gov/pubmed/29545017

28. Santos GRB dos, Aragão FBA, Lobão WJ de M, Lima FR, Andrade LMRL de, Furtado QR, et al. Relationship between microcephaly and Zika virus during pregnancy: a review. Rev Assoc Med Bras [Internet]. 2018 Jul;64(7):635–42. Available from: http://www.ncbi.nlm.nih.gov/pubmed/30365666

29. Wachira VK, Peixoto HM, Fernandes De Oliveira MR. Systematic review of factors associated with the development of Guillain-Barr e syndrome 2007-2017: what has changed? 2018; Available from: https://v2dis-prod.evidencepartners.com/Generic/getAttachment2.php?id=44

30. Pomar L, Musso D, Malinger G, Vouga M, Panchaud A, Baud D. Zika virus during pregnancy: From maternal exposure to congenital Zika virus syndrome. Prenat Diagn [Internet]. 2019 May [cited 2019 Aug 1];39(6):420–30. Available from: http://www.ncbi.nlm.nih.gov/pubmed/30866073

31. Wilder-Smith A, Chang CR, Leong WY. Zika in travellers 1947-2017: a systematic review. J Travel Med [Internet]. 2018 [cited 2019 Aug 1];25(1). Available from: http://www.ncbi.nlm.nih.gov/pubmed/30016469

32. Nithiyanantham SF, Badawi A. Maternal infection with Zika virus and prevalence of congenital disorders in infants: systematic review and meta-analysis. Can J Public Heal [Internet]. 2019 May 10 [cited 2019 Aug 1]; Available from: http://www.ncbi.nlm.nih.gov/pubmed/31077071

33. Masel J, McCracken MK, Gleeson T, Morrison B, Rutherford G, Imrie A, et al. Does prior dengue virus exposure worsen clinical outcomes of Zika virus infection? A systematic review, pooled analysis and lessons learned. Diemert DJ, editor. PLoS Negl Trop Dis [Internet]. 2019 Jan 25 [cited 2019 Aug 1];13(1):e0007060. Available from: http://www.ncbi.nlm.nih.gov/pubmed/30682026

34. Barbosa MH de M, Magalhães-Barbosa MC de, Robaina JR, Prata-Barbosa A, Lima MA de MT de, Cunha AJLA da. Auditory findings associated with Zika virus infection: an integrative review. Braz J Otorhinolaryngol [Internet]. 2019 Jun 18 [cited 2019 Aug 1]; Available from: http://www.ncbi.nlm.nih.gov/pubmed/31296482

For peer review only

35. Minhas AM, Nayab A, Iyer S, Narmeen M, Fatima K, Khan MS, et al. Association of Zika Virus with Myocarditis, Heart Failure, and Arrhythmias: A Literature Review. Cureus [Internet]. 2017 Jun 27 [cited 2019 Aug 1];9(6):e1399. Available from: http://www.ncbi.nlm.nih.gov/pubmed/28856072

36. Anthony Boadle LP. Exclusive: Brazil says Zika virus outbreak worse than believed | Reuters [Internet]. Reuters. 2016 [cited 2018 Dec 4]. Available from: https://www.reuters.com/article/us-health-zika-brazil-exclusive-idUSKCN0VA331

37. World Health Organization. ZIKA SITUATION REPORT - ZIKA AND POTENTIAL COMPLICATIONS 12 FEBRUARY 2016 [Internet]. 2016 [cited 2018 Dec 4]. Available from: https://www.who.int/emergencies/zika-virus/situation-report/who-zika-situation-report-12-02-2016.pdf

38. Nielsen-Saines K, Brasil P, Kerin T, Vasconcelos Z, Gabaglia CR, Damasceno L, et al. Delayed childhood neurodevelopment and neurosensory alterations in the second year of life in a prospective cohort of ZIKV-exposed children. Nat Med [Internet]. 2019 Aug 8 [cited 2019 Aug 14];25(8):1213–7. Available from: http://www.nature.com/articles/s41591-019-0496-1

39. Brasil P, Pereira JP, Moreira ME, Ribeiro Nogueira RM, Damasceno L, Wakimoto M, et al. Zika Virus Infection in Pregnant Women in Rio de Janeiro. N Engl J Med [Internet]. 2016 Dec 15 [cited 2019 Aug 6];375(24):2321–34. Available from: http://www.ncbi.nlm.nih.gov/pubmed/26943629

40. Mlakar J, Korva M, Tul N, Popović M, Poljšak-Prijatelj M, Mraz J, et al. Zika Virus Associated with Microcephaly. N Engl J Med [Internet]. 2016 Mar 10 [cited 2019 Aug 6];374(10):951–8. Available from: http://www.ncbi.nlm.nih.gov/pubmed/26862926

41. de Paula Freitas B, de Oliveira Dias JR, Prazeres J, Sacramento GA, Ko AI, Maia M, et al. Ocular Findings in Infants With Microcephaly Associated With Presumed Zika Virus Congenital Infection in Salvador, Brazil. JAMA Ophthalmol [Internet]. 2016 May 1 [cited 2019 Aug 6];134(5):529. Available from: http://www.ncbi.nlm.nih.gov/pubmed/26865554

42. Schuler-Faccini L, Ribeiro EM, Feitosa IML, Horovitz DDG, Cavalcanti DP, Pessoa A, et al. Possible Association Between Zika Virus Infection and Microcephaly — Brazil, 2015. MMWR Morb Mortal Wkly Rep [Internet]. 2016 Jan 29 [cited 2019 Aug 6];65(3):59–62. Available from: http://www.ncbi.nlm.nih.gov/pubmed/26820244

43. Cao-Lormeau V-M, Blake A, Mons S, Lastère S, Roche C, Vanhomwegen J, et al. Guillain-Barré Syndrome outbreak associated with Zika virus infection in French Polynesia: a case-control study. Lancet [Internet]. 2016 Apr 9 [cited 2018 Dec 4];387(10027):1531–9. Available from: https://www.sciencedirect.com/science/article/pii/S0140673616005626

44. Honein MA, Dawson AL, Petersen EE, Jones AM, Lee EH, Yazdy MM, et al. Birth Defects Among Fetuses and Infants of US Women With Evidence of Possible Zika Virus Infection During Pregnancy. JAMA [Internet]. 2017 Jan 3 [cited 2019 Aug 6];317(1):59. Available from: http://jama.jamanetwork.com/article.aspx?doi=10.1001/jama.2016.19006

45. Meaney-Delman D, Hills SL, Williams C, Galang RR, Iyengar P, Hennenfent AK, et al. Zika Virus Infection Among U.S. Pregnant Travelers — August 2015–February 2016. MMWR Morb Mortal Wkly Rep [Internet]. 2016 Mar 4 [cited 2019 Aug 6];65(8):211–4. Available from: http://www.ncbi.nlm.nih.gov/pubmed/26938703

For peer review only

46. Cauchemez S, Besnard M, Bompard P, Dub T, Guillemette-Artur P, Eyrolle-Guignot D, et al. Association between Zika virus and microcephaly in French Polynesia, 2013–15: a retrospective study. Lancet [Internet]. 2016 May 21 [cited 2019 Aug 6];387(10033):2125–32. Available from: http://www.ncbi.nlm.nih.gov/pubmed/26993883

47. Oliveira Melo AS, Malinger G, Ximenes R, Szejnfeld PO, Alves Sampaio S, Bispo de Filippis AM. Zika virus intrauterine infection causes fetal brain abnormality and microcephaly: tip of the iceberg? Ultrasound Obstet Gynecol [Internet]. 2016 Jan 1 [cited 2019 Aug 6];47(1):6–7. Available from: http://doi.wiley.com/10.1002/uog.15831

48. Besnard M, Eyrolle-Guignot D, Guillemette-Artur P, Lastère S, Bost-Bezeaud F, Marcelis L, et al. Congenital cerebral malformations and dysfunction in fetuses and newborns following the 2013 to 2014 Zika virus epidemic in French Polynesia. Eurosurveillance [Internet]. 2016 Mar 31 [cited 2019 Aug 6];21(13):30181. Available from: http://www.ncbi.nlm.nih.gov/pubmed/27063794

49. van der Linden V, Pessoa A, Dobyns W, Barkovich AJ, Júnior H van der L, Filho ELR, et al. Description of 13 Infants Born During October 2015–January 2016 With Congenital Zika Virus Infection Without Microcephaly at Birth — Brazil. MMWR Morb Mortal Wkly Rep [Internet]. 2016 Dec 2 [cited 2019 Aug 6];65(47):1343–8. Available from: http://www.cdc.gov/mmwr/volumes/65/wr/mm6547e2.htm

50. Soares de Oliveira-Szejnfeld P, Levine D, Melo AS de O, Amorim MMR, Batista AGM, Chimelli L, et al. Congenital Brain Abnormalities and Zika Virus: What the Radiologist Can Expect to See Prenatally and Postnatally. Radiology [Internet]. 2016 Oct [cited 2019 Aug 6];281(1):203–18. Available from: http://www.ncbi.nlm.nih.gov/pubmed/27552432

51. Calvet G, Aguiar RS, Melo ASO, Sampaio SA, de Filippis I, Fabri A, et al. Detection and sequencing of Zika virus from amniotic fluid of fetuses with microcephaly in Brazil: a case study. Lancet Infect Dis [Internet]. 2016 Jun [cited 2019 Aug 6];16(6):653–60. Available from: http://www.ncbi.nlm.nih.gov/pubmed/26897108

52. França GVA, Schuler-Faccini L, Oliveira WK, Henriques CMP, Carmo EH, Pedi VD, et al. Congenital Zika virus syndrome in Brazil: a case series of the first 1501 livebirths with complete investigation. Lancet [Internet]. 2016 Aug 27 [cited 2019 Aug 6];388(10047):891–7. Available from: http://www.ncbi.nlm.nih.gov/pubmed/27372398

53. Besnard M, Lastère S, Teissier A, Cao-Lormeau V, Musso D. Evidence of perinatal transmission of Zika virus, French Polynesia, December 2013 and February 2014. Eurosurveillance [Internet]. 2014 Apr 3 [cited 2019 Aug 6];19(13):20751. Available from: http://www.eurosurveillance.org/ViewArticle.aspx?ArticleId=20751

54. Kleber de Oliveira W, Cortez-Escalante J, De Oliveira WTGH, do Carmo GMI, Henriques CMP, Coelho GE, et al. Increase in Reported Prevalence of Microcephaly in Infants Born to Women Living in Areas with Confirmed Zika Virus Transmission During the First Trimester of Pregnancy — Brazil, 2015. MMWR Morb Mortal Wkly Rep [Internet]. 2016 Mar 11 [cited 2019 Aug 6];65(9):242–7. Available from: http://www.ncbi.nlm.nih.gov/pubmed/26963593

55. Oehler E, Watrin L, Larre P, Leparc-Goffart I, Lastere S, Valour F, et al. Zika virus infection complicated by Guillain-Barre syndrome--case report, French Polynesia, December 2013. Euro Surveill [Internet]. 2014 Mar 6 [cited 2019 Aug 16];19(9). Available from:

For peer review only

http://www.ncbi.nlm.nih.gov/pubmed/24626205

For peer review only

Table 1. Summary of included systematic reviews

Author, Year	Aim	Search period	Number of studies included	Types of studies included in review	Jurisdictions of included studies (n studies)
Krauer et al. (2017) (15)	To assess the relationship between ZIKV infection and congenital brain abnormalities and Guillain-Barré syndrome	From inception until May 30, 2016	106	Case reports, case series, case-control studies, cohort studies, cross-sectional studies, ecological study/outbreak reports, modelling studies, animal experiments, in vitro experiments, sequence analysis and phylogenetics	Brazil (6), Cabo Verde (2), Colombia (1), French Polynesia (2), Martinique (2), Panama (5), El Salvador (1), Haiti (119), Puerto Rico (1), Venezuela (1), Slovenia*, Netherlands*, Dominican Republic*, French Guiana*, Honduras*, Paraguay*, Suriname*, Micronesia*, Pacific Islands*<br>* Not possible to know number of studies from these countries
Paixão et al. (2016) (16)	To summarize current knowledge on ZIKV including epidemiology, clinical presentation, and complications	1954 to Jan 2016	41	Case reports, case series, surveillance reports, cross-sectional studies, epidemiological bulletins and alerts	Not clearly reported.<br>Most data are from Brazil and French Polynesia.
Chibueze et al. (2017) (17)	To summarize guidance on pregnancy care in the context of ZIKV infection	From inception until March 3, 2016	18	Case reports, case series, observational studies	Brazil (11)<br>Colombia (1)<br>France (1)<br>Puerto Rico (1)<br>Slovenia (1)<br>USA (2)<br>Venezuela (1)
Coelho et al. (2017) (18)	To summarize evidence and meta-analyze data to estimate prevalence	Not reported	8	Cohort studies	Brazil (1)<br>Colombia (1)<br>French Guiana (1)<br>Puerto Rico (1)

For peer review only

Author, Year	Aim	Search period	Number of studies included	Types of studies included in review	Jurisdictions of included studies (n studies)
	of microcephaly in babies born to ZIKV infected pregnant women				USA (4)
Simões et al. (2016) (19)	To assess the effects of Zika virus infection on during pregnancy and postpartum periods	From inception until Feb 23, 2016	30	Case reports, case series, guidelines	Not clearly reported; most data are from Brazil.
Padilla et al. (2016) (20)	To review clinical and basic science literature about ZIKV infection relevant for obstetric anesthesiologists	From inception until Apr 15, 2016	30	Systematic reviews, narrative reviews, case reports, epidemiologic studies, government reports, and news articles	Not clearly reported.
Marques et al. (2019) (21)	To map the neurological damage and outcomes related to congenital ZIKV infection	Jan 1966 to Aug 2018	28	Not informed	Brazil (16)<br>USA (3)<br>Colombia (1)
Counotte et al. (2018) (22)	To summarize the evidence of the casual associations between ZIKV and CZS and GBS	May 30, 2016 to Jan 18, 2017	101	Case report, case series, case-control study, cohort study, cross-sectional study, controlled trials, ecological study/outbreak report, modelling study, animal experiment, in vitro experiment, sequencing and phylogenetics, biochemical/protein structure studies	USA, Martinique, Brazil, Suriname, Colombia, French Guiana, Slovenia, Spain, Uganda, Nicaragua, Barbados, Belize, Dominican Republic, El Salvador, Guatemala, Haiti, Honduras; Mexico, Republic of Marshall Islands, Venezuela, French Polynesia, Ecuador, France, Puerto Rico, Guadeloupe,

For peer review only

Author, Year	Aim	Search period	Number of studies included	Types of studies included in review	Jurisdictions of included studies (n studies)
					Guyana, New Zealand, French Southern Territories
Haby et al., (2018) (23)	To estimate and meta-analyze the prevalence of asymptomatic Zika virus infection in the general population and in specific population groups from observational epidemiological studies	From inception until Jan 26, 2018	23	Cross-sectional seroprevalence studies, case series, case-control, cohort	USA (6), Brazil (3), French Polynesia (3), French Guiana (3), Puerto Rico (2), Colombia (2), Spain (2), Micronesia (1), Martinique (1)
Sarwar et al. (2018) (24)	To report on the current literature regarding ZIKV and its hazardous effects on maternofetal health with a special emphasis on risk assessment, virus transmission, associated complications, and possible management	2007 to May 2017	69	Not informed	Argentina, Bolivia, Brazil, Colombia, French Guiana, Suriname, Paraguay, Trinidad and Tobago, Canada, Dominican Republic, Grenada, Guadeloupe, Guatemala, Haiti, Martinique, Puerto Rico, USA, Costa Rica, El Salvador, Honduras, Nicaragua, Panama, Europe, Slovenia, Spain, Thailand, Vietnam, French Polynesia, Marshall Islands, Cape Verde
Wahid et al. (2018) (25)	To summarize the evidence of neurological complications in ZIKV-infected people	2015 to March 2017	35	Case-studies, case-cohort studies, cross-sectional studies, organizational survey reports and case-control studies	Brazil (15)<br>French Polynesia (4)<br>Colombia (3)

For peer review only

Author, Year	Aim	Search period	Number of studies included	Types of studies included in review	Jurisdictions of included studies (n studies)
					USA, Slovenia, Suriname, Spain, Haiti, Martinique, Netherlands, Ecuador, Guyana (1)
Soriano-Arandes et al. (2018) (26)	To summarize the new evidence in aspects of epidemiology, virology, pathogenesis, associated risk factors during pregnancy, newborn phenotypic signs, neuroimaging, laboratory diagnosis, treatment and vaccines	From inception until Nov 30, 2017	106	Case series, cohort (prospective/retrospective), cross-sectional or case-control studies	Brazil, French Polynesia, USA, Martinique, Colombia
Barbi et al. (2018) (27)	To systematically review the literature and perform a meta-analysis to estimate the prevalence of GBS among ZIKV-infected individuals	From inception until Nov 2017	3	Case series, epidemiological surveys, cross-sectional or cohort studies	French Polynesia (1), Suriname and Dominican Republic (1), South American, Central American and Caribbean countries (1)
Santos et al. (2018) (28)	To analyze the association between Zika-virus and microcephaly during the gestational period	From inception until Dec 2016	35	Not informed	Brazil
Wachira et al. (2018) (29)	To describe the factors associated with development of GBS, both infectious and	Jan 1, 2007 to Jun 30, 2017	34	The most common were case control, cohort, self-controlled case series	French Polynesia

For peer review only

Author, Year	Aim	Search period	Number of studies included	Types of studies included in review	Jurisdictions of included studies (n studies)
	non-infectious, through a SR.				
Pomar et al. (2019) (30)	Present a review to describe the risks and complications of maternal and subsequent fetal infection by ZIKV.	Jun 2009 to Nov 2018	68	Not informed	Colombia (3), Puerto Rico (1), French Guiana (3), Brazil (1), Yap Island (1), USA (2)
Wilder-Smith et al. (2018) (31)	Describe the burden of ZIKV infection in international travelers over time; estimate the proportion of birth defects as a result of maternal ZIKV infection in travelers; track the extent of sexual transmission; summarize data on ZIKV cases in travelers identifying counties with reports on local transmission	1947 to Apr 2017	65	Surveillance reports, case reports, retrospective (multi-centre study), descriptive retrospective analysis and prospective cohort study	USA (9), Canada (2), Germany (3), Norway (1), France (5), Italy (7), Japan (2), Australia (4), New Caledonia (1), Finland (1), Mexico (1), Slovenia (1), Netherlands (4), Belgium (1), Portugal (1), Switzerland (3), Israel (1), Taiwan (2), Spain (1), China (7), South Korea (2), UK (2), Singapore (1), Malaysia (1)
Nithiyanantham et al. (2019) (32)	To conduct a systematic review and meta-analysis on the prevalence of congenital Zika-related disorders in infants of mothers	From inception until Oct 31, 2017	25	Case series, epidemiological reports, prospective and retrospective studies, cohort studies and cross-sectional studies	USA (8), Brazil (6), Colombia (2), Puerto Rico (1), French Polynesia (1), Martinique (1), Trinidad and Tobago (1), French Guiana (1), Ecuador (1), Spain (1)

For peer review only

Author, Year	Aim	Search period	Number of studies included	Types of studies included in review	Jurisdictions of included studies (n studies)
	infected with ZIKV during pregnancy.				
Masel et al. (2019) (33)	To determine if prior infection with DENV, as compared with those with no prior DENV infection, is associated with a greater risk of ZIKV complications (including neurological and teratogenic outcomes), greater ZIKV peak viremia, greater area-under-the-curve of viremia or other putative laboratory proxies of ZIKV severity.	From inception until Mar 25, 2018	5	Case control study	Brazil (2), French Polynesia (5)
Barbosa et al. (2019) (34)	To describe the auditory alterations, pathogenesis and recommendations for follow-up in individuals with prenatal or acquired ZIKV infection.	From inception until Apr 2019	27	Case report and case series	Brazil (14), Colombia (3), USA (2), French Polynesia (1), Puerto Rico (1)
Minhas et al. (2017) (35)	Focuses on the potential threat that ZIKV may pose to the heart like that of	From inception until March 2017	3	Case report and prospective observational multicenter study	France (1), Venezuela (1), China (1)

For peer review only

Author, Year	Aim	Search period	Number of studies included	Types of studies included in review	Jurisdictions of included studies (n studies)
	similar arboviral diseases.				

For peer review only

Table 2. Overlap between systematic reviews

Number of citations	Title	Author	Cited by
10	Zika virus infection in pregnant women in Rio de Janeiro	Brasil et al. (2016) (39)	(15,17,18,20–22,25,26,30,32)
8	Zika virus associated with microcephaly	Mlakar et al. (2016) (40)	(15,17,19,24–26,28,31)
7	Ocular findings in infants with microcephaly associated with presumed Zika virus congenital infection in Salvador, Brazil	de Paula Freitas et al. (2016) (41)	(15,17,19–21,25,30)
7	Possible association between Zika virus infection and microcephaly - Brazil, 2015	Schuler-Faccini et al. (2016) (42)	(15,17,21,25,26,28,30)
6	Guillain-Barre Syndrome outbreak associated with Zika virus infection in French Polynesia: a case-control study	Cao-Lormeau et al. (2016) (43)	(15,20,23–25,33)
6	Birth defects among fetuses and infants of US women with evidence of possible Zika virus infection during pregnancy	Honein et al. (2017) (44)	(18,21,22,24,26,32)
6	Zika virus infection among U.S. pregnant travelers - August 2015 - February 2016	Meaney-Delman et al. (2016) (45)	(15,17,18,20,31,32)
6	Zika virus outbreak on Yap Island, Federated States of Micronesia	Duffy et al. (2009) (3)	(15,16,19,23,24,30)
6	Association between Zika virus and microcephaly in French Polynesia, 2013 - 15: a retrospective study	Cauchemez et al. (2016) (46)	(15,17,24–26,30)
6	Zika virus intrauterine infection causes fetal brain abnormality and microcephaly: tip of the iceberg?	Oliveira et al. (2016) (47)	(15,17,20,21,26,28)
5	Congenital cerebral malformations and dysfunction in fetuses and newborns following the 2013 to 2014 Zika virus epidemic in French Polynesia	Besnard et al. (2016) (48)	(15,25,30,32,34)
5	Description of 13 infants born during October 2015 - January 2016 with congenital Zika virus infection without microcephaly at birth - Brazil	van der Linden et al. (2016) (49)	(21,22,26,30,34)
5	Congenital brain abnormalities and Zika virus: what the radiologist can expect to see prenatally and postnatally	Oliveira-Szejnfeld et al. (2016) (50)	(21,22,26,30,32)
5	Detection and sequencing of Zika virus from amniotic fluid of fetuses with microcephaly in Brazil: a case study	Calvet et al. (2016) (51)	(15,17,19,28,30)

For peer review only

5	Congenital Zika virus syndrome in Brazil: a case series of the first 1501 livebirths with complete investigation	França et al. (2016) (52)	(21,22,25,26,30)
5	Evidence of perinatal transmission of Zika virus, French Polynesia, December 2013 and February 2014	Besnard et al. (2014) (53)	(16,17,24,26,28)
5	Increase in reported prevalence of microcephaly in infants born to women living in areas with confirmed Zika virus transmission during the first trimester of pregnancy - Brazil, 2015	Oliveira et al. (2016) (54)	(15,17,20,24,25)
5	Zika virus infection complicated by Guillain-Barre syndrome - case report, French Polynesia, December 2013	Oehler et al. (2014) (55)	(15,16,19,20,25)

Figure legends

Figure 1: PRISMA flow diagram of search results and study selection.

Figure 2: Overlap between studies cited in at least 5 systematic reviews.

Figure 3: Individual study results of quality assessment using AMSTAR 2 - Result for all questions of AMSTAR 2 tool.

Figure 4: Individual study results of quality assessment using AMSTAR 2 - Critical domains of AMSTAR 2 tool.

For peer review only

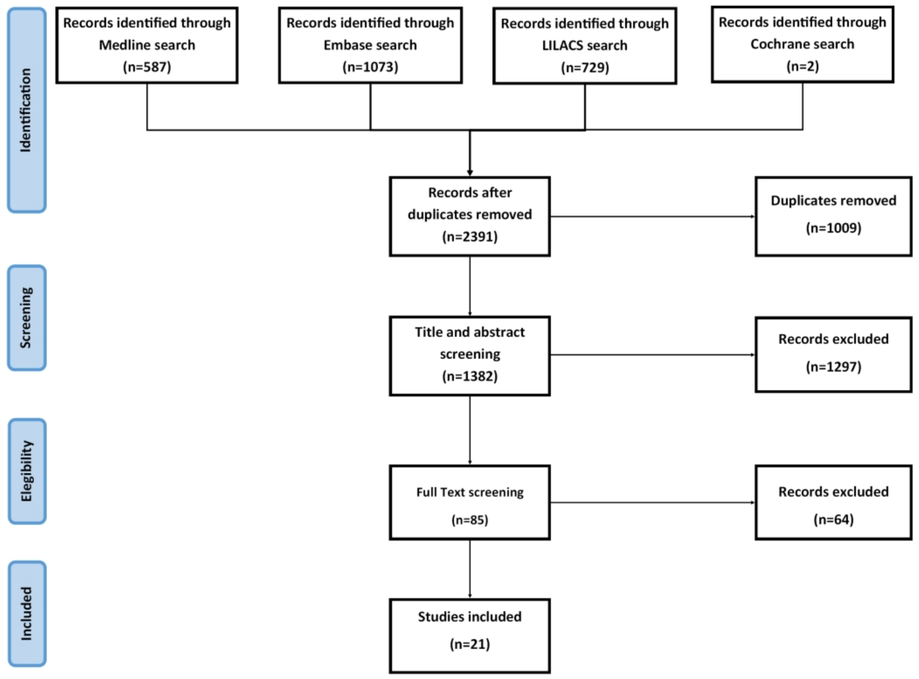

PRISMA flow diagram of search results and study selection.

279x215mm (300 x 300 DPI)

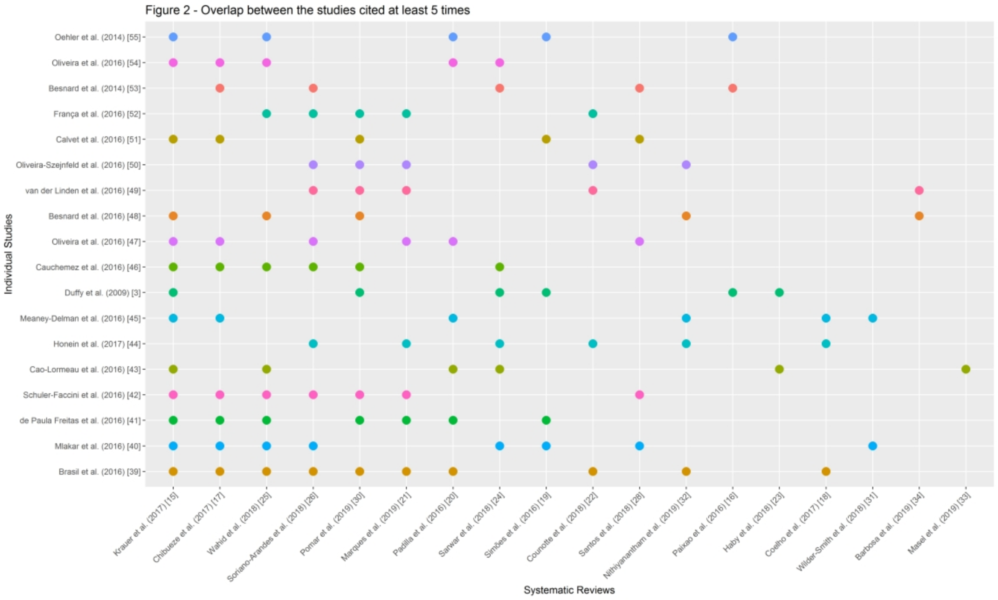

Overlap between studies cited in at least 5 systematic reviews.

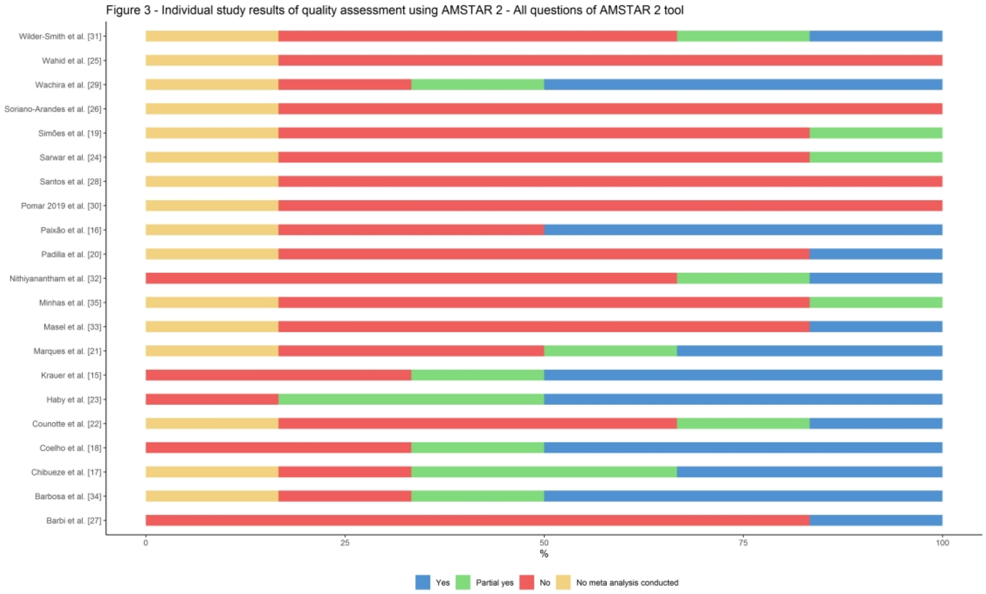

Individual study results of quality assessment using AMSTAR 2 - Result for all questions of AMSTAR 2 tool.

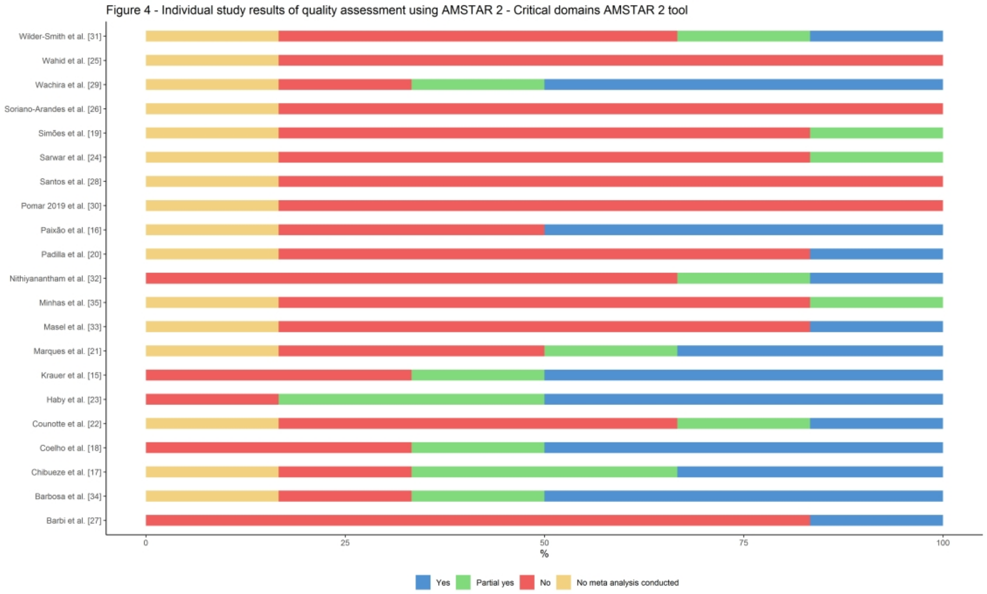

Individual study results of quality assessment using AMSTAR 2 - Critical domains of AMSTAR 2 tool.

Search Strategy

Database: Embase Classic+Embase <1947 to 2018 February 27>

Search Strategy:

1 zika fever/ or zika virus/ or zika virus vaccine/ or zika.mp. (5013)

2 "systematic review"/ or "review"/ (2367967)

3 1 and 2 (569)

Database: Ovid MEDLINE(R) <1946 to February Week 3 2018>

Search Strategy:

1 exp ZIKA VIRUS INFECTION/ or exp ZIKA VIRUS/ or zika.mp. (2287)

2 "review"/ (2215441)

3 1 and 2 (326)

Database: Cochrane

Search Strategy:

1 ZIKA and review (2)

Update – 22/07/2019

Database: LILACS

Search Strategy:

(tw:((tw:(ZIKA VIRUS INFECTION)) OR (tw:(ZIKA VIRUS)) OR (tw:(zika.mp)))) AND (tw:(systematic review)) (729)

Database(s): Ovid MEDLINE(R) 1946 to July Week 2 2019

Search Strategy:

1 exp ZIKA VIRUS INFECTION/ or exp ZIKA VIRUS/ or zika.mp. (4560)

2 "review"/ (2360456)

3 1 and 2 (722)

4 limit 3 to yr="2018-Current" (261)

Database(s): Embase Classic+Embase <1947 to 2019 July 19>

Search Strategy:

1 zika fever/ or zika virus/ or zika virus vaccine/ or zika.mp. (8593)

2 "systematic review"/ or "review"/ (2553024)

3 1 and 2 (1056)

4 limit 3 to yr="2018-Current" (504)

For peer review only

Database: Cochrane

Search Strategy:

1 ZIKA and review (0)

For peer review only

Supplementary file 2 - Table 1. Health outcomes - Congenital Zika syndrome

Authors	Malformations / Congenital abnormalities	Brain abnormalities	Microcephaly	Ocular disorders	Auditory disorder - Rate	Cardiovascular damage
Krauer et al. (2017) [15]		Prevalence: 96% of cases	91% of cases; Prevalence ratio over states with no reported cases of microcephaly= 4.67	Prevalence: 42% (49 cases in 116 mother-infant pairs)	13% (3 cases in 24 mother-infant pairs)	
Paixao et al. (2016) [16]		In 2015, the prevalence of microcephaly in Brazil was 20 cases per 10,000 live births; Zika infection during 9 pregnancies confirmed by CDC resulted in the birth of a neonate with microcephaly.	Rate per 100,000 live births: 121.7 (0.12%) in 2015 in Brazil; Death due microcephaly: 1.3% in suspected microcephaly cases			
Chibueze et al. (2017) [17]		In one observational study of 35 infants with microcephaly, 11 fetuses had intra-uterine brain injury accompanied by stunting of cerebral growth prior to birth.	One observational study provided a trimester-specific modelling estimate risk for microcephaly per 10,000 ZIKV infected pregnant women per trimester of pregnancy: 1st 95 (34 - 191), 2nd 84 (12 - 196), 3rd trimester: 0 - (0 - 251)			
Coelho et al. (2017) [18]	Other organs damage: French Guiana 2% in 250 live births or mother-infant pair. USA: 7%. Not clear if the denominator is the number of live births or mother-infant pair (301 or 498 respectively)		0.3% in live-birth pregnancies; 14.3% - in live-birth pregnancies; Prevalence (cases/all pregnancies): 2.3%. Prevalence (cases/live births): 2.7%. Death due to microcephaly: 8.3%, would be 5.7% in case of new confirmed cases are included.	Two studies reported a prevalence of ocular damage (0.9% and 1%). It is not clear if the denominator is the number of live births (395 and 301, respectively) or the number mother-infant pair (442 and 498, respectively)		French Guiana: Cardiovascular damage equal to 1%. The denominator is unclear if is the number of live births or mother-infant pair (301 and 498 respectively)
Simoes et al. (2016) [19]	Prevalence of CZS: 10 to 20 cases in 100,000 live births; 8.87% of cases with confirmed changes in CNS		The Ministry of Health in Brazil reported an increase in the number of cases of microcephaly close to 20 times that previously reported (aproximately 0.5 cases for each 10,000 live births) which means 10 microcephaly cases per 10,000 births.			
Padilla et al. (2016) [20]	In 72 women with Zika-positive serology during pregnancy in Brazil, 29% had abnormalities detected on fetal ultrasound. Central nervous system abnormalities were noted after Zika infections as late as 27 weeks' gestation, and placental insufficiency was noted with even later gestational ages.		In 2015, the prevalence of microcephaly in Brazil was 20 cases per 10,000 live births; Zika infection during 9 pregnancies confirmed by CDC resulted in the birth of a neonate with microcephaly.			
Marques et al. (2019) [21]		% of neurological malformations: Subcortical-cortical junction calcifications: 92.9%, Basal ganglia calcifications: 57.1%, Periventricular calcifications: 29.5%. Ventriculomegaly/hydrocephaly: 63.1%. Cerebellar abnormalities: 46.2%, 82% (14 of 17 patients). Corpus callosum abnormalities: 47.9%	39.7% in cases of congenital Zika infection. Almost 100% when the infection occurred during the first trimester and decreased when the infection occurred in the second or third trimester	Prevalence: 44.3% in congenital ZIKV infection, 20% in patients with microcephaly, 33% in patients with ventriculomegaly, and 43% in patients with calcification. Bilateral findings: 76.8% of infants with ocular lesions. In eyes of infants with ocular lesions and congenital ZIKV infection: Macular lesions in 50%, Optical nerve abnormalities: 27.78%, Chorioretinal artrophy/scaring: 10.65%, Focal pigment mottling of retina: 6.94%, Microphtalmia: 3.70%, Glaucoma: 2.31%, Cataract: 2.31%, Iris coloboma: 2.31%, Subluxation: 1.39%		
Counotte et al. (2018) [22]	Prevalence of adverse congenital outcomes: 8.97-49.57% in ZIKV positive women. Birth defects: 5.9% in pregnant asymptomatic women and 5.98% in symptomatic pregnant women		RR between ZIKV exposed and unexposed: 4.4-6.6. OR between women with confirmed ZIKV and without evidence of ZIKV infection: 11.0-55.5			
Haby et al. (2018) [23]			Prevalence of asymptomatic ZIKV infection in mothers who gave birth to babies with microcephaly: 0.36			

For peer review only - http://bmjopen.bmj.com/site/about/guidelines.xhtml

Authors	Malformations / Congenital abnormalities	Brain abnormalities	Microcephaly	Ocular disorders	Auditory disorder - Rate	Cardiovascular damage
Sarwar et al. (2018) [24]		Prevalence in dead neonates of ZIKV infected mothers: Pachygyria: 14.28%, Arthrogryposis: 14.28%. Morphologic microcephalus changes: 14.28%. Ventriculomegaly / hydrocephaly: 100%. Cerebellar abnormalities: 28.57%	Risk of 1% when infection occurred in the first trimester of pregnancy	In ZIKV infected infants: Retinal impairment: 28%, Impaired optic nerve: 17%, Optic nerve hypoplasia: 8%		
Wahid et al. (2018) [25]	Fetal abnormalities 28.57% in infected pregnant women. Ventricular calcifications or other central nervous system abnormal amniotic fluid volume or cerebral or umbilical artery flow: 16.67%. (CNS) lesions: 16.67%. 80 of the 185 infants, ZIKV-linked microcephaly: 10 (the value of the denominator is not clear) neonates, 5 of 80 or 185 birth defects such as hydranencephaly, holoprosencephaly, clubfeet, and craniosynostosis, 3 of 80 or 185: cataracts, holoprosencephaly, and ventral pons hypoplasia	Prevalence: 28% (including microcephaly) in newborns of mothers infected with ZIKV	Risk of microcephaly: 0-30%. Relative Risk 100–1,000 (assuming 10% exposure) or 20–200 (assuming 50% exposure) compared to background risk of microcephaly. Prevalence: 50.47% among definite or probable ZIKV cases. Higher risk of microcephaly in pregnant women infected during first trimester. Estimated risk of microcephaly: 0.95% in women infected in the first trimester	In infants with microcephaly: ophthalmoscopic alterations in 50% (not clear if ZIKV-related infection) . Ocular findings 34.5-58.62% of ZIKV linked microcephalic infants		
Soriano-Arandes et al. (2018) [26]	Birth defects: 6% in asymptomatic and symptomatic pregnant women. From 1 study: Fetal adverse outcomes in women infected with ZIKV: 55% in the first term of pregnancy, 29% in the third trimester. In infants with CZS: Dimples: 30.1%, Distal hand/finger contractures: 20.5%, feet malposition: 15.7%, generalized arthrogryposis: 9.6%, birth defects in women with recent ZIKV infection: 6%	Prevalence: Microcephaly in 86.7% and craniofacial disproportion in 95.8% of infants with probable CZS	In infected women in the first trimester: Risk of 0.95% in a population with an estimated rate of ZIKV infection of 66%; Prevalence of 55% in Rio de Janeiro. infection in the 3rd trimester: Prevalence: 29% (Rio de Janeiro). In a series of 13 infants with congenital ZIKV infection and progressive microcephaly, more than half of the mothers did not report any symptoms prior to delivery.		In a study of 70 children with microcephaly and laboratory diagnosys of congenital ZIKV infection, 5 (7%) had sensorineural hearing loss.	One study: congenital heart disease was described in 14 of a series of 103 cases (13.6%) in children with CZS.
Santos et al. (2018) [28]		Intracranial calcifitcation: 23 of 23 childen. Frontal lobe: 69% - 78%. Parietal lobe: 83% - 87%. Corticomedullary junction: 53% - 86%. Thalamus: 39% - 43%. Punctate calcification: 72% - 100%. Distributed in the band format: 56% - 75%. Reduction in the constitution of gyri of the severe cerebral cortex: 0.78. Cerebellar hypoplasia: 0.74. Involving only one cerebellar hemisphere : 13%. Brainstem globally hypoplastic: 8.7%. Abnormal hypodensity of the white matter: 1. Diffuse involvement of all the cerebral lobes: 0.87. Basal ganglia calcification: 57% - 65%				
Pomar et al. (2019) [30]	CZS: 4-9% of pregnancies of women infected by ZIKV. Malformations of cortical development: 79-82% of CZS cases. Intraventicular synechiae and periventricular cystic degeneration: 58% of CZS cases. Malformations of the corpus callosum: 71-100%. Vermian hypoplasia: 42% of CZS cases. 21% to 82%. Swallowing disorders and hydramnios: 25%. Partial immobilization or arthrogryposes: 10-25%. Motor abnormalities : 77.3-100% of CZS cases. Adverse outcomes - No signs/complications: 45% of proven infected fetuses/newborn. Adverse outcomes - Mild / moderate signs: 20% of proven infected fetuses/newborn. Adverse outcomes - Severe complications: 21% of proven infected fetuses/newborn. Risk of neurodevelopmental abnormality: 9% of infants born from infected mothers	Brain volume loss: 92%. Ventriculomegaly in CZS: 63.1-92%. Calcifications in CZS: 71-92%	Prevalence of microcephaly in CZS: 33.3-64%	Eye abnormalities: 25% in infants with CZS		

Authors	Malformations / Congenital abnormalities	Brain abnormalities	Microcephaly	Ocular disorders	Auditory disorder - Rate	Cardiovascular damage
**Wilder-Smith et al. (2018) [31]**	From infected pregnant travelers: Fetuses or infants with birth defects: 6% for asymptomatic women and 6% for symptomatic women with evidence of possible recent ZIKV infection. Zika virus-associated birth defects in infants with ZIKV infection: 10% in completed pregnancies with reported outcomes; 5% in infants with possible ZIKV-associated birth defects from women with confirmed or probably ZIKV infection) (5% among symptomatic and 4% among asymptomatic women). Among 1,508 pregnancies with lab-confirmed ZIKV (5% among symptomatic and 7% among asymptomatic woman). Adverse fetal outcomes: 7% in pregnant women with symptomatic ZIKV infection. Adverse outcomes: 3 of 4 ZIKV infected pregnant women.					
**Nithiyanantham et al. (2019) [32]**	Prevalence of joint abnormalities: 13.2% in infants of ZIKV-infected mothers	In infants of ZIKV-infected mothers: Ventriculomegaly / hydrocephaly: 21.8% (95% CI,15.2-28.4); Brain calcifications: 42.6% (95% CI, 30.8-54.4)	Prevalence of 3.9% in infants of ZIKV-infected mothers	Prevalence: 4.2% in infants of ZIKV-infected mothers		
**Masel et al. (2019) [33]**	No association of prior exposure to DENV and fetal imaging abnormalities					
**Barbosa et al. (2019) [34]**	Microcephaly or neurologic changes: 50.10% on 962 fetus or children studied				Altered OAE varied from 0% to 75%, while altered a-ABR varied from 0% to 29.9%. Among patients who underwent OAE assessments (n=244), 18,4% presented alterations while 25% of microcephaly cases dislpayed alterations. Among the 448 patients who reportedly underwent the first a-ABR test, 15.2% presented alterations. Among three studies that included 102 children with laboratory confirmation of congenital ZIKV infection, 18 (17.6%) had hearing alterations, five in the ABR and 13 in the HINE.	
**Minhas et al. (2017) [35]**						Cohort with 9 adults positive for ZIKV and no previous cardiac history. 8 of the cases had arrhythmicas and 6 presented heart failure. Of the 8 arrhythmias, 3 were acute atrial fibrillation (two paroxysmal, one persistent), 2 were non-sustained atrial tachycardia, and 2 were ventricular arrhythmias. 5 of the 6 heart failure patients had a low ejection fraction (EF), and one had preserved EF with pre-eclampsia and moderate to severe pericardial effusion.

For peer review only - http://bmjopen.bmj.com/site/about/guidelines.xhtml

Supplementary file 2 - Table 2. Health outcomes - Neurological

Authors	Neurological complications	Epilepsy	Sleep characteristics	GBS
**Krauer et al. (2017) [15]**				74-84% symptomatic ZIKV in GBS cases; ZIKV laboratory-confirmed in GBS cases investigated: 100%
**Paixao et al. (2016) [16]**	French Polynesia outbreak: Amongg patients that visited health care facilities with Zika-like symptoms, 2.3 per 1,000 had neurological complications			In Bahia, Brazil, GBS was diagnosed in 1 of every 1,000 reported ZIKV cases. French Polynesia outbreak: Among patients that visited health care facilities with Zika-like symptoms, 1.3/1,000 ZIKV infections had GBS. ZIKV symptomatic cases when confirmed Among 42 GBS cases, 36% required intensive care and 21% required mechanical ventilation; El salvador: Prevalence of 35% (84 GBS cases in 240 ZIKV infections)
**Simoes et al. (2016) [19]**				In the primary databases consulted, there is only one case report occurred in French Polynesia in which GBS was diagnosed in a patient infected with Zika virus.
**Padilla et al. (2016) [20]**				An analysis of 42 patients who developed GBS during the French Polynesia outbreak estimates the incidence of the disease to be 0.24 per 1000 Zika virus infections. 88% of these patients reported symptoms and 93% of patients showed evidence of recent disease with ZIKV confirmed by the presence of IgM antibodies. Of these patients, 38% required admission to an intensive care unit and 29% required mechanical ventilation.
**Marques et al. (2019) [21]**		Prevalence of epilepsy: 42.2-67% in children with congenital ZIKV. Infantile spasms: 72%, 21.6%. Generalized: 11.8%. Partial: 8.9%. Described as brief jerking spells of flexion and/or extension movements that lasted a few seconds : 21.57%. Focal motor seizures: 21%. Tonic seizures: 4%. Myoclonic seizures: 2%. Myoclonic seizures: 1%.	34.1% (30 in 88 congenital ZIKV-infected children) were defined as poor sleepres and 24% (21 in 88) slept less than 9 hours	

For peer review only - http://bmjopen.bmj.com/site/about/guidelines.xhtml

Authors	Neurological complications	Epilepsy	Sleep characteristics	GBS
**Counotte et al. (2018) [22]**				Prevalence ratio during the ZIKV transmission over pre-outbreak period: 2.0-9.8.
**Haby et al. (2018) [23]**				Prevalence of asymptomatic ZIKV infection in patients with GBS: 0.12
**Wahid et al. (2018) [25]**	A recent study presented neurological disorders in 12 of 16 patients co-infected with ZIKV, chikungunya virus, and dengue virus in Guayaquil, Ecuador. One patients experienced CNS vasculitis, three had GBS whereas, and six patients were diagnosed with meningitis or encephalitis.			About 43% of GBS patients were found to be positive for ZIKV. Another study confirmed ZIKV-linked GBS in 1 of 3 patients.
**Barbi et al. (2018) [27]**				Meta-analysis: 1513 GBS cases in 164,651 ZIKV-infected individuals (0.92%). Estimative the prevalence of GBS to be 1.23% (CI: 95% 1.17%-1.29%) of all ZIKV infection cases in adults. 16 in 38 GBS cases (42%) needed intensive care unit hospitalization (French Polynesia)
**Wachira et al. (2018) [29]**				OR: 59.7 (CI: 95% 10.4 - ∞); Other study: no statistical significance between ZIKV and GBS
**Pomar et al. (2019) [30]**		9-95.5% in congenital ZIKV infection		Prevalence of 1.23% (95% CI, 1.17%-1.29%) in general ZIKV infected-population)
**Wilder-Smith et al. (2018) [31]**				2.15% (2 cases in 93 ZIKV cases recorded in Geosentinel sites)
**Masel et al. (2019) [33]**	No association of prior exposure to DENV and clinical neurological assessment of fetus			No statistically significant difference in patients with GBS with or without prior DENV exposure. No statistical difference in prior DENV exposed patients with or without GBS after ZIKV infection.

Supplementary file 2 - Table 3. Health outcomes – Adverse outcomes

Authors	Death due ZIKV infection	Abortion due to ZIKA / fetal death / perinatal death / neonatal death	Intrauterine growth restrictions - Rate within mother-infant pairs	Abnormal amniotic fluid	Adverse birth outcomes
**Krauer et al. (2017) [15]**		Prevalence in all pregnancy outcomes: Miscarriage 2.5%; intrauterine death or stillbirth 1.1%; termination of pregnancy 5.4%; Neonatal death: 3.2%	28.57% of cases	Rate: 18% of infected pregnant women	
**Paixão et al. (2016) [16]**	In Brazil, 2 deaths of adults were attribuited to Zika and 7 are under investigation by the Ministry of Health; El Salvador (240 ZIKV cases, 2 deaths)				
**Chibueze et al. (2017) [17]**					
**Coelho et al. (2017) [18]**		Miscarriages and perinatal deaths: USA (22% - 2 deaths in 9 ZIKV infected pregnant women), Brazil (6.7% - 9 deaths in 135 ZIKV infected pregnant women), Puerto Rico (3% - 2 deaths in 67 ZIKV infected pregnant women), USA (10.6% - 47 deaths in 442 ZIKV infected pregnant women), French Guiana (4% - 20 deaths in 498 ZIKV infected pregnant women).			
**Simões et al. (2016) [19]**		In Brazil, 1.79% (91/5,079) of microcephaly reported cases, progressed to miscarriage or postpartum death. According to the classification, 64.8% (59/91) remained under investigation; 838% (8/91) were investigated and discarded, and 26.4% (24/91) were investigated and confirmed for microcephaly and/or changes in the CNS.			
**Padilla et al. (2016) [20]**		In 72 women with Zika-positive serology during pregnancy in Brazil, the fetal death rate was 4.8%; Zika infection during 9 pregnancies confirmed by CDC resulted in outcomes of 2 spontaneous abortions and 2 elective abortions.			
**Wahid et al. (2018) [25]**			One study with 88 pregnant women of which 72 were positive for ZIKV and ultrasonography was performed in 42: in utero growth restriction with or without microcephaly (5/42).		
**Pomar et al. (2019) [30]**		14% of proven infected fetuses/newborn	Prevalence of IUGR in CZS: 14%		
**Masel et al. (2019) [33]**		No association of prior exposure to DENV and fetal loss			Occured in 46.4% of those ZIKV infected participants

For peer review only

Supplementary file 3 - Table 1.1. Summary of AMSTAR 2 rating

AMSTAR 2	Krauer et al. [15]	Paixão et al. [16]	Chibueze et al. [17]	Coelho et al. [18]	Simões et al. [19]	Padilla et al. [20]	Marques et al. [21]
**1**	Yes	Yes	Yes	Yes	Yes	No	Yes
**2**	No	No	Partial yes	No	No	No	Yes
**3**	Yes	Yes	Yes	Yes	Yes	Yes	Yes
**4**	Partial yes	Yes	Partial yes	Partial yes	Partial yes	Yes	Partial yes
**5**	Yes	Yes	Yes	Yes	Yes	Yes	Yes
**6**	Yes	No	Yes	Yes	No	No	No
**7**	Yes	Yes	Yes	Yes	No	No	No
**8**	Partial yes	Partial yes	Yes	Partial yes	No	No	No
**9**	No	No	No	No	No	No	No
**10**	Yes	No	Yes	Yes	No	No	Yes
**11**	No	No	No	No	No	No	No
**12**	Yes	No MA conducted	No MA conducted	Yes	No MA conducted	No MA conducted	No MA conducted
**13**	No	No MA conducted	No MA conducted	No	No MA conducted	No MA conducted	No MA conducted
**14**	No	No	No	No	No	No	No
**15**	Yes	Yes	No	Yes	No	No	No
**16**	No	No MA conducted	No MA conducted	No	No MA conducted	No MA conducted	No MA conducted

*MA - Meta-analysis

Questions 2,4,7,9,12 and 14, highlighted, are those of critical domains.

1 - Did the research questions and inclusion criteria for the review include the components of PICO?

2 - Did the report of the review contain an explicit statement that the review methods were established prior to the conduct of the review and did the report justify any significant deviations from the protocol?

3 - Did the review authors explain their selection of the study designs for inclusion in the review?

4 - Did the review authors use a comprehensive literature search strategy?

5 - Did the review authors perform study selection in duplicate?

6 - Did the review authors perform data extraction in duplicate?

7 - Did the review authors provide a list of excluded studies and justify the exclusions?

8 - Did the review authors describe the included studies in adequate detail?

9 - Did the review authors use a satisfactory technique for assessing the risk of bias (RoB) in individual studies that were included in the review?

10 - Did the review authors report on the sources of funding for the studies included in the review?

11 - If meta-analysis was performed did the review authors use appropriate methods for statistical combination of results?

12 - If meta-analysis was performed, did the review authors assess the potential impact of RoB in individual studies on the results of the meta-analysis or other evidence synthesis?

13 - Did the review authors account for RoB in individual studies when interpreting/ discussing the results of the review?

14 - Did the review authors provide a satisfactory explanation for, and discussion of, any heterogeneity observed in the results of the review?

15 - If they performed quantitative synthesis did the review authors carry out an adequate investigation of publication bias (small study bias) and discuss its likely impact on the results of the review?

16 - Did the review authors report any potential sources of conflict of interest, including any funding they received for conducting the review?

For peer review only

Supplementary file 3 - Table 1.2. Summary of AMSTAR 2 rating

AMSTAR 2	Counotte et al. [22]	Haby et al. [23]	Sarwar et al. [24]	Wahid et al. [25]	Soriano-Arandes et al. [26]	Barbi et al. [27]	Santos et al. [28]
**1**	Yes	Yes	Yes	Yes	Yes	Yes	Yes
**2**	Yes	Partial yes	No	No	No	No	No
**3**	No	Yes	No	No	No	No	No
**4**	Partial yes	Partial yes	Partial yes	No	No	No	No
**5**	Yes	No	No	Yes	No	No	No
**6**	Yes	No	No	No	No	Yes	No
**7**	No	Yes	No	No	No	No	No
**8**	Yes	Yes	No	Yes	Yes	Yes	No
**9**	No	No MA conducted	No MA conducted	No MA conducted	No MA conducted	No MA conducted	No
**10**	No	Yes	No	No	No	Yes	No
**11**	No	No	No	No	Yes	No	No
**12**	No MA conducted	Yes	No MA conducted	No MA conducted	No MA conducted	No	No MA conducted
**13**	No MA conducted	No	No MA conducted	No MA conducted	No MA conducted	No	No MA conducted
**14**	No	Yes	No	No	No	No	Yes
**15**	No	Yes	No	No	No	No	No
**16**	No MA conducted	Yes	No MA conducted	No MA conducted	No MA conducted	No	No MA conducted

Questions 2,4,7,9,12 and 14, highlighted, are those of critical domains.
1 - Did the research questions and inclusion criteria for the review include the components of PICO?
2 - Did the report of the review contain an explicit statement that the review methods were established prior to the conduct of the review and did the report justify any significant deviations from the protocol?
3 - Did the review authors explain their selection of the study designs for inclusion in the review?
4 - Did the review authors use a comprehensive literature search strategy?
5 - Did the review authors perform study selection in duplicate?
6 - Did the review authors perform data extraction in duplicate?
7 - Did the review authors provide a list of excluded studies and justify the exclusions?
8 - Did the review authors describe the included studies in adequate detail?
9 - Did the review authors use a satisfactory technique for assessing the risk of bias (RoB) in individual studies that were included in the review?
10 - Did the review authors report on the sources of funding for the studies included in the review?
11 - If meta-analysis was performed did the review authors use appropriate methods for statistical combination of results?
12 - If meta-analysis was performed, did the review authors assess the potential impact of RoB in individual studies on the results of the meta-analysis or other evidence synthesis?
13 - Did the review authors account for RoB in individual studies when interpreting/ discussing the results of the review?
14 - Did the review authors provide a satisfactory explanation for, and discussion of, any heterogeneity observed in the results of the review?
15 - If they performed quantitative synthesis did the review authors carry out an adequate investigation of publication bias (small study bias) and discuss its likely impact on the results of the review?
16 - Did the review authors report any potential sources of conflict of interest, including any funding they received for conducting the review?

For peer review only

Supplementary file 3 - Table 1.3. Summary of AMSTAR 2 rating

AMSTAR 2	Wachira et al. [29]	Pomar et al. [30]	Wilder-Smith et al. [31]	Nithiyanantham et al. [32]	Masel et al. [33]	Barbosa et al. [34]	Minhas et al. [35]
**1**	Yes	Yes	Yes	Yes	Yes	Yes	Yes
**2**	Yes	No	No	No	No	Yes	No
**3**	Yes	No	Yes	Yes	Yes	Yes	Yes
**4**	Partial yes	No	Partial yes	Partial yes	Yes	Partial yes	Partial yes
**5**	Yes	Yes	No	Yes	Yes	Yes	Yes
**6**	Yes	Yes	No	No	Yes	Yes	Yes
**7**	No	No	No	No	No	No	No
**8**	Yes	No	Partial yes	Yes	Yes	Yes	Yes
**9**	No	No	No	No	No	Yes	No
**10**	Yes	No	No	No	No	No	No
**11**	No	No MA conducted	No MA conducted	No	No MA conducted	No MA conducted	No MA conducted
**12**	No MA conducted	No MA conducted	No MA conducted	No	No MA conducted	No MA conducted	No MA conducted
**13**	No MA conducted	No	No	No	No	No	No
**14**	Yes	No	Yes	Yes	No	Yes	No
**15**	Yes	No MA conducted	No MA conducted	Yes	No MA conducted	No MA conducted	No MA conducted
**16**	No MA conducted	Yes	Yes	Yes	Yes	Yes	Yes

Questions 2,4,7,9,12 and 14, highlighted, are those of critical domains.
1 - Did the research questions and inclusion criteria for the review include the components of PICO?
2 - Did the report of the review contain an explicit statement that the review methods were established prior to the conduct of the review and did the report justify any significant deviations from the protocol?
3 - Did the review authors explain their selection of the study designs for inclusion in the review?
4 - Did the review authors use a comprehensive literature search strategy?
5 - Did the review authors perform study selection in duplicate?
6 - Did the review authors perform data extraction in duplicate?
7 - Did the review authors provide a list of excluded studies and justify the exclusions?
8 - Did the review authors describe the included studies in adequate detail?
9 - Did the review authors use a satisfactory technique for assessing the risk of bias (RoB) in individual studies that were included in the review?
10 - Did the review authors report on the sources of funding for the studies included in the review?
11 - If meta-analysis was performed did the review authors use appropriate methods for statistical combination of results?
12 - If meta-analysis was performed, did the review authors assess the potential impact of RoB in individual studies on the results of the meta-analysis or other evidence synthesis?
13 - Did the review authors account for RoB in individual studies when interpreting/ discussing the results of the review?
14 - Did the review authors provide a satisfactory explanation for, and discussion of, any heterogeneity observed in the results of the review?
15 - If they performed quantitative synthesis did the review authors carry out an adequate investigation of publication bias (small study bias) and discuss its likely impact on the results of the review?
16 - Did the review authors report any potential sources of conflict of interest, including any funding they received for conducting the review?

For peer review only

PRISMA 2009 Checklist

Section/topic	#	Checklist item	Reported on page #
**TITLE**			
Title	1	Identify the report as a systematic review, meta-analysis, or both.	1
**ABSTRACT**			
Structured summary	2	Provide a structured summary including, as applicable: background; objectives; data sources; study eligibility criteria, participants, and interventions; study appraisal and synthesis methods; results; limitations; conclusions and implications of key findings; systematic review registration number.	2
**INTRODUCTION**			
Rationale	3	Describe the rationale for the review in the context of what is already known.	3
Objectives	4	Provide an explicit statement of questions being addressed with reference to participants, interventions, comparisons, outcomes, and study design (PICOS).	3
**METHODS**			
Protocol and registration	5	Indicate if a review protocol exists, if and where it can be accessed (e.g., Web address), and, if available, provide registration information including registration number.	3,4
Eligibility criteria	6	Specify study characteristics (e.g., PICOS, length of follow-up) and report characteristics (e.g., years considered, language, publication status) used as criteria for eligibility, giving rationale.	3,4
Information sources	7	Describe all information sources (e.g., databases with dates of coverage, contact with study authors to identify additional studies) in the search and date last searched.	3,4
Search	8	Present full electronic search strategy for at least one database, including any limits used, such that it could be repeated.	3
Study selection	9	State the process for selecting studies (i.e., screening, eligibility, included in systematic review, and, if applicable, included in the meta-analysis).	3,4
Data collection process	10	Describe method of data extraction from reports (e.g., piloted forms, independently, in duplicate) and any processes for obtaining and confirming data from investigators.	3,4
Data items	11	List and define all variables for which data were sought (e.g., PICOS, funding sources) and any assumptions and simplifications made.	
Risk of bias in individual studies	12	Describe methods used for assessing risk of bias of individual studies (including specification of whether this was done at the study or outcome level), and how this information is to be used in any data synthesis.	4
Summary measures	13	State the principal summary measures (e.g., risk ratio, difference in means).	5-6
Synthesis of results	14	Describe the methods of handling data and combining results of studies, if done, including measures of consistency (e.g., I^2) for each meta-analysis.	

For peer review only - http://bmjopen.bmj.com/site/about/guidelines.xhtml

PRISMA 2009 Checklist

Section/topic	#	Checklist item	Reported on page #
Risk of bias across studies	15	Specify any assessment of risk of bias that may affect the cumulative evidence (e.g., publication bias, selective reporting within studies).	
Additional analyses	16	Describe methods of additional analyses (e.g., sensitivity or subgroup analyses, meta-regression), if done, indicating which were pre-specified.	
**RESULTS**			
Study selection	17	Give numbers of studies screened, assessed for eligibility, and included in the review, with reasons for exclusions at each stage, ideally with a flow diagram.	4
Study characteristics	18	For each study, present characteristics for which data were extracted (e.g., study size, PICOS, follow-up period) and provide the citations.	5
Risk of bias within studies	19	Present data on risk of bias of each study and, if available, any outcome level assessment (see item 12).	5
Results of individual studies	20	For all outcomes considered (benefits or harms), present, for each study: (a) simple summary data for each intervention group (b) effect estimates and confidence intervals, ideally with a forest plot.	
Synthesis of results	21	Present results of each meta-analysis done, including confidence intervals and measures of consistency.	
Risk of bias across studies	22	Present results of any assessment of risk of bias across studies (see Item 15).	
Additional analysis	23	Give results of additional analyses, if done (e.g., sensitivity or subgroup analyses, meta-regression [see Item 16]).	
**DISCUSSION**			
Summary of evidence	24	Summarize the main findings including the strength of evidence for each main outcome; consider their relevance to key groups (e.g., healthcare providers, users, and policy makers).	6-8
Limitations	25	Discuss limitations at study and outcome level (e.g., risk of bias), and at review-level (e.g., incomplete retrieval of identified research, reporting bias).	8
Conclusions	26	Provide a general interpretation of the results in the context of other evidence, and implications for future research.	9
**FUNDING**			
Funding	27	Describe sources of funding for the systematic review and other support (e.g., supply of data); role of funders for the systematic review.	9

From: Moher D, Liberati A, Tetzlaff J, Altman DG, The PRISMA Group (2009). Preferred Reporting Items for Systematic Reviews and Meta-Analyses: The PRISMA Statement. PLoS Med 6(7): e1000097. doi:10.1371/journal.pmed1000097

For more information, visit: **www.prisma-statement.org**.

For peer review only - http://bmjopen.bmj.com/site/about/guidelines.xhtml

Health outcomes associated with Zika virus infection in humans: a systematic review of systematic reviews

Journal:	*BMJ Open*
Manuscript ID	bmjopen-2019-032275.R3
Article Type:	Original research
Date Submitted by the Author:	25-Sep-2019
Complete List of Authors:	Ximenes, Raphael; University Health Network, Toronto Health Economics and Technology Assessment (THETA) Collaborative, University Health Network<br>Ramsay, Lauren; University Health Network, Toronto Health Economics and Technology Assessment (THETA) Collaborative, University Health Network; University of Toronto, Institute of Health Policy, Management and Evaluation, University of Toronto<br>Miranda, Rafael; University Health Network, University Health Network, Toronto Health Economics and Technology Assessment (THETA) Collaborative, University Health Network<br>Morris, Shaun; Hospital fo sick children, Division of Infectious Diseases and Centre for Global Child Health, Hospital for Sick Children; University of Toronto Faculty of Medicine, Department of Pediatrics, Faculty of Medicine, University of Toronto<br>Murphy, Kellie; Mount Sinai Hospital<br>Sander, Beate; University Health Network, Toronto Health Economics and Technology Assessment (THETA) Collaborative, University Health Network; University of Toronto, Institute of Health Policy, Management and Evaluation, Management and Evaluation, University of Toronto
**Primary Subject Heading**:	Infectious diseases
Secondary Subject Heading:	Global health, Epidemiology, Obstetrics and gynaecology, Paediatrics, Neurology
Keywords:	Zika Virus infection, Epidemiology < INFECTIOUS DISEASES, Congenital Zika Syndrome, NEUROLOGY, Guillain-Barré syndrome, Microcephaly

SCHOLARONE™
Manuscripts

Health outcomes associated with Zika virus infection in humans: a systematic review of systematic reviews

Raphael Ximenes[1], Lauren C. Ramsay[1,2], Rafael N. Miranda[1], Shaun K. Morris[3,4], Kellie E. Murphy[5], RADAM-LAC Research Team, Beate Sander[1,2,6,7]

[1] Toronto Health Economics and Technology Assessment (THETA) Collaborative, University Health Network, Toronto, ON, Canada, raphael.ximenes@theta.utoronto.ca.

[2] Institute of Health Policy, Management and Evaluation, University of Toronto, Toronto, ON, Canada.

[3] Division of Infectious Diseases and Centre for Global Child Health, Hospital for Sick Children, Toronto, ON, Canada.

[4] Department of Pediatrics, Faculty of Medicine, University of Toronto, Toronto, ON, Canada.

[5] Mount Sinai Hospital, Toronto, ON, Canada.

[6] Public Health Ontario, Toronto, ON, Canada.

[7] ICES, Toronto, ON, Canada.

RADAM-LAC Research Team members are Beate Sander, Camila Gonzalez, Manisha Kulkarni, Marcos Miretti, Mauricio Espinel, Jianhong Wu and Varsovia Cevallos.

For peer review only

Abstract

Objective: With the emergence of Zika virus (ZIKV) disease in Central and South America in the mid-2010s and recognition of the teratogenic effects of congenital exposure to ZIKV, there has been a substantial increase in new research published on ZIKV. Our objective is to synthesize the literature on health outcomes associated with ZIKV infection in humans.

Methods: We conducted a systematic review (SR) of SRs following PRISMA (Preferred Reporting Items for Systematic Reviews and Meta-Analyses) guidelines. We searched MEDLINE, Embase, Cochrane and LILACS databases from inception to July 22, 2019, and included SRs that reported ZIKV associated health outcomes. Three independent reviewers selected eligible studies, extracted data and assessed the quality of included SRs using the A MeaSurement Tool to Assess Systematic Reviews 2 (AMSTAR 2) tool. Conflicts were resolved by consensus or consultation with a third reviewer.

Results: The search yielded 1,382 unique articles, of which 21 SRs met our inclusion criteria. The 21 SRs ranged from descriptive to quantitative data synthesis, including four meta-analysis. The most commonly reported ZIKV-associated manifestations and health outcomes were microcephaly, congenital abnormalities, brain abnormalities, neonatal death, and Guillain-Barré syndrome. The included reviews were highly heterogeneous. The overall quality of the SRs was critically low with all studies having more than one critical weakness.

Conclusion: The evolving nature of the literature on ZIKV-associated health outcomes, together with the critically low quality of existing SRs, demonstrate the need for high-quality SRs to guide patient care and inform policy decision making.

Strengths and limitations:

- Lack of SRs on ZIKV in the literature
- Lack of information about the risks of severe outcomes related to ZIKV infection or the presence of specific outcomes
- Broad search strategy
- Without restrictions by language or publication type
- To our knowledge, this is the first SR of SRs about health outcomes associated with ZIKV infection in humans

For peer review only

Introduction

Zika Virus (ZIKV) was first discovered in 1947 in rhesus monkeys in Uganda (1). It is an arbovirus in the flavivirus family and typically causes mild illness in humans characterized by fever and rash. There were reports of sporadic cases of ZIKV infection in humans over the years in Asia and Africa (2), with the first large documented outbreak taking place in Yap, a Micronesian island, in 2007 (3). Since then, there have been reported outbreaks in French Polynesia (in 2013-2014), and most recently in South and Central America and the Caribbean (4). With the emergence of ZIKV in Brazil, there were over 800,000 estimated cases of ZIKV infection reported by countries and territories in the Americas by January 2018 (5). By March 2017, according to the latest World Health Organization (WHO) global situation report on Zika, 84 countries, territories or subnational areas had evidence of vector-borne ZIKV transmission (6). According to the CDC, until May 2019, there were 89 areas with current or past transmission, but no current outbreak of ZIKV (7).

Our understanding of Zika-associated clinical outcomes has evolved over time. Before human pathogenesis was understood, cellular level damage was apparent in animal studies in the 1950s (8). The first study in humans to suggest an association between ZIKV and human disease was a case-control study during an outbreak in French Polynesia between 2013 and 2014, suggesting an association with Guillain-Barre Syndrome (GBS). (9). However, the link between ZIKV in pregnant women and microcephaly in infants was only evident in the 2015-2016 outbreak in South America (10). With the spread of ZIKV to new regions of the world and the extent of the outbreak in South and Central American and Caribbean countries, a substantial body of new research has been published in recent years about Zika.

A bibliometric analysis of ZIKV research that indexed in Web of Science found a significant increase in the number of studies being published beginning in 2015 (n=38 publications) to 2017 (n=1,962 publications) (11). Summarizing the large body of literature on outcomes associated with ZIKV infection is timely and needed.

The purpose of this systematic review (SR) of systematic reviews was to synthesize the currently known health outcomes associated with ZIKV infection in humans.

Methods

Search strategy and selection criteria

We searched MEDLINE, Embase, Cochrane and LILACS databases from inception to July 22, 2019. Our search strategy across all databases included concepts related to “Zika” and “systematic review” (complete search strategy found in Supplementary File 1). Our search strategy was not restricted by language or publication type. Three reviewers (RX, first reviewer; LR and RM second reviewers) independently screened titles, abstracts, and relevant full text of identified articles.

For peer review only

The inclusion criteria were defined as SRs that reported health outcomes of ZIKV infection in humans, i.e. clinical presentation and sequelae of ZIKV infection in humans. We excluded studies that only reported symptoms (e.g., rash, fever) of ZIKV infection, diagnostic techniques, mosquito control, therapeutic regimes, vaccine and trial but not outcomes (e.g., GBS, Congenital Zika Syndrome). We followed the Preferred Reporting Items for Systematic Reviews and Meta-Analyses (PRISMA) guidelines for reporting results (12).

The data extraction was performed in duplicate by the reviewers. The SR methods were established prior to the conduct of the SR and the protocol for the current SR was registered with PROSPERO (CRD42018091087) and there were no deviations from the protocol, except for adding the LILACS database to the search.

Patient and Public Involvement

No patient involved.

Quality appraisal

We used the A MeaSurement Tool to Assess systematic Reviews 2 (AMSTAR 2) tool to critically appraise the included SRs (13). AMSTAR 2 is not intended to generate an overall score, but rather to assist in the identification of high-quality SRs. Three reviewers (RX, first reviewer; LR and RM, second reviewers) independently evaluated the quality of each study based on weaknesses in critical domains as defined by the AMSTAR 2 tool. Studies were rated based on the overall confidence in the results of the SR and defined as either high (zero or one non-critical weakness), moderate (more than one non-critical weakness), low (one critical flaw with or without non-critical weaknesses) or critically low (more than one critical flaw with or without non-critical weaknesses) (14). Critical domains included protocol registration, adequacy of the literature search, justification for excluding studies, risk of bias from individual studies included in the SR, appropriateness of meta-analytical methods, consideration of risk of bias when interpreting results, and assessment of publication bias (14). Any disagreements between the two reviewers were resolved by consensus.

Data analysis

Three reviewers (RX, first reviewer; LR and RM, second reviewers) extracted the data using a structured electronic data extraction form, extracting study characteristics, and measures of effect for outcomes resulting from ZIKV infection. Included studies were summarized narratively, and health outcomes were reported where possible.

For peer review only

Results

We identified 1,382 unique articles from the database searches (Figure 1). After screening titles and abstracts, we selected 85 for full text screening. Of these, twenty-one met our inclusion criteria (15–35). The main reasons for exclusion at the full text stage were articles not being SRs (but rather overviews or literature reviews) and studies only reported symptoms but not outcomes.

Study characteristics are summarized in Table 1. The included SRs were published between February 2016 and May 2019. The types of studies eligible for inclusion in the SRs varied across studies; four SR did not include any information on the included studies (21,24,28,30), all other SRs included observational studies (one limited to only cohort studies (18)), and the majority (71%; n=15) included case reports and case series. Three SRs considered evidence from modelling studies, animal experiments, and in vitro experiments (15,33,35). Another did not limit to reports of primary data and included SRs, narrative reviews, and news articles (20).

The majority of studies included in the SRs were conducted in Brazil, the United States (US), French Polynesia and Colombia.

Summary of included SRs and outcomes

Of the 21 included SRs, the most commonly reported outcome was microcephaly, reported in 14 SRs (15–26,30,32), 12 SRs reported on GBS (15,16,19,20,22,23,25,27,29–31,33), 11 SRs reported on malformations or congenital abnormalities (18–20,22,26,30–34), 9 reported on brain (15,17,21,24–26,28,30,32), 7 SRs reported on ocular disorders (15,18,21,24,25,30,32), and 6 SRs on termination of pregnancy, fetal death and perinatal death (15,18–20,30,33). Three SRs or fewer reported on auditory disorder (15,26,34), cardiovascular damage (18,26,35), neurological complications (16,25,33), intrauterine growth restrictions (15,25), abnormal amniotic fluid (15), epilepsy (21), and death due Zika infection (16).

Seven SRs focused on pregnant women (17–20,24,26,28) and 5 SRs included the general population (15,16,22,23,29), while newborns, neonates, perinatal, early birth or infants were included in 5 five SRs (18,19,21,25,26). One SR focused in travelers returning to the US and Europe (31). Adults were the included in two of the 15 SRs (25,27).

Overlap between systematic reviews

Our SR includes 21 SRs. The overlap between the results of the 21 SRs included 860 studies (Table 1), 615 of which were not duplicates. Out of the 615 studies, 477 (77.56%) were cited only once as studies included in the SRs included in our SR, and the remainder were cited in up to 10 SRs, 83 (13.50%) were cited twice, 29 (4.72%) three times, 8 (1.30%) four times, 8 (1.30%) five times, 6 (0.98%) six times, 2 (0.33%) seven times, one (0.16%) eight times and one (0.16%) ten times (3,36–52) (Table 2, Figure 2).

For peer review only

Health Outcomes

The Supplementary File 2 reports the health outcome data extracted from the twenty-one SRs.

Clinical Outcomes Associated with ZIKV Infection During Pregnancy

The Supplemental File 2 shows that the reported outcomes associated with ZIKV infection during pregnancy ranging from adverse birth outcomes to perinatal death. The frequency of infant deaths (miscarriages, perinatal deaths, intrauterine death or stillbirth and termination of pregnancy) was reported by 6 of 21 SRs (15,18–20,30,33), ranging from 4.8% to 22%.

Congenital Zika syndrome (CZS) was reported in many different ways. Some studies reported specific outcomes related to CZS (e.g. brain abnormalities, ocular disorder or microcephaly) while others reported CZS as a nonspecific outcome. The prevalence of CZS ranged from 2% (5 cases in 250 ZIKV-infected pregnant women) (18) to 50% (58 adverse congenital outcomes out of 117 women with PCR confirmed ZIKV) (22).

Brain abnormalities were explicitly reported with data from 19 studies in which 96% (205 in 213 pregnant women) of fetuses were diagnosed after confirmation with imaging tests (15). One SR reported the prevalence of brain abnormalities (28%) including microcephaly in newborns whose mothers were infected with ZIKV in pregnancy (25) while other SR reported an observational study of 35 infants with microcephaly, 11 fetuses had intra-uterine brain injury accompanied by stunting of cerebral growth prior to birth (17). Further, five SRs classified the type of brain abnormalities or where the lesions were found (21,24,28,30,32)as intracranial calcification, reduction in the constitution of gyri of the severe cerebral cortex, abnormal hypodensity of the white matter, malformations of cortical development, subcortical-cortical junction calcifications, basal ganglia calcification, brain calcification, intraventicular synechiae and periventricular cystic, brain volume loss, ventriculomegaly / hydrocephaly and diffuse involvement of all the cerebral lobes.

Microcephaly was reported in 14 of 21 SRs. Chibueze et al. (2016) provided a trimester-specific modeling estimate risk for microcephaly. When the infection occurs in an indeterminate period of pregnancy, ZIKV associated microcephaly was described by Coelho et al. (2017). The authors performed a meta-analysis and found a prevalence of 2.3% (95% CI 1% - 5.3%) of microcephaly when considering all pregnancies (2,941 mother-infant pairs). When considering only live births (2,648 live births), the prevalence of microcephaly was 2.7% (95% CI 1.2% - 6%) (18). Nithiyanantham et al. (2019) also performed a meta-analysis of the proportion of congenital disorders in infants born to ZIKV-infected mothers, reporting a prevalence of 3.9% (95% CI 2.4% – 5.4%) (32). Pomar et al. (2019) reported the prevalence of microcephaly in CZS ranging from 33.3% to 64% (30). Four SRs reported microcephaly cases per live-birth pregnancies, ranging from 0.2% (20 cases per 10,000 live births) to 14.3% (1 case in 7 live-birth pregnancies) (15,16,18,20) and one SRs reported 10 microcephaly cases per 10,000 births (19).

For peer review only

Microcephaly risk in infected pregnant women was reported in four SRs. The absolute risk varied between 0.95% (95% CI: 0.34 – 1.91%) during the first trimester of pregnancy to 30% (22,24–26) (trimester not reported). Death caused by microcephaly was estimated in a study reported by Coelho et al. (2017), reporting a rate of 8.3% (171 deaths among 2,063 confirmed cases of microcephaly) (18). The prevalence of microcephaly in asymptomatic ZIKV infection was also reported as 0.36% (0.22% – 0.51%) (23). Another SR reported that in a series of 13 infants with congenital ZIKV infection and microcephaly, more than half of the mothers did not report any symptoms of ZIKV prior to delivery (26).

The prevalence of congenital ZIKV syndrome-related outcomes is still unknown. In this SR of SRs we found the intrauterine growth restrictions rate reported varied from 28.57% (10 cases in 35 mother-infant pairs) (15) to 31.43%, from one observational study of 35 infants with microcephaly (17). Another study reported intrauterine growth restriction in 11.9% of fetuses with or without microcephaly (5 fetuses from 42 positives for ZIKV pregnant women) (25). Pomar et al. (2019) reported the prevalence of intrauterine growth restriction in 14% of CZS cases. The prevalence of ocular disorder was reported in five SRs ranging from 0.9% % (from one study with 395 live-birth pregnancies) to 58.6% (17 ocular findings with microcephaly associated in 29 infants) (15,18,21,24,25,30,32). Abnormal amniotic fluid was described only by Krauer et al. (2017). Auditory disorder was described by Krauer et al. (2018) (prevalence of 13% - 3 cases in 24 mother-infant pairs) and Soriano-Arandes et al. (2018) (prevalence of 7% - 5 cases in 70 children with laboratory diagnosis of ZIKV infection) and Barbosa et al. (2019) (variations in the frequency of altered otoacoustic emissions testing (OAE) and automated auditory brainstem (ABR) response testing across the studies in 515 children: altered OAE varied from 0% to 75%, while altered a-ABR varied from 0% to 29.2%). The prevalence of cardiovascular damage was reported by Coelho et al. (2017) (prevalence of 1% - 3 cases in 301 live-birth pregnancies), Soriano-Arandes et al. (2018) (prevalence of 13.6% - 14 cases in103 ZIKV cases) and Minhas et al. (2017) (prevalence of 67% of heart failure in a cohort with 9 adults positive for ZIKV and no previous cardiac history).

Neurological Complications Associated with ZIKV Infection

Neurological complications were reported by 12 of 21 SRs (16,19–23,25,27,29–31,33), where GBS was the most commonly reported neurological complication.

Among adults, the proportion of neurological complications associated with ZIKV infection in Bahia (Brazil) was similar to that in French Polynesia. Among these neurological complications, GBS was diagnosed in 1 of every 1,000 reported Zika cases in Brazil and 1.3 per 1,000 in French Polynesia (16). During the French Polynesia outbreak in 2013, the incidence of GBS has been 0.24 per 1,000 ZIKV infections (20), and Simões et al. (2016) described one case report in French Polynesia in which GBS was diagnosed in a patient with ZIKV (19).

Counotte et al. (2018) reported the increased incidence of GBS incidence ratio between during and pre-ZIKV outbreak periods in seven different countries; which ranged from 2.0 (95% CI: 1.6-2.6) to 9.8 (95% CI: 7.6-12.5), while Barbi et al. (2018) conducted a meta-analysis of the prevalence of GBS in

ZIKV infected cases. Their estimate for the prevalence of GBS in adults infected with ZIKV was 1.23% (CI: 95% 1.17%-1.29%). This same study was reported by Pomar et al. (2019). Krauer et al. (2017) reported the prevalence of symptomatic ZIKV in GBS cases (74-84% symptomatic ZIKV in GBS cases). Paixão et al. (2016), Padilla et al. (2016) and Barbi et al. (2018), described the prevalence of admission to an intensive care unit (ranging from 36% to 42%, among 42 and 38 GBS cases respectively) and mechanical ventilation (21% to 29% among 42 GBS cases) in French Polynesia. The interval between ZIKV and GBS symptoms was described by Krauer et al. (2017), Paixão et al. (2016), Padilla et al. (2016) and Counotte et al. (2018). The highest interval was reported by Paixão et al. (2016), where 88% of GBS cases reported a viral syndrome up to 23 days before the onset of the neurologic syndrome. No deaths due to GBS related with ZIKV infections were reported in this SR.

Epilepsy and sleep profiles were described in two SRs. For Marques et al. (2019), the prevalence of epilepsy in congenital ZIKV infants ranged from 42% (43 in 102 children with congenital ZIKV) to 67% (95 in 141 congenital ZIKV), and 34% (30 in 88 congenital ZIKV-infected children) of the ZIKV infected children were defined as poor sleepers (21). Pomar et al. (2019) reported that 9% to 95.5% of congenital ZIKV infections were associated with epilepsy.

Idiopatic thrombocytopaenia purpura (ITP) related with ZIKV infection was reported by Counotte et al. (2018). They reported 11 cases of ITP across 18 studies; however, there is no information about the total number of ZIKV infected subjects in these studies.

Deaths Associated with ZIKV Infection

Deaths due to Zika infection are rare. According to the Brazilian Ministry of Health, between 440,000 and 1,300,000 cases of Zika occurred in Brazil in 2015 (53,54). Since the beginning of the outbreak 11 deaths among adults were confirmed in Brazil and an additional nine deaths were reported by the countries and territories in the Americas (5).

Coinfection

Coinfection was reported with dengue (16–18,25), chikungunya (16,17,25) and HIV (16,17); cytomegalovirus, toxoplasmosis, or other known teratogenic agents (16–18); hepatitis B virus (HBV), hepatitis C virus (HCV), cytomegalovirus (CMV), herpes simplex virus (HSV), Epstein-Barr virus (EBV), rubella, human T lymphotrophic virus (HTLV), parvovirus B19 and syphilis (17).

Masel et al. (2019) found no association of prior exposure to DENV and fetal loss, or clinical neurological assessment of fetus, and no statistical difference in prior DENV exposed patients with or without GBS after ZIKV infection.

Quality assessment

Of the twenty-one SRs included, there was high inter-rater reliability between the reviewers (91%). The overall quality of the SRs was critically low with all studies identified as having more than one critical weakness with or without non-critical weaknesses (Figure 3). For all studies, the majority (65%)

For peer review only - http://bmjopen.bmj.com/site/about/guidelines.xhtml

of answers for the six critical domains of AMSTAR 2 tool (questions 2, 4, 7, 9, 12 and 14) were 'no' or 'partial yes' (53% and 12% respectively) (Figure 4 and Supplementary File 3). Main weaknesses identified were a deficient bibliographic search strategy and the lack of an explicit statement that SR methods were established prior to the conduct of the SR.

Discussion

Our SR of SRs identified 21 SRs that reported health outcomes associated with ZIKV infection. Microcephaly was the most commonly reported health outcome. Other outcomes reported were fetal death, neonatal death, congenital abnormalities including brain abnormalities, intrauterine growth restrictions, ocular disorders, and infant disorders including auditory disorders, cardiovascular damage, death due ZIKV infection, neurological complications, epilepsy and finally adult outcomes including GBS. The included SRs indicate that ZIKV infection is causally associated with congenital abnormalities, including microcephaly, and that ZIKV infection is a trigger of GBS, considering evidence on biological plausibility, the strength of association, and the exclusion of alternative explanations.

Overall, we found high heterogeneity among the twenty-one included SRs ranging from descriptive SRs, with few data on health outcomes associated with ZIKV infection, to more quantitative SRs, including four meta-analyses. There was some overlap (22%) of included studies across the SRs, indicating that the SRs are relatively distinct from each other and consistent with the included SRs reporting on different aspects of ZIKV infection. Given this heterogeneity it was not possible to perform a quantitative synthesis, making it difficult to compare the results or draw conclusions based on the included SRs. Further, our quality appraisal found that all SRs were of critically low quality, with only three or fewer of six critical domains of AMSTAR 2 tool met in any study.

Further research into the magnitude of effects, potential other immediate and late outcomes, and long-term sequelae is warranted to understand the full impact of ZIKV infection, particularly long-term follow up studies of infants born to ZIKV-infected mothers and infants and children infected with ZIKV early in life. In a recent study, Nielsen-Saines et al. (2019) reinforce this conclusion. They observed that the neurologic phenotype in some ZIKV-exposed children may change from abnormal to normal from birth into early childhood, and vice versa (55).

Our SR has some limitations. Since ZIKV is an emerging disease, and despite the increasing number of SRs, one limitation is the lack of SRs on ZIKV in the literature. Because the Brazilian outbreak prompted much of the recent research, 7 of 21 (33%) included SRs were conducted fairly early in the epidemic between 2016 and 2017, 43% in 2018 and 24% in 2019, which can explain the lack of information on severe outcomes related to ZIKV infection or the presence of specific outcomes, caused by the inability to observe outcomes that are only evident or possible to detect in older children. Often the reported data are unclear as to the nature of the infection, i.e. whether included subjects are suspected ZIKV cases or confirmed ZIKV cases. Further, some of the included SRs did not report denominators, making interpretation difficult.

For peer review only

The low quality of the included SRs may indicate an important publication bias related to rare (e.g., ITP) or poorly reported outcomes (e.g., sleep disorders, epilepsy and auditory disorder) as these may not be captured in the search strategy.

Our study was strengthened by using a broad search strategy, without restrictions by language or publication type, reducing selection bias. To our knowledge, this is the first SR of SRs about health outcomes associated with ZIKV infection in humans.

As SRs of SRs aim to provide a summary of evidence from other SRs, although we were not able to perform a meta-analysis, our SR synthesizes findings from SRs on health outcomes associated with ZIKV infection in humans.

The evolving nature of the literature on ZIKV-associated health outcomes together with the critically low quality of existing SRs, confirm the need for high-quality SRs to better understand the burden of ZIKV, guide patient care and inform health policy.

Conclusion

Our SR demonstrates the need for future SRs on health outcomes associated with ZIKV infection as more research is published. As the ZIKV epidemic continues to evolve and the time since the emergence of the Brazilian outbreak increases we expect more primary observational studies on associated short- and long-term health outcomes to be published and synthesized in future SRs.

For peer review only

Acknowledgements

The authors would like to acknowledge Andrea Tricco and Joanna Bielecki for their guidance on this project.

Author Contributions

Raphael Ximenes: Conceptualization of the study, performed the systematic review, critically appraising the scientific literature, analysis, drafting and revising the manuscript.

Rafael N. Miranda: Performed the systematic review, critically appraising the scientific literature, revising the manuscript.

Lauren C. Ramsay: Performed the systematic review and critically appraising the scientific literature.

Shaun K. Morris: Critical revision of the manuscript.

Kellie E. Murphy: Critical revision of the manuscript.

RADAM-LAC Research Team: Contribution to study conception and design.

Beate Sander: Conceptualization of the study, critical revision of the manuscript, supervision of the study.

Funding

This project was funded by the Canadian Institutes of Health Research - Team grant - FRN149784 and the International Development Research Centre (IDRC).

Conflicts

The authors have no conflicts of interest to declare.

Data availability

All data underlining the results are available as part of the article and no additional source data are required.

For peer review only

References

1. World Health Organization. WHO | The History of Zika Virus. Who [Internet]. 2017 [cited 2018 Dec 10]; Available from: https://www.who.int/emergencies/zika-virus/timeline/en/

2. Posen HJ, Keystone JS, Gubbay JB, Morris SK. Epidemiology of Zika virus, 1947–2007. BMJ Glob Heal [Internet]. 2016 Aug [cited 2019 Feb 9];1(2):e000087. Available from: http://www.ncbi.nlm.nih.gov/pubmed/28588942

3. Duffy MR, Chen T-H, Hancock WT, Powers AM, Kool JL, Lanciotti RS, et al. Zika Virus Outbreak on Yap Island, Federated States of Micronesia. N Engl J Med [Internet]. 2009 Jun 11 [cited 2018 Dec 10];360(24):2536–43. Available from: http://www.nejm.org/doi/abs/10.1056/NEJMoa0805715

4. Kindhauser MK, Allen T, Frank V, Santhana RS, Dye C. Zika: the origin and spread of a mosquito-borne virus. Bull World Health Organ [Internet]. 2016 Sep 1;94(9):675–686C. Available from: http://www.who.int/entity/bulletin/volumes/94/9/16-171082.pdf

5. Pan American Health Organization / World Health Organization. Zika suspected and confirmed cases reported by countries and territories in the Americas Cumulative cases, 2015-2017. Updated as of 04 January 2018 [Internet]. Pan American Health Organization. Washington, D.C.; 2017 [cited 2019 Feb 9]. Available from: https://www.paho.org/hq/index.php?option=com_docman&view=download&category_slug=cumulative-cases-pdf-8865&alias=43296-zika-cumulative-cases-4-january-2018-296&Itemid=270&lang=en

6. World Health Organization. SITUATION REPORT ZIKA VIRUS MICROCEPHALY GUILLAIN-BARRÉ SYNDROME 10 MARCH 2017 DATA AS OF 9 MARCH 2017 [Internet]. [cited 2019 Feb 9]. Available from: https://apps.who.int/iris/bitstream/handle/10665/254714/zikasitrep10Mar17-eng.pdf?sequence=1

7. CDC. Zika Travel Information | Travelers' Health | CDC [Internet]. [cited 2019 May 28]. Available from: https://wwwnc.cdc.gov/travel/page/zika-travel-information

8. Dick GW. Zika virus (II). Pathogenicity and physical properties. Trans R Soc Trop Med Hyg [Internet]. 1952 Sep 1 [cited 2019 May 28];46(5):521–34. Available from: https://academic.oup.com/trstmh/article-lookup/doi/10.1016/0035-9203(52)90043-6

9. Cao-Lormeau VM, Blake A, Mons S, Lastere S, Roche C, Vanhomwegen J, et al. Guillain-Barre Syndrome outbreak associated with Zika virus infection in French Polynesia: a case-control study. Lancet. 2016;387:1531–9.

10. de Araújo TVB, Rodrigues LC, de Alencar Ximenes RA, de Barros Miranda-Filho D, Montarroyos UR, de Melo APL, et al. Association between Zika virus infection and microcephaly in Brazil, January to May, 2016: preliminary report of a case-control study. Lancet Infect Dis [Internet]. 2016 Dec 1 [cited 2019 Jan 28];16(12):1356–63. Available from: http://www.ncbi.nlm.nih.gov/pubmed/27641777

11. Saima Nasir JA. A Bibliometric Analysis of Research on Zika Virus Indexed in Web of Science. Adv Life Sci [Internet]. 2018 [cited 2018 Dec 4];5(3):88–95. Available from: http://www.als-journal.com/532-18/

For peer review only

12. Moher D, Shamseer L, Clarke M, Ghersi D, Liberatî A, Petticrew M, et al. Preferred reporting items for systematic review and meta-analysis protocols (PRISMA-P) 2015 statement [Internet]. 2015. Available from: http://www.crd.york.ac.uk/prospero

13. AMSTAR - Assessing the Methodological Quality of Systematic Reviews [Internet]. [cited 2018 Dec 4]. Available from: https://amstar.ca/Amstar-2.php

14. Shea BJ, Reeves BC, Wells G, Thuku M, Hamel C, Moran J, et al. AMSTAR 2: a critical appraisal tool for systematic reviews that include randomised or non-randomised studies of healthcare interventions, or both. BMJ [Internet]. 2017 Sep 21 [cited 2018 Dec 4];j4008. Available from: http://www.bmj.com/lookup/doi/10.1136/bmj.j4008

15. Krauer F, Riesen M, Reveiz L, Oladapo OT, Martínez-Vega R, Porgo T V., et al. Zika Virus Infection as a Cause of Congenital Brain Abnormalities and Guillain–Barré Syndrome: Systematic Review. von Seidlein L, editor. PLOS Med [Internet]. 2017 Jan 3;14(1):e1002203. Available from: https://dx.plos.org/10.1371/journal.pmed.1002203

16. Paixão ES, Barreto F, Teixeira M da G, Costa M da CN, Rodrigues LC. History, Epidemiology, and Clinical Manifestations of Zika: A Systematic Review. Am J Public Health [Internet]. 2016 Apr 9;106(4):606–12. Available from: http://ajph.aphapublications.org/doi/10.2105/AJPH.2016.303112

17. Chibueze EC, Tirado V, Lopes K da S, Balogun OO, Takemoto Y, Swa T, et al. Zika virus infection in pregnancy: a systematic review of disease course and complications. Reprod Health [Internet]. 2017 Dec 28;14(1):28. Available from: http://reproductive-health-journal.biomedcentral.com/articles/10.1186/s12978-017-0285-6

18. Coelho A, Crovella S, Coelho AVC, Crovella S. Microcephaly Prevalence in Infants Born to Zika Virus-Infected Women: A Systematic Review and Meta-Analysis. Int J Mol Sci [Internet]. 2017 Aug 5;18(8):1714. Available from: http://www.mdpi.com/1422-0067/18/8/1714

19. Simões R, Buzzini R, Bernardo W, Cardoso F, Salomão A, Cerri G, et al. Zika virus infection and pregnancy. Rev Assoc Med Bras [Internet]. 2016 Apr;62(2):108–15. Available from: http://www.scielo.br/scielo.php?script=sci_arttext&pid=S0104-42302016000200108&lng=en&tlng=en

20. Padilla C, Pan A, Geller A, Zakowski MI. Zika virus: review and obstetric anesthetic clinical considerations. J Clin Anesth [Internet]. 2016 Dec 1;35:136–44. Available from: https://www.sciencedirect.com/science/article/pii/S0952818016304299

21. Marques V de M, Santos CS, Santiago IG, Marques SM, Nunes Brasil M das G, Lima TT, et al. Neurological Complications of Congenital Zika Virus Infection. Pediatr Neurol [Internet]. 2019 Feb;91:3–10. Available from: http://www.ncbi.nlm.nih.gov/pubmed/30591235

22. Counotte MJ, Egli-Gany D, Riesen M, Abraha M, Porgo TV, Wang J, et al. Zika virus infection as a cause of congenital brain abnormalities and Guillain-Barré syndrome: From systematic review to living systematic review. PLoS Med [Internet]. 2018 Feb 15;7:196. Available from: http://www.ncbi.nlm.nih.gov/pubmed/30631437

23. Haby MM, Pinart M, Elias V, Reveiz L. Prevalence of asymptomatic Zika virus infection: a systematic review. Bull World Health Organ [Internet]. 2018 Jun 1;96(6):402–413D. Available

For peer review only

from: http://www.ncbi.nlm.nih.gov/pubmed/29904223

24. Rehan Sarwar M, Saqib A, Iftikhar S. Zika Virus Infection during Pregnancy; Maternofetal Risk Assessment, Transmission, Complications, and Management: A Review of the Literature. Arch Clin Infect Dis [Internet]. 2018 Jun 24;13(3). Available from: http://archcid.com/en/articles/12848.html

25. Wahid B, Ali A, Waqar M, Idrees M. An updated systematic review of Zika virus-linked complications. Asian Pac J Trop Med [Internet]. 2018;11(1):1. Available from: http://www.apjtm.org/text.asp?2018/11/1/1/223527

26. Soriano-Arandes A, Rivero-Calle I, Nastouli E, Espiau M, Frick M, Alarcon A, et al. What we know and what we don't know about perinatal Zika virus infection: a systematic review. Expert Rev Anti Infect Ther [Internet]. 2018 Mar 4;16(3):243–54. Available from: http://www.ncbi.nlm.nih.gov/pubmed/29415586

27. Barbi L, Coelho AVC, Alencar LCA de, Crovella S. Prevalence of Guillain-Barré syndrome among Zika virus infected cases: a systematic review and meta-analysis. Brazilian J Infect Dis [Internet]. 2018 Mar;22(2):137–41. Available from: http://www.ncbi.nlm.nih.gov/pubmed/29545017

28. Santos GRB dos, Aragão FBA, Lobão WJ de M, Lima FR, Andrade LMRL de, Furtado QR, et al. Relationship between microcephaly and Zika virus during pregnancy: a review. Rev Assoc Med Bras [Internet]. 2018 Jul;64(7):635–42. Available from: http://www.ncbi.nlm.nih.gov/pubmed/30365666

29. Wachira VK, Peixoto HM, Fernandes De Oliveira MR. Systematic review of factors associated with the development of Guillain-Barr e syndrome 2007-2017: what has changed? 2018; Available from: https://v2dis-prod.evidencepartners.com/Generic/getAttachment2.php?id=44

30. Pomar L, Musso D, Malinger G, Vouga M, Panchaud A, Baud D. Zika virus during pregnancy: From maternal exposure to congenital Zika virus syndrome. Prenat Diagn [Internet]. 2019 May [cited 2019 Aug 1];39(6):420–30. Available from: http://www.ncbi.nlm.nih.gov/pubmed/30866073

31. Wilder-Smith A, Chang CR, Leong WY. Zika in travellers 1947-2017: a systematic review. J Travel Med [Internet]. 2018 [cited 2019 Aug 1];25(1). Available from: http://www.ncbi.nlm.nih.gov/pubmed/30016469

32. Nithiyanantham SF, Badawi A. Maternal infection with Zika virus and prevalence of congenital disorders in infants: systematic review and meta-analysis. Can J Public Heal [Internet]. 2019 May 10 [cited 2019 Aug 1]; Available from: http://www.ncbi.nlm.nih.gov/pubmed/31077071

33. Masel J, McCracken MK, Gleeson T, Morrison B, Rutherford G, Imrie A, et al. Does prior dengue virus exposure worsen clinical outcomes of Zika virus infection? A systematic review, pooled analysis and lessons learned. Diemert DJ, editor. PLoS Negl Trop Dis [Internet]. 2019 Jan 25 [cited 2019 Aug 1];13(1):e0007060. Available from: http://www.ncbi.nlm.nih.gov/pubmed/30682026

34. Barbosa MH de M, Magalhães-Barbosa MC de, Robaina JR, Prata-Barbosa A, Lima MA de MT de, Cunha AJLA da. Auditory findings associated with Zika virus infection: an integrative review. Braz J Otorhinolaryngol [Internet]. 2019 Jun 18 [cited 2019 Aug 1]; Available from: http://www.ncbi.nlm.nih.gov/pubmed/31296482

For peer review only

35. Minhas AM, Nayab A, Iyer S, Narmeen M, Fatima K, Khan MS, et al. Association of Zika Virus with Myocarditis, Heart Failure, and Arrhythmias: A Literature Review. Cureus [Internet]. 2017 Jun 27 [cited 2019 Aug 1];9(6):e1399. Available from: http://www.ncbi.nlm.nih.gov/pubmed/28856072

36. Brasil P, Pereira JP, Moreira ME, Ribeiro Nogueira RM, Damasceno L, Wakimoto M, et al. Zika Virus Infection in Pregnant Women in Rio de Janeiro. N Engl J Med [Internet]. 2016 Dec 15 [cited 2019 Aug 6];375(24):2321–34. Available from: http://www.ncbi.nlm.nih.gov/pubmed/26943629

37. Mlakar J, Korva M, Tul N, Popović M, Poljšak-Prijatelj M, Mraz J, et al. Zika Virus Associated with Microcephaly. N Engl J Med [Internet]. 2016 Mar 10 [cited 2019 Aug 6];374(10):951–8. Available from: http://www.ncbi.nlm.nih.gov/pubmed/26862926

38. de Paula Freitas B, de Oliveira Dias JR, Prazeres J, Sacramento GA, Ko AI, Maia M, et al. Ocular Findings in Infants With Microcephaly Associated With Presumed Zika Virus Congenital Infection in Salvador, Brazil. JAMA Ophthalmol [Internet]. 2016 May 1 [cited 2019 Aug 6];134(5):529. Available from: http://www.ncbi.nlm.nih.gov/pubmed/26865554

39. Schuler-Faccini L, Ribeiro EM, Feitosa IML, Horovitz DDG, Cavalcanti DP, Pessoa A, et al. Possible Association Between Zika Virus Infection and Microcephaly — Brazil, 2015. MMWR Morb Mortal Wkly Rep [Internet]. 2016 Jan 29 [cited 2019 Aug 6];65(3):59–62. Available from: http://www.ncbi.nlm.nih.gov/pubmed/26820244

40. Cao-Lormeau V-M, Blake A, Mons S, Lastère S, Roche C, Vanhomwegen J, et al. Guillain-Barré Syndrome outbreak associated with Zika virus infection in French Polynesia: a case-control study. Lancet [Internet]. 2016 Apr 9 [cited 2018 Dec 4];387(10027):1531–9. Available from: https://www.sciencedirect.com/science/article/pii/S0140673616005626

41. Honein MA, Dawson AL, Petersen EE, Jones AM, Lee EH, Yazdy MM, et al. Birth Defects Among Fetuses and Infants of US Women With Evidence of Possible Zika Virus Infection During Pregnancy. JAMA [Internet]. 2017 Jan 3 [cited 2019 Aug 6];317(1):59. Available from: http://jama.jamanetwork.com/article.aspx?doi=10.1001/jama.2016.19006

42. Meaney-Delman D, Hills SL, Williams C, Galang RR, Iyengar P, Hennenfent AK, et al. Zika Virus Infection Among U.S. Pregnant Travelers — August 2015–February 2016. MMWR Morb Mortal Wkly Rep [Internet]. 2016 Mar 4 [cited 2019 Aug 6];65(8):211–4. Available from: http://www.ncbi.nlm.nih.gov/pubmed/26938703

43. Cauchemez S, Besnard M, Bompard P, Dub T, Guillemette-Artur P, Eyrolle-Guignot D, et al. Association between Zika virus and microcephaly in French Polynesia, 2013–15: a retrospective study. Lancet [Internet]. 2016 May 21 [cited 2019 Aug 6];387(10033):2125–32. Available from: http://www.ncbi.nlm.nih.gov/pubmed/26993883

44. Oliveira Melo AS, Malinger G, Ximenes R, Szejnfeld PO, Alves Sampaio S, Bispo de Filippis AM. Zika virus intrauterine infection causes fetal brain abnormality and microcephaly: tip of the iceberg? Ultrasound Obstet Gynecol [Internet]. 2016 Jan 1 [cited 2019 Aug 6];47(1):6–7. Available from: http://doi.wiley.com/10.1002/uog.15831

45. Besnard M, Eyrolle-Guignot D, Guillemette-Artur P, Lastère S, Bost-Bezeaud F, Marcelis L, et al. Congenital cerebral malformations and dysfunction in fetuses and newborns following the 2013 to 2014 Zika virus epidemic in French Polynesia. Eurosurveillance [Internet]. 2016 Mar 31 [cited

For peer review only

2019 Aug 6];21(13):30181. Available from: http://www.ncbi.nlm.nih.gov/pubmed/27063794

46. van der Linden V, Pessoa A, Dobyns W, Barkovich AJ, Júnior H van der L, Filho ELR, et al. Description of 13 Infants Born During October 2015–January 2016 With Congenital Zika Virus Infection Without Microcephaly at Birth — Brazil. MMWR Morb Mortal Wkly Rep [Internet]. 2016 Dec 2 [cited 2019 Aug 6];65(47):1343–8. Available from: http://www.cdc.gov/mmwr/volumes/65/wr/mm6547e2.htm

47. Soares de Oliveira-Szejnfeld P, Levine D, Melo AS de O, Amorim MMR, Batista AGM, Chimelli L, et al. Congenital Brain Abnormalities and Zika Virus: What the Radiologist Can Expect to See Prenatally and Postnatally. Radiology [Internet]. 2016 Oct [cited 2019 Aug 6];281(1):203–18. Available from: http://www.ncbi.nlm.nih.gov/pubmed/27552432

48. Calvet G, Aguiar RS, Melo ASO, Sampaio SA, de Filippis I, Fabri A, et al. Detection and sequencing of Zika virus from amniotic fluid of fetuses with microcephaly in Brazil: a case study. Lancet Infect Dis [Internet]. 2016 Jun [cited 2019 Aug 6];16(6):653–60. Available from: http://www.ncbi.nlm.nih.gov/pubmed/26897108

49. França GVA, Schuler-Faccini L, Oliveira WK, Henriques CMP, Carmo EH, Pedi VD, et al. Congenital Zika virus syndrome in Brazil: a case series of the first 1501 livebirths with complete investigation. Lancet [Internet]. 2016 Aug 27 [cited 2019 Aug 6];388(10047):891–7. Available from: http://www.ncbi.nlm.nih.gov/pubmed/27372398

50. Besnard M, Lastère S, Teissier A, Cao-Lormeau V, Musso D. Evidence of perinatal transmission of Zika virus, French Polynesia, December 2013 and February 2014. Eurosurveillance [Internet]. 2014 Apr 3 [cited 2019 Aug 6];19(13):20751. Available from: http://www.eurosurveillance.org/ViewArticle.aspx?ArticleId=20751

51. Kleber de Oliveira W, Cortez-Escalante J, De Oliveira WTGH, do Carmo GMI, Henriques CMP, Coelho GE, et al. Increase in Reported Prevalence of Microcephaly in Infants Born to Women Living in Areas with Confirmed Zika Virus Transmission During the First Trimester of Pregnancy — Brazil, 2015. MMWR Morb Mortal Wkly Rep [Internet]. 2016 Mar 11 [cited 2019 Aug 6];65(9):242–7. Available from: http://www.ncbi.nlm.nih.gov/pubmed/26963593

52. Oehler E, Watrin L, Larre P, Leparc-Goffart I, Lastere S, Valour F, et al. Zika virus infection complicated by Guillain-Barre syndrome--case report, French Polynesia, December 2013. Euro Surveill [Internet]. 2014 Mar 6 [cited 2019 Aug 16];19(9). Available from: http://www.ncbi.nlm.nih.gov/pubmed/24626205

53. Anthony Boadle LP. Exclusive: Brazil says Zika virus outbreak worse than believed | Reuters [Internet]. Reuters. 2016 [cited 2018 Dec 4]. Available from: https://www.reuters.com/article/us-health-zika-brazil-exclusive-idUSKCN0VA331

54. World Health Organization. ZIKA SITUATION REPORT - ZIKA AND POTENTIAL COMPLICATIONS 12 FEBRUARY 2016 [Internet]. 2016 [cited 2018 Dec 4]. Available from: https://www.who.int/emergencies/zika-virus/situation-report/who-zika-situation-report-12-02-2016.pdf

55. Nielsen-Saines K, Brasil P, Kerin T, Vasconcelos Z, Gabaglia CR, Damasceno L, et al. Delayed childhood neurodevelopment and neurosensory alterations in the second year of life in a

For peer review only

prospective cohort of ZIKV-exposed children. Nat Med [Internet]. 2019 Aug 8 [cited 2019 Aug 14];25(8):1213–7. Available from: http://www.nature.com/articles/s41591-019-0496-1

For peer review only

Table 1. Summary of included systematic reviews

Author, Year	Aim	Search period	Number of studies included	Types of studies included in review	Jurisdictions of included studies (n studies)
Krauer et al. (2017) (15)	To assess the relationship between ZIKV infection and congenital brain abnormalities and Guillain-Barré syndrome	From inception until May 30, 2016	106	Case reports, case series, case-control studies, cohort studies, cross-sectional studies, ecological study/outbreak reports, modelling studies, animal experiments, in vitro experiments, sequence analysis and phylogenetics	Brazil (6), Cabo Verde (2), Colombia (1), French Polynesia (2), Martinique (2), Panama (5), El Salvador (1), Haiti (119), Puerto Rico (1), Venezuela (1), Slovenia*, Netherlands*, Dominican Republic*, French Guiana*, Honduras*, Paraguay*, Suriname*, Micronesia*, Pacific Islands*<br>* Not possible to know number of studies from these countries
Paixão et al. (2016) (16)	To summarize current knowledge on ZIKV including epidemiology, clinical presentation, and complications	1954 to Jan 2016	41	Case reports, case series, surveillance reports, cross-sectional studies, epidemiological bulletins and alerts	Not clearly reported.<br>Most data are from Brazil and French Polynesia.
Chibueze et al. (2017) (17)	To summarize guidance on pregnancy care in the context of ZIKV infection	From inception until March 3, 2016	18	Case reports, case series, observational studies	Brazil (11)<br>Colombia (1)<br>France (1)<br>Puerto Rico (1)<br>Slovenia (1)<br>USA (2)<br>Venezuela (1)
Coelho et al. (2017) (18)	To summarize evidence and meta-analyze data to estimate prevalence	Not reported	8	Cohort studies	Brazil (1)<br>Colombia (1)<br>French Guiana (1)<br>Puerto Rico (1)

For peer review only

Author, Year	Aim	Search period	Number of studies included	Types of studies included in review	Jurisdictions of included studies (n studies)
	of microcephaly in babies born to ZIKV infected pregnant women				USA (4)
Simões et al. (2016) (19)	To assess the effects of Zika virus infection on during pregnancy and postpartum periods	From inception until Feb 23, 2016	30	Case reports, case series, guidelines	Not clearly reported; most data are from Brazil.
Padilla et al. (2016) (20)	To review clinical and basic science literature about ZIKV infection relevant for obstetric anesthesiologists	From inception until Apr 15, 2016	30	Systematic reviews, narrative reviews, case reports, epidemiologic studies, government reports, and news articles	Not clearly reported.
Marques et al. (2019) (21)	To map the neurological damage and outcomes related to congenital ZIKV infection	Jan 1966 to Aug 2018	28	Not informed	Brazil (16)<br>USA (3)<br>Colombia (1)
Counotte et al. (2018) (22)	To summarize the evidence of the casual associations between ZIKV and CZS and GBS	May 30, 2016 to Jan 18, 2017	101	Case report, case series, case-control study, cohort study, cross-sectional study, controlled trials, ecological study/outbreak report, modelling study, animal experiment, in vitro experiment, sequencing and phylogenetics, biochemical/protein structure studies	USA, Martinique, Brazil, Suriname, Colombia, French Guiana, Slovenia, Spain, Uganda, Nicaragua, Barbados, Belize, Dominican Republic, El Salvador, Guatemala, Haiti, Honduras; Mexico, Republic of Marshall Islands, Venezuela, French Polynesia, Ecuador, France, Puerto Rico, Guadeloupe,

For peer review only

Author, Year	Aim	Search period	Number of studies included	Types of studies included in review	Jurisdictions of included studies (n studies)
					Guyana, New Zealand, French Southern Territories
Haby et al., (2018) (23)	To estimate and meta-analyze the prevalence of asymptomatic Zika virus infection in the general population and in specific population groups from observational epidemiological studies	From inception until Jan 26, 2018	23	Cross-sectional seroprevalence studies, case series, case-control, cohort	USA (6), Brazil (3), French Polynesia (3), French Guiana (3), Puerto Rico (2), Colombia (2), Spain (2), Micronesia (1), Martinique (1)
Sarwar et al. (2018) (24)	To report on the current literature regarding ZIKV and its hazardous effects on maternofetal health with a special emphasis on risk assessment, virus transmission, associated complications, and possible management	2007 to May 2017	69	Not informed	Argentina, Bolivia, Brazil, Colombia, French Guiana, Suriname, Paraguay, Trinidad and Tobago, Canada, Dominican Republic, Grenada, Guadeloupe, Guatemala, Haiti, Martinique, Puerto Rico, USA, Costa Rica, El Salvador, Honduras, Nicaragua, Panama, Europe, Slovenia, Spain, Thailand, Vietnam, French Polynesia, Marshall Islands, Cape Verde
Wahid et al. (2018) (25)	To summarize the evidence of neurological complications in ZIKV-infected people	2015 to March 2017	35	Case-studies, case-cohort studies, cross-sectional studies, organizational survey reports and case-control studies	Brazil (15)<br>French Polynesia (4)<br>Colombia (3)

For peer review only

Author, Year	Aim	Search period	Number of studies included	Types of studies included in review	Jurisdictions of included studies (n studies)
					USA, Slovenia, Suriname, Spain, Haiti, Martinique, Netherlands, Ecuador, Guyana (1)
Soriano-Arandes et al. (2018) (26)	To summarize the new evidence in aspects of epidemiology, virology, pathogenesis, associated risk factors during pregnancy, newborn phenotypic signs, neuroimaging, laboratory diagnosis, treatment and vaccines	From inception until Nov 30, 2017	106	Case series, cohort (prospective/retrospective), cross-sectional or case-control studies	Brazil, French Polynesia, USA, Martinique, Colombia
Barbi et al. (2018) (27)	To systematically review the literature and perform a meta-analysis to estimate the prevalence of GBS among ZIKV-infected individuals	From inception until Nov 2017	3	Case series, epidemiological surveys, cross-sectional or cohort studies	French Polynesia (1), Suriname and Dominican Republic (1), South American, Central American and Caribbean countries (1)
Santos et al. (2018) (28)	To analyze the association between Zika-virus and microcephaly during the gestational period	From inception until Dec 2016	35	Not informed	Brazil
Wachira et al. (2018) (29)	To describe the factors associated with development of GBS, both infectious and	Jan 1, 2007 to Jun 30, 2017	34	The most common were case control, cohort, self-controlled case series	French Polynesia

For peer review only

Author, Year	Aim	Search period	Number of studies included	Types of studies included in review	Jurisdictions of included studies (n studies)
	non-infectious, through a SR.				
Pomar et al. (2019) (30)	Present a review to describe the risks and complications of maternal and subsequent fetal infection by ZIKV.	Jun 2009 to Nov 2018	68	Not informed	Colombia (3), Puerto Rico (1), French Guiana (3), Brazil (1), Yap Island (1), USA (2)
Wilder-Smith et al. (2018) (31)	Describe the burden of ZIKV infection in international travelers over time; estimate the proportion of birth defects as a result of maternal ZIKV infection in travelers; track the extent of sexual transmission; summarize data on ZIKV cases in travelers identifying counties with reports on local transmission	1947 to Apr 2017	65	Surveillance reports, case reports, retrospective (multi-centre study), descriptive retrospective analysis and prospective cohort study	USA (9), Canada (2), Germany (3), Norway (1), France (5), Italy (7), Japan (2), Australia (4), New Caledonia (1), Finland (1), Mexico (1), Slovenia (1), Netherlands (4), Belgium (1), Portugal (1), Switzerland (3), Israel (1), Taiwan (2), Spain (1), China (7), South Korea (2), UK (2), Singapore (1), Malaysia (1)
Nithiyanantham et al. (2019) (32)	To conduct a systematic review and meta-analysis on the prevalence of congenital Zika-related disorders in infants of mothers	From inception until Oct 31, 2017	25	Case series, epidemiological reports, prospective and retrospective studies, cohort studies and cross-sectional studies	USA (8), Brazil (6), Colombia (2), Puerto Rico (1), French Polynesia (1), Martinique (1), Trinidad and Tobago (1), French Guiana (1), Ecuador (1), Spain (1)

For peer review only

Author, Year	Aim	Search period	Number of studies included	Types of studies included in review	Jurisdictions of included studies (n studies)
	infected with ZIKV during pregnancy.				
Masel et al. (2019) (33)	To determine if prior infection with DENV, as compared with those with no prior DENV infection, is associated with a greater risk of ZIKV complications (including neurological and teratogenic outcomes), greater ZIKV peak viremia, greater area-under-the-curve of viremia or other putative laboratory proxies of ZIKV severity.	From inception until Mar 25, 2018	5	Case control study	Brazil (2), French Polynesia (5)
Barbosa et al. (2019) (34)	To describe the auditory alterations, pathogenesis and recommendations for follow-up in individuals with prenatal or acquired ZIKV infection.	From inception until Apr 2019	27	Case report and case series	Brazil (14), Colombia (3), USA (2), French Polynesia (1), Puerto Rico (1)
Minhas et al. (2017) (35)	Focuses on the potential threat that ZIKV may pose to the heart like that of	From inception until March 2017	3	Case report and prospective observational multicenter study	France (1), Venezuela (1), China (1)

For peer review only

Author, Year	Aim	Search period	Number of studies included	Types of studies included in review	Jurisdictions of included studies (n studies)
	similar arboviral diseases.				

For peer review only

Table 2. Overlap between systematic reviews

Number of citations	Title	Author	Cited by
10	Zika virus infection in pregnant women in Rio de Janeiro	Brasil et al. (2016) (36)	(15,17,18,20–22,25,26,30,32)
8	Zika virus associated with microcephaly	Mlakar et al. (2016) (37)	(15,17,19,24–26,28,31)
7	Ocular findings in infants with microcephaly associated with presumed Zika virus congenital infection in Salvador, Brazil	de Paula Freitas et al. (2016) (38)	(15,17,19–21,25,30)
7	Possible association between Zika virus infection and microcephaly - Brazil, 2015	Schuler-Faccini et al. (2016) (39)	(15,17,21,25,26,28,30)
6	Guillain-Barre Syndrome outbreak associated with Zika virus infection in French Polynesia: a case-control study	Cao-Lormeau et al. (2016) (40)	(15,20,23–25,33)
6	Birth defects among fetuses and infants of US women with evidence of possible Zika virus infection during pregnancy	Honein et al. (2017) (41)	(18,21,22,24,26,32)
6	Zika virus infection among U.S. pregnant travelers - August 2015 - February 2016	Meaney-Delman et al. (2016) (42)	(15,17,18,20,31,32)
6	Zika virus outbreak on Yap Island, Federated States of Micronesia	Duffy et al. (2009) (3)	(15,16,19,23,24,30)
6	Association between Zika virus and microcephaly in French Polynesia, 2013 - 15: a retrospective study	Cauchemez et al. (2016) (43)	(15,17,24–26,30)
6	Zika virus intrauterine infection causes fetal brain abnormality and microcephaly: tip of the iceberg?	Oliveira et al. (2016) (44)	(15,17,20,21,26,28)
5	Congenital cerebral malformations and dysfunction in fetuses and newborns following the 2013 to 2014 Zika virus epidemic in French Polynesia	Besnard et al. (2016) (45)	(15,25,30,32,34)
5	Description of 13 infants born during October 2015 - January 2016 with congenital Zika virus infection without microcephaly at birth - Brazil	van der Linden et al. (2016) (46)	(21,22,26,30,34)
5	Congenital brain abnormalities and Zika virus: what the radiologist can expect to see prenatally and postnatally	Oliveira-Szejnfeld et al. (2016) (47)	(21,22,26,30,32)
5	Detection and sequencing of Zika virus from amniotic fluid of fetuses with microcephaly in Brazil: a case study	Calvet et al. (2016) (48)	(15,17,19,28,30)

For peer review only - http://bmjopen.bmj.com/site/about/guidelines.xhtml

5	Congenital Zika virus syndrome in Brazil: a case series of the first 1501 livebirths with complete investigation	França et al. (2016) (49)	(21,22,25,26,30)
5	Evidence of perinatal transmission of Zika virus, French Polynesia, December 2013 and February 2014	Besnard et al. (2014) (50)	(16,17,24,26,28)
5	Increase in reported prevalence of microcephaly in infants born to women living in areas with confirmed Zika virus transmission during the first trimester of pregnancy - Brazil, 2015	Oliveira et al. (2016) (51)	(15,17,20,24,25)
5	Zika virus infection complicated by Guillain-Barre syndrome - case report, French Polynesia, December 2013	Oehler et al. (2014) (52)	(15,16,19,20,25)

Figure legends

Figure 1: PRISMA flow diagram of search results and study selection.

Figure 2: Overlap between studies cited in at least 5 systematic reviews.

Figure 3: Individual study results of quality assessment using AMSTAR 2 - Result for all questions of AMSTAR 2 tool.

Figure 4: Individual study results of quality assessment using AMSTAR 2 - Critical domains of AMSTAR 2 tool.

For peer review only

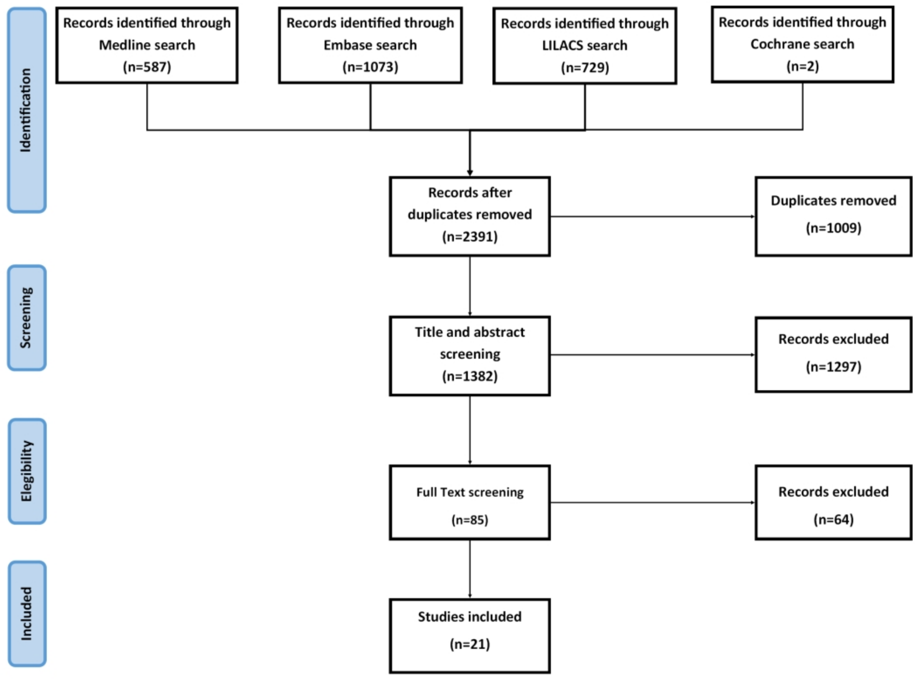

PRISMA flow diagram of search results and study selection.

279x215mm (300 x 300 DPI)

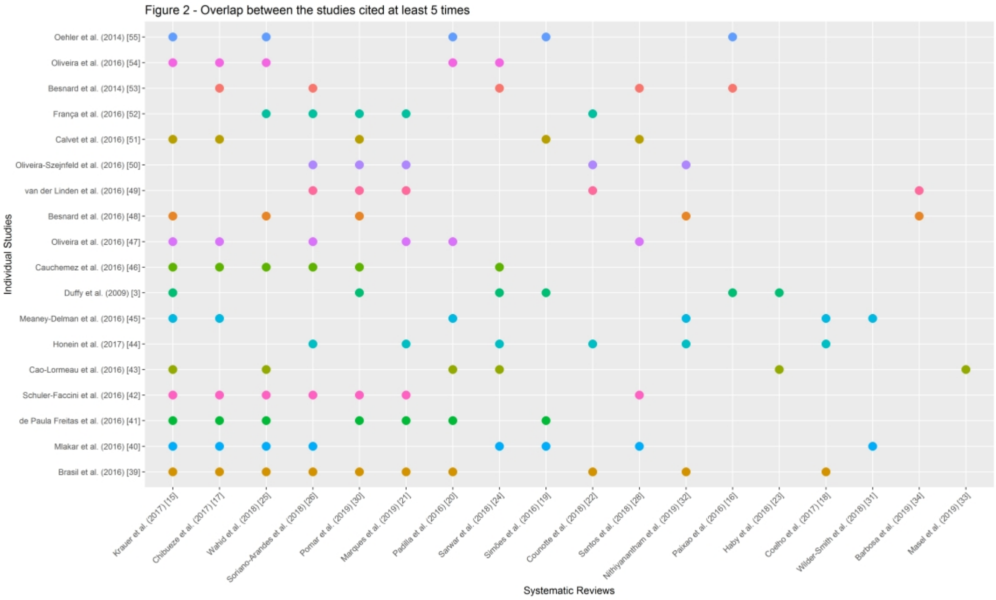

Overlap between studies cited in at least 5 systematic reviews.

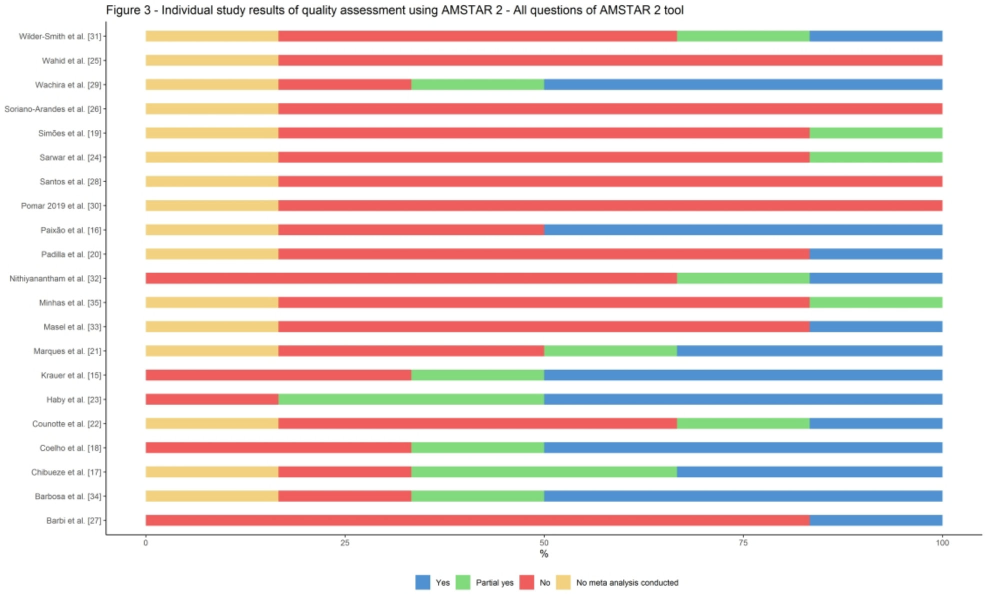

Individual study results of quality assessment using AMSTAR 2 - Result for all questions of AMSTAR 2 tool.

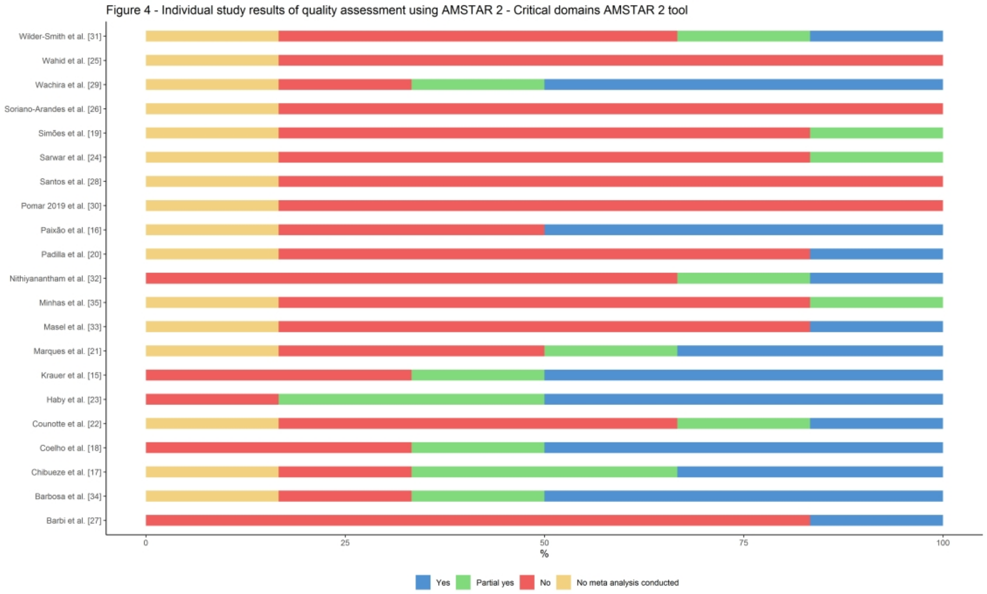

Individual study results of quality assessment using AMSTAR 2 - Critical domains of AMSTAR 2 tool.

Search Strategy

Database: Embase Classic+Embase <1947 to 2018 February 27>

Search Strategy:

1 zika fever/ or zika virus/ or zika virus vaccine/ or zika.mp. (5013)

2 "systematic review"/ or "review"/ (2367967)

3 1 and 2 (569)

Database: Ovid MEDLINE(R) <1946 to February Week 3 2018>

Search Strategy:

1 exp ZIKA VIRUS INFECTION/ or exp ZIKA VIRUS/ or zika.mp. (2287)

2 "review"/ (2215441)

3 1 and 2 (326)

Database: Cochrane

Search Strategy:

1 ZIKA and review (2)

Update – 22/07/2019

Database: LILACS

Search Strategy:

(tw:((tw:(ZIKA VIRUS INFECTION)) OR (tw:(ZIKA VIRUS)) OR (tw:(zika.mp)))) AND (tw:(systematic review)) (729)

Database(s): Ovid MEDLINE(R) 1946 to July Week 2 2019

Search Strategy:

1 exp ZIKA VIRUS INFECTION/ or exp ZIKA VIRUS/ or zika.mp. (4560)

2 "review"/ (2360456)

3 1 and 2 (722)

4 limit 3 to yr="2018-Current" (261)

Database(s): Embase Classic+Embase <1947 to 2019 July 19>

Search Strategy:

1 zika fever/ or zika virus/ or zika virus vaccine/ or zika.mp. (8593)

2 "systematic review"/ or "review"/ (2553024)

3 1 and 2 (1056)

4 limit 3 to yr="2018-Current" (504)

For peer review only

Database: Cochrane

Search Strategy:

1 ZIKA and review (0)

For peer review only

Supplementary file 2 - Table 1. Health outcomes - Congenital Zika syndrome

Authors	Malformations / Congenital abnormalities	Brain abnormalities	Microcephaly	Ocular disorders	Auditory disorder - Rate	Cardiovascular damage
Krauer et al. (2017) [15]		Prevalence: 96% of cases	91% of cases; Prevalence ratio over states with no reported cases of microcephaly= 4.67	Prevalence: 42% (49 cases in 116 mother-infant pairs)	13% (3 cases in 24 mother-infant pairs)	
Paixao et al. (2016) [16]		In 2015, the prevalence of microcephaly in Brazil was 20 cases per 10,000 live births; Zika infection during 9 pregnancies confirmed by CDC resulted in the birth of a neonate with microcephaly.	Rate per 100,000 live births: 121.7 (0.12%) in 2015 in Brazil; Death due microcephaly: 1.3% in suspected microcephaly cases			
Chibueze et al. (2017) [17]		In one observational study of 35 infants with microcephaly, 11 fetuses had intra-uterine brain injury accompanied by stunting of cerebral growth prior to birth.	One observational study provided a trimester-specific modelling estimate risk for microcephaly per 10,000 ZIKV infected pregnant women per trimester of pregnancy: 1st 95 (34 - 191), 2nd 84 (12 - 196), 3rd trimester: 0 - (0 - 251)			
Coelho et al. (2017) [18]	Other organs damage: French Guiana 2% in 250 live births or mother-infant pair. USA: 7%. Not clear if the denominator is the number of live births or mother-infant pair (301 or 498 respectively)		0.3% in live-birth pregnancies; 14.3% - in live-birth pregnancies; Prevalence (cases/all pregnancies): 2.3%. Prevalence (cases/live births): 2.7%. Death due to microcephaly: 8.3%, would be 5.7% in case of new confirmed cases are included.	Two studies reported a prevalence of ocular damage (0.9% and 1%). It is not clear if the denominator is the number of live births (395 and 301, respectively) or the number mother-infant pair (442 and 498, respectively)		French Guiana: Cardiovascular damage equal to 1%. The denominator is unclear if is the number of live births or mother-infant pair (301 and 498 respectively)
Simoes et al. (2016) [19]	Prevalence of CZS: 10 to 20 cases in 100,000 live births; 8.87% of cases with confirmed changes in CNS		The Ministry of Health in Brazil reported an increase in the number of cases of microcephaly close to 20 times that previously reported (aproximately 0.5 cases for each 10,000 live births) which means 10 microcephaly cases per 10,000 births.			
Padilla et al. (2016) [20]	In 72 women with Zika-positive serology during pregnancy in Brazil, 29% had abnormalities detected on fetal ultrasound. Central nervous system abnormalities were noted after Zika infections as late as 27 weeks' gestation, and placental insufficiency was noted with even later gestational ages.		In 2015, the prevalence of microcephaly in Brazil was 20 cases per 10,000 live births; Zika infection during 9 pregnancies confirmed by CDC resulted in the birth of a neonate with microcephaly.			
Marques et al. (2019) [21]		% of neurological malformations: Subcortical-cortical junction calcifications: 92.9%, Basal ganglia calcifications: 57.1%, Periventricular calcifications: 29.5%. Ventriculomegaly/hydrocephaly: 63.1%. Cerebellar abnormalities: 46.2%, 82% (14 of 17 patients). Corpus callosum abnormalities: 47.9%	39.7% in cases of congenital Zika infection. Almost 100% when the infection occurred during the first trimester and decreased when the infection occurred in the second or third trimester	Prevalence: 44.3% in congenital ZIKV infection, 20% in patients with microcephaly, 33% in patients with ventriculomegaly, and 43% in patients with calcification. Bilateral findings: 76.8% of infants with ocular lesions. In eyes of infants with ocular lesions and congenital ZIKV infection: Macular lesions in 50%, Optical nerve abnormalities: 27.78%, Chorioretinal artrophy/scaring: 10.65%, Focal pigment mottling of retina: 6.94%, Microphtalmia: 3.70%, Glaucoma: 2.31%, Cataract: 2.31%, Iris coloboma: 2.31%, Subluxation: 1.39%		
Counotte et al. (2018) [22]	Prevalence of adverse congenital outcomes: 8.97-49.57% in ZIKV positive women. Birth defects: 5.9% in pregnant asymptomatic women and 5.98% in symptomatic pregnant women		RR between ZIKV exposed and unexposed: 4.4-6.6. OR between women with confirmed ZIKV and without evidence of ZIKV infection: 11.0-55.5			
Haby et al. (2018) [23]			Prevalence of asymptomatic ZIKV infection in mothers who gave birth to babies with microcephaly: 0.36			

Authors	Malformations / Congenital abnormalities	Brain abnormalities	Microcephaly	Ocular disorders	Auditory disorder - Rate	Cardiovascular damage
Sarwar et al. (2018) [24]		Prevalence in dead neonates of ZIKV infected mothers: Pachygyria: 14.28%, Arthrogryposis: 14.28%. Morphologic microcephalus changes: 14.28%. Ventriculomegaly / hydrocephaly: 100%. Cerebellar abnormalities: 28.57%	Risk of 1% when infection occurred in the first trimester of pregnancy	In ZIKV infected infants: Retinal impairment: 28%, Impaired optic nerve: 17%, Optic nerve hypoplasia: 8%		
Wahid et al. (2018) [25]	Fetal abnormalities 28.57% in infected pregnant women. Ventricular calcifications or other central nervous system abnormal amniotic fluid volume or cerebral or umbilical artery flow: 16.67%. (CNS) lesions: 16.67%. 80 of the 185 infants, ZIKV-linked microcephaly: 10 (the value of the denominator is not clear) neonates, 5 of 80 or 185 birth defects such as hydranencephaly, holoprosencephaly, clubfeet, and craniosynostosis, 3 of 80 or 185: cataracts, holoprosencephaly, and ventral pons hypoplasia	Prevalence: 28% (including microcephaly) in newborns of mothers infected with ZIKV	Risk of microcephaly: 0-30%. Relative Risk 100–1,000 (assuming 10% exposure) or 20–200 (assuming 50% exposure) compared to background risk of microcephaly. Prevalence: 50.47% among definite or probable ZIKV cases. Higher risk of microcephaly in pregnant women infected during first trimester. Estimated risk of microcephaly: 0.95% in women infected in the first trimester	In infants with microcephaly: ophthalmoscopic alterations in 50% (not clear if ZIKV-related infection) . Ocular findings 34.5-58.62% of ZIKV linked microcephalic infants		
Soriano-Arandes et al. (2018) [26]	Birth defects: 6% in asymptomatic and symptomatic pregnant women. From 1 study: Fetal adverse outcomes in women infected with ZIKV: 55% in the first term of pregnancy, 29% in the third trimester. In infants with CZS: Dimples: 30.1%, Distal hand/finger contractures: 20.5%, feet malposition: 15.7%, generalized arthrogryposis: 9.6%, birth defects in women with recent ZIKV infection: 6%	Prevalence: Microcephaly in 86.7% and craniofacial disproportion in 95.8% of infants with probable CZS	In infected women in the first trimester: Risk of 0.95% in a population with an estimated rate of ZIKV infection of 66%; Prevalence of 55% in Rio de Janeiro. infection in the 3rd trimester: Prevalence: 29% (Rio de Janeiro). In a series of 13 infants with congenital ZIKV infection and progressive microcephaly, more than half of the mothers did not report any symptoms prior to delivery.		In a study of 70 children with microcephaly and laboratory diagnosys of congenital ZIKV infection, 5 (7%) had sensorineural hearing loss.	One study: congenital heart disease was described in 14 of a series of 103 cases (13.6%) in children with CZS.
Santos et al. (2018) [28]		Intracranial calcifitcation: 23 of 23 childen. Frontal lobe: 69% - 78%. Parietal lobe: 83% - 87%. Corticomedullary junction: 53% - 86%. Thalamus: 39% - 43%. Punctate calcification: 72% - 100%. Distributed in the band format: 56% - 75%. Reduction in the constitution of gyri of the severe cerebral cortex: 0.78. Cerebellar hypoplasia: 0.74. Involving only one cerebellar hemisphere : 13%. Brainstem globally hypoplastic: 8.7%. Abnormal hypodensity of the white matter: 1. Diffuse involvement of all the cerebral lobes: 0.87. Basal ganglia calcification: 57% - 65%				
Pomar et al. (2019) [30]	CZS: 4-9% of pregnancies of women infected by ZIKV. Malformations of cortical development: 79-82% of CZS cases. Intraventicular synechiae and periventricular cystic degeneration: 58% of CZS cases. Malformations of the corpus callosum: 71-100%. Vermian hypoplasia: 42% of CZS cases. 21% to 82%. Swallowing disorders and hydramnios: 25%. Partial immobilization or arthrogryposes: 10-25%. Motor abnormalities : 77.3-100% of CZS cases. Adverse outcomes - No signs/complications: 45% of proven infected fetuses/newborn. Adverse outcomes - Mild / moderate signs: 20% of proven infected fetuses/newborn. Adverse outcomes - Severe complications: 21% of proven infected fetuses/newborn. Risk of neurodevelopmental abnormality: 9% of infants born from infected mothers	Brain volume loss: 92%. Ventriculomegaly in CZS: 63.1-92%. Calcifications in CZS: 71-92%	Prevalence of microcephaly in CZS: 33.3-64%	Eye abnormalities: 25% in infants with CZS		

Authors	Malformations / Congenital abnormalities	Brain abnormalities	Microcephaly	Ocular disorders	Auditory disorder - Rate	Cardiovascular damage
Wilder-Smith et al. (2018) [31]	From infected pregnant travelers: Fetuses or infants with birth defects: 6% for asymptomatic women and 6% for symptomatic women with evidence of possible recent ZIKV infection. Zika virus-associated birth defects in infants with ZIKV infection: 10% in completed pregnancies with reported outcomes; 5% in infants with possible ZIKV-associated birth defects from women with confirmed or probably ZIKV infection) (5% among symptomatic and 4% among asymptomatic women). Among 1,508 pregnancies with lab-confirmed ZIKV (5% among symptomatic and 7% among asymptomatic woman). Adverse fetal outcomes: 7% in pregnant women with symptomatic ZIKV infection. Adverse outcomes: 3 of 4 ZIKV infected pregnant women.					
Nithiyanantham et al. (2019) [32]	Prevalence of joint abnormalities: 13.2% in infants of ZIKV-infected mothers	In infants of ZIKV-infected mothers: Ventriculomegaly / hydrocephaly: 21.8% (95% CI,15.2-28.4); Brain calcifications: 42.6% (95% CI, 30.8-54.4)	Prevalence of 3.9% in infants of ZIKV-infected mothers	Prevalence: 4.2% in infants of ZIKV-infected mothers		
Masel et al. (2019) [33]	No association of prior exposure to DENV and fetal imaging abnormalities					
Barbosa et al. (2019) [34]	Microcephaly or neurologic changes: 50.10% on 962 fetus or children studied				Altered OAE varied from 0% to 75%, while altered a-ABR varied from 0% to 29.9%. Among patients who underwent OAE assessments (n=244), 18,4% presented alterations while 25% of microcephaly cases dislpayed alterations. Among the 448 patients who reportedly underwent the first a-ABR test, 15.2% presented alterations. Among three studies that included 102 children with laboratory confirmation of congenital ZIKV infection, 18 (17.6%) had hearing alterations, five in the ABR and 13 in the HINE.	
Minhas et al. (2017) [35]						Cohort with 9 adults positive for ZIKV and no previous cardiac history. 8 of the cases had arrhythmicas and 6 presented heart failure. Of the 8 arrhythmias, 3 were acute atrial fibrillation (two paroxysmal, one persistent), 2 were non-sustained atrial tachycardia, and 2 were ventricular arrhythmias. 5 of the 6 heart failure patients had a low ejection fraction (EF), and one had preserved EF with pre-eclampsia and moderate to severe pericardial effusion.

For peer review only - http://bmjopen.bmj.com/site/about/guidelines.xhtml

Supplementary file 2 - Table 2. Health outcomes - Neurological

Authors	Neurological complications	Epilepsy	Sleep characteristics	GBS
**Krauer et al. (2017) [15]**				74-84% symptomatic ZIKV in GBS cases; ZIKV laboratory-confirmed in GBS cases investigated: 100%
**Paixao et al. (2016) [16]**	French Polynesia outbreak: Amongg patients that visited health care facilities with Zika-like symptoms, 2.3 per 1,000 had neurological complications			In Bahia, Brazil, GBS was diagnosed in 1 of every 1,000 reported ZIKV cases. French Polynesia outbreak: Among patients that visited health care facilities with Zika-like symptoms, 1.3/1,000 ZIKV infections had GBS. ZIKV symptomatic cases when confirmed Among 42 GBS cases, 36% required intensive care and 21% required mechanical ventilation; El salvador: Prevalence of 35% (84 GBS cases in 240 ZIKV infections)
**Simoes et al. (2016) [19]**				In the primary databases consulted, there is only one case report occurred in French Polynesia in which GBS was diagnosed in a patient infected with Zika virus.
**Padilla et al. (2016) [20]**				An analysis of 42 patients who developed GBS during the French Polynesia outbreak estimates the incidence of the disease to be 0.24 per 1000 Zika virus infections. 88% of these patients reported symptoms and 93% of patients showed evidence of recent disease with ZIKV confirmed by the presence of IgM antibodies. Of these patients, 38% required admission to an intensive care unit and 29% required mechanical ventilation.
**Marques et al. (2019) [21]**		Prevalence of epilepsy: 42.2-67% in children with congenital ZIKV. Infantile spasms: 72%, 21.6%. Generalized: 11.8%. Partial: 8.9%. Described as brief jerking spells of flexion and/or extension movements that lasted a few seconds : 21.57%. Focal motor seizures: 21%. Tonic seizures: 4%. Myoclonic seizures: 2%. Myoclonic seizures: 1%.	34.1% (30 in 88 congenital ZIKV-infected children) were defined as poor sleepres and 24% (21 in 88) slept less than 9 hours	

For peer review only - http://bmjopen.bmj.com/site/about/guidelines.xhtml

Authors	Neurological complications	Epilepsy	Sleep characteristics	GBS
**Counotte et al. (2018) [22]**				Prevalence ratio during the ZIKV transmission over pre-outbreak period: 2.0-9.8.
**Haby et al. (2018) [23]**				Prevalence of asymptomatic ZIKV infection in patients with GBS: 0.12
**Wahid et al. (2018) [25]**	A recent study presented neurological disorders in 12 of 16 patients co-infected with ZIKV, chikungunya virus, and dengue virus in Guayaquil, Ecuador. One patients experienced CNS vasculitis, three had GBS whereas, and six patients were diagnosed with meningitis or encephalitis.			About 43% of GBS patients were found to be positive for ZIKV. Another study confirmed ZIKV-linked GBS in 1 of 3 patients.
**Barbi et al. (2018) [27]**				Meta-analysis: 1513 GBS cases in 164,651 ZIKV-infected individuals (0.92%). Estimative the prevalence of GBS to be 1.23% (CI: 95% 1.17%-1.29%) of all ZIKV infection cases in adults. 16 in 38 GBS cases (42%) needed intensive care unit hospitalization (French Polynesia)
**Wachira et al. (2018) [29]**				OR: 59.7 (CI: 95% 10.4 - ∞); Other study: no statistical significance between ZIKV and GBS
**Pomar et al. (2019) [30]**		9-95.5% in congenital ZIKV infection		Prevalence of 1.23% (95% CI, 1.17%-1.29%) in general ZIKV infected-population)
**Wilder-Smith et al. (2018) [31]**				2.15% (2 cases in 93 ZIKV cases recorded in Geosentinel sites)
**Masel et al. (2019) [33]**	No association of prior exposure to DENV and clinical neurological assessment of fetus			No statistically significant difference in patients with GBS with or without prior DENV exposure. No statistical difference in prior DENV exposed patients with or without GBS after ZIKV infection.

Supplementary file 2 - Table 3. Health outcomes – Adverse outcomes

Authors	Death due ZIKV infection	Abortion due to ZIKA / fetal death / perinatal death / neonatal death	Intrauterine growth restrictions - Rate within mother-infant pairs	Abnormal amniotic fluid	Adverse birth outcomes
**Krauer et al. (2017) [15]**		Prevalence in all pregnancy outcomes: Miscarriage 2.5%; intrauterine death or stillbirth 1.1%; termination of pregnancy 5.4%; Neonatal death: 3.2%	28.57% of cases	Rate: 18% of infected pregnant women	
**Paixão et al. (2016) [16]**	In Brazil, 2 deaths of adults were attribuited to Zika and 7 are under investigation by the Ministry of Health; El Salvador (240 ZIKV cases, 2 deaths)				
**Chibueze et al. (2017) [17]**					
**Coelho et al. (2017) [18]**		Miscarriages and perinatal deaths: USA (22% - 2 deaths in 9 ZIKV infected pregnant women), Brazil (6.7% - 9 deaths in 135 ZIKV infected pregnant women), Puerto Rico (3% - 2 deaths in 67 ZIKV infected pregnant women), USA (10.6% - 47 deaths in 442 ZIKV infected pregnant women), French Guiana (4% - 20 deaths in 498 ZIKV infected pregnant women).			
**Simões et al. (2016) [19]**		In Brazil, 1.79% (91/5,079) of microcephaly reported cases, progressed to miscarriage or postpartum death. According to the classification, 64.8% (59/91) remained under investigation; 838% (8/91) were investigated and discarded, and 26.4% (24/91) were investigated and confirmed for microcephaly and/or changes in the CNS.			
**Padilla et al. (2016) [20]**		In 72 women with Zika-positive serology during pregnancy in Brazil, the fetal death rate was 4.8%; Zika infection during 9 pregnancies confirmed by CDC resulted in outcomes of 2 spontaneous abortions and 2 elective abortions.			
**Wahid et al. (2018) [25]**			One study with 88 pregnant women of which 72 were positive for ZIKV and ultrasonography was performed in 42: in utero growth restriction with or without microcephaly (5/42).		
**Pomar et al. (2019) [30]**		14% of proven infected fetuses/newborn	Prevalence of IUGR in CZS: 14%		
**Masel et al. (2019) [33]**		No association of prior exposure to DENV and fetal loss			Occured in 46.4% of those ZIKV infected participants

For peer review only

Supplementary file 3 - Table 1.1. Summary of AMSTAR 2 rating

AMSTAR 2	Krauer et al. [15]	Paixão et al. [16]	Chibueze et al. [17]	Coelho et al. [18]	Simões et al. [19]	Padilla et al. [20]	Marques et al. [21]
**1**	Yes	Yes	Yes	Yes	Yes	No	Yes
**2**	No	No	Partial yes	No	No	No	Yes
**3**	Yes	Yes	Yes	Yes	Yes	Yes	Yes
**4**	Partial yes	Yes	Partial yes	Partial yes	Partial yes	Yes	Partial yes
**5**	Yes	Yes	Yes	Yes	Yes	Yes	Yes
**6**	Yes	No	Yes	Yes	No	No	No
**7**	Yes	Yes	Yes	Yes	No	No	No
**8**	Partial yes	Partial yes	Yes	Partial yes	No	No	No
**9**	No	No	No	No	No	No	No
**10**	Yes	No	Yes	Yes	No	No	Yes
**11**	No	No	No	No	No	No	No
**12**	Yes	No MA conducted	No MA conducted	Yes	No MA conducted	No MA conducted	No MA conducted
**13**	No	No MA conducted	No MA conducted	No	No MA conducted	No MA conducted	No MA conducted
**14**	No	No	No	No	No	No	No
**15**	Yes	Yes	No	Yes	No	No	No
**16**	No	No MA conducted	No MA conducted	No	No MA conducted	No MA conducted	No MA conducted

*MA - Meta-analysis

Questions 2,4,7,9,12 and 14, highlighted, are those of critical domains.

1 - Did the research questions and inclusion criteria for the review include the components of PICO?

2 - Did the report of the review contain an explicit statement that the review methods were established prior to the conduct of the review and did the report justify any significant deviations from the protocol?

3 - Did the review authors explain their selection of the study designs for inclusion in the review?

4 - Did the review authors use a comprehensive literature search strategy?

5 - Did the review authors perform study selection in duplicate?

6 - Did the review authors perform data extraction in duplicate?

7 - Did the review authors provide a list of excluded studies and justify the exclusions?

8 - Did the review authors describe the included studies in adequate detail?

9 - Did the review authors use a satisfactory technique for assessing the risk of bias (RoB) in individual studies that were included in the review?

10 - Did the review authors report on the sources of funding for the studies included in the review?

11 - If meta-analysis was performed did the review authors use appropriate methods for statistical combination of results?

12 - If meta-analysis was performed, did the review authors assess the potential impact of RoB in individual studies on the results of the meta-analysis or other evidence synthesis?

13 - Did the review authors account for RoB in individual studies when interpreting/ discussing the results of the review?

14 - Did the review authors provide a satisfactory explanation for, and discussion of, any heterogeneity observed in the results of the review?

15 - If they performed quantitative synthesis did the review authors carry out an adequate investigation of publication bias (small study bias) and discuss its likely impact on the results of the review?

16 - Did the review authors report any potential sources of conflict of interest, including any funding they received for conducting the review?

For peer review only

Supplementary file 3 - Table 1.2. Summary of AMSTAR 2 rating

AMSTAR 2	Counotte et al. [22]	Haby et al. [23]	Sarwar et al. [24]	Wahid et al. [25]	Soriano-Arandes et al. [26]	Barbi et al. [27]	Santos et al. [28]
**1**	Yes	Yes	Yes	Yes	Yes	Yes	Yes
**2**	Yes	Partial yes	No	No	No	No	No
**3**	No	Yes	No	No	No	No	No
**4**	Partial yes	Partial yes	Partial yes	No	No	No	No
**5**	Yes	No	No	Yes	No	No	No
**6**	Yes	No	No	No	No	Yes	No
**7**	No	Yes	No	No	No	No	No
**8**	Yes	Yes	No	Yes	Yes	Yes	No
**9**	No	No MA conducted	No MA conducted	No MA conducted	No MA conducted	No MA conducted	No
**10**	No	Yes	No	No	No	Yes	No
**11**	No	No	No	No	Yes	No	No
**12**	No MA conducted	Yes	No MA conducted	No MA conducted	No MA conducted	No	No MA conducted
**13**	No MA conducted	No	No MA conducted	No MA conducted	No MA conducted	No	No MA conducted
**14**	No	Yes	No	No	No	No	Yes
**15**	No	Yes	No	No	No	No	No
**16**	No MA conducted	Yes	No MA conducted	No MA conducted	No MA conducted	No	No MA conducted

Questions 2,4,7,9,12 and 14, highlighted, are those of critical domains.

1 - Did the research questions and inclusion criteria for the review include the components of PICO?

2 - Did the report of the review contain an explicit statement that the review methods were established prior to the conduct of the review and did the report justify any significant deviations from the protocol?

3 - Did the review authors explain their selection of the study designs for inclusion in the review?

4 - Did the review authors use a comprehensive literature search strategy?

5 - Did the review authors perform study selection in duplicate?

6 - Did the review authors perform data extraction in duplicate?

7 - Did the review authors provide a list of excluded studies and justify the exclusions?

8 - Did the review authors describe the included studies in adequate detail?

9 - Did the review authors use a satisfactory technique for assessing the risk of bias (RoB) in individual studies that were included in the review?

10 - Did the review authors report on the sources of funding for the studies included in the review?

11 - If meta-analysis was performed did the review authors use appropriate methods for statistical combination of results?

12 - If meta-analysis was performed, did the review authors assess the potential impact of RoB in individual studies on the results of the meta-analysis or other evidence synthesis?

13 - Did the review authors account for RoB in individual studies when interpreting/ discussing the results of the review?

14 - Did the review authors provide a satisfactory explanation for, and discussion of, any heterogeneity observed in the results of the review?

15 - If they performed quantitative synthesis did the review authors carry out an adequate investigation of publication bias (small study bias) and discuss its likely impact on the results of the review?

16 - Did the review authors report any potential sources of conflict of interest, including any funding they received for conducting the review?

For peer review only

Supplementary file 3 - Table 1.3. Summary of AMSTAR 2 rating

AMSTAR 2	Wachira et al. [29]	Pomar et al. [30]	Wilder-Smith et al. [31]	Nithiyanantham et al. [32]	Masel et al. [33]	Barbosa et al. [34]	Minhas et al. [35]
**1**	Yes	Yes	Yes	Yes	Yes	Yes	Yes
**2**	Yes	No	No	No	No	Yes	No
**3**	Yes	No	Yes	Yes	Yes	Yes	Yes
**4**	Partial yes	No	Partial yes	Partial yes	Yes	Partial yes	Partial yes
**5**	Yes	Yes	No	Yes	Yes	Yes	Yes
**6**	Yes	Yes	No	No	Yes	Yes	Yes
**7**	No	No	No	No	No	No	No
**8**	Yes	No	Partial yes	Yes	Yes	Yes	Yes
**9**	No	No	No	No	No	Yes	No
**10**	Yes	No	No	No	No	No	No
**11**	No	No MA conducted	No MA conducted	No	No MA conducted	No MA conducted	No MA conducted
**12**	No MA conducted	No MA conducted	No MA conducted	No	No MA conducted	No MA conducted	No MA conducted
**13**	No MA conducted	No	No	No	No	No	No
**14**	Yes	No	Yes	Yes	No	Yes	No
**15**	Yes	No MA conducted	No MA conducted	Yes	No MA conducted	No MA conducted	No MA conducted
**16**	No MA conducted	Yes	Yes	Yes	Yes	Yes	Yes

Questions 2,4,7,9,12 and 14, highlighted, are those of critical domains.
1 - Did the research questions and inclusion criteria for the review include the components of PICO?
2 - Did the report of the review contain an explicit statement that the review methods were established prior to the conduct of the review and did the report justify any significant deviations from the protocol?
3 - Did the review authors explain their selection of the study designs for inclusion in the review?
4 - Did the review authors use a comprehensive literature search strategy?
5 - Did the review authors perform study selection in duplicate?
6 - Did the review authors perform data extraction in duplicate?
7 - Did the review authors provide a list of excluded studies and justify the exclusions?
8 - Did the review authors describe the included studies in adequate detail?
9 - Did the review authors use a satisfactory technique for assessing the risk of bias (RoB) in individual studies that were included in the review?
10 - Did the review authors report on the sources of funding for the studies included in the review?
11 - If meta-analysis was performed did the review authors use appropriate methods for statistical combination of results?
12 - If meta-analysis was performed, did the review authors assess the potential impact of RoB in individual studies on the results of the meta-analysis or other evidence synthesis?
13 - Did the review authors account for RoB in individual studies when interpreting/ discussing the results of the review?
14 - Did the review authors provide a satisfactory explanation for, and discussion of, any heterogeneity observed in the results of the review?
15 - If they performed quantitative synthesis did the review authors carry out an adequate investigation of publication bias (small study bias) and discuss its likely impact on the results of the review?
16 - Did the review authors report any potential sources of conflict of interest, including any funding they received for conducting the review?

For peer review only

PRISMA 2009 Checklist

Section/topic	#	Checklist item	Reported on page #
**TITLE**			
Title	1	Identify the report as a systematic review, meta-analysis, or both.	1
**ABSTRACT**			
Structured summary	2	Provide a structured summary including, as applicable: background; objectives; data sources; study eligibility criteria, participants, and interventions; study appraisal and synthesis methods; results; limitations; conclusions and implications of key findings; systematic review registration number.	2
**INTRODUCTION**			
Rationale	3	Describe the rationale for the review in the context of what is already known.	3
Objectives	4	Provide an explicit statement of questions being addressed with reference to participants, interventions, comparisons, outcomes, and study design (PICOS).	3
**METHODS**			
Protocol and registration	5	Indicate if a review protocol exists, if and where it can be accessed (e.g., Web address), and, if available, provide registration information including registration number.	3,4
Eligibility criteria	6	Specify study characteristics (e.g., PICOS, length of follow-up) and report characteristics (e.g., years considered, language, publication status) used as criteria for eligibility, giving rationale.	3,4
Information sources	7	Describe all information sources (e.g., databases with dates of coverage, contact with study authors to identify additional studies) in the search and date last searched.	3,4
Search	8	Present full electronic search strategy for at least one database, including any limits used, such that it could be repeated.	3
Study selection	9	State the process for selecting studies (i.e., screening, eligibility, included in systematic review, and, if applicable, included in the meta-analysis).	3,4
Data collection process	10	Describe method of data extraction from reports (e.g., piloted forms, independently, in duplicate) and any processes for obtaining and confirming data from investigators.	3,4
Data items	11	List and define all variables for which data were sought (e.g., PICOS, funding sources) and any assumptions and simplifications made.	
Risk of bias in individual studies	12	Describe methods used for assessing risk of bias of individual studies (including specification of whether this was done at the study or outcome level), and how this information is to be used in any data synthesis.	4
Summary measures	13	State the principal summary measures (e.g., risk ratio, difference in means).	5-6
Synthesis of results	14	Describe the methods of handling data and combining results of studies, if done, including measures of consistency (e.g., I^2) for each meta-analysis.	

For peer review only - http://bmjopen.bmj.com/site/about/guidelines.xhtml

PRISMA 2009 Checklist

Section/topic	#	Checklist item	Reported on page #
Risk of bias across studies	15	Specify any assessment of risk of bias that may affect the cumulative evidence (e.g., publication bias, selective reporting within studies).	
Additional analyses	16	Describe methods of additional analyses (e.g., sensitivity or subgroup analyses, meta-regression), if done, indicating which were pre-specified.	
**RESULTS**			
Study selection	17	Give numbers of studies screened, assessed for eligibility, and included in the review, with reasons for exclusions at each stage, ideally with a flow diagram.	4
Study characteristics	18	For each study, present characteristics for which data were extracted (e.g., study size, PICOS, follow-up period) and provide the citations.	5
Risk of bias within studies	19	Present data on risk of bias of each study and, if available, any outcome level assessment (see item 12).	5
Results of individual studies	20	For all outcomes considered (benefits or harms), present, for each study: (a) simple summary data for each intervention group (b) effect estimates and confidence intervals, ideally with a forest plot.	
Synthesis of results	21	Present results of each meta-analysis done, including confidence intervals and measures of consistency.	
Risk of bias across studies	22	Present results of any assessment of risk of bias across studies (see Item 15).	
Additional analysis	23	Give results of additional analyses, if done (e.g., sensitivity or subgroup analyses, meta-regression [see Item 16]).	
**DISCUSSION**			
Summary of evidence	24	Summarize the main findings including the strength of evidence for each main outcome; consider their relevance to key groups (e.g., healthcare providers, users, and policy makers).	6-8
Limitations	25	Discuss limitations at study and outcome level (e.g., risk of bias), and at review-level (e.g., incomplete retrieval of identified research, reporting bias).	8
Conclusions	26	Provide a general interpretation of the results in the context of other evidence, and implications for future research.	9
**FUNDING**			
Funding	27	Describe sources of funding for the systematic review and other support (e.g., supply of data); role of funders for the systematic review.	9

From: Moher D, Liberati A, Tetzlaff J, Altman DG, The PRISMA Group (2009). Preferred Reporting Items for Systematic Reviews and Meta-Analyses: The PRISMA Statement. PLoS Med 6(7): e1000097. doi:10.1371/journal.pmed1000097

For more information, visit: **www.prisma-statement.org**.

For peer review only - http://bmjopen.bmj.com/site/about/guidelines.xhtml
